# Contraceptive Use in Adolescent Girls and Adult Women in Low- and Middle-Income Countries

**DOI:** 10.1001/jamanetworkopen.2019.21437

**Published:** 2020-02-19

**Authors:** Zhihui Li, George Patton, Farnaz Sabet, Zhiying Zhou, S. V. Subramanian, Chunling Lu

**Affiliations:** 1 Department of Global Health and Population, Department of Social and Behavioral Sciences, Harvard T.H. Chan School of Public Health, Boston, Massachusetts; 2 University of Melbourne, Parkville, Victoria, Australia; 3 Murdoch Children’s Research Institute, Parkville, Victoria, Australia; 4 Xi’an Jiaotong University School of Public Policy and Administration, Xi’an, Shaanxi, China; 5 Department of Social and Behavioral Sciences, Harvard T.H. Chan School of Public Health, Boston, Massachusetts; 6 Brigham and Women’s Hospital, Harvard Medical School, Boston, Massachusetts

## Abstract

**Importance:**

Preventing unwanted pregnancy through adequate use of contraceptives among adolescent girls is essential for improving maternal and child health and social well-being.

**Objective:**

To estimate the levels and trends of contraceptive use in adolescent girls (age 15-19 years) compared with adult women (age 20-34 years).

**Design, Setting, and Participants:**

This survey study used data from 261 Demographic and Health Surveys or Multiple Cluster Indicator Surveys conducted in 103 low- and middle-income countries between 2000 and 2017. Modern contraceptive use in 90 countries and unmet need for family planning in 73 countries for adolescent girls and adult women were assessed at country and aggregate levels. Data analyses for this study were conducted between March 2019 and December 2019.

**Main Outcomes and Measures:**

Modern contraceptive use and unmet need for family planning, estimated between adolescent girls and adult women for absolute inequality and relative inequality (defined as the ratio in service use between adult women and adolescent girls). Inequalities by wealth and place of residence in each age group were also assessed.

**Results:**

A total of 832 673 adolescent girls and 2 156 268 adult women were included in the analysis. Adolescent girls were significantly less likely to use modern contraceptives (31.6% [95% CI, 30.3% to 32.8%] vs 43.5% [95% CI, 42.4% to 44.7%]; absolute inequality, 11.9 percentage points [PPs] [95% CI, 11.7 to 12.1 PPs]; relative inequality, 1.38 PPs [95% CI, 1.36 to 1.40 PPs]) and more likely to have an unmet need for family planning (50.8% [95% CI, 49.0% to 52.5%] vs 36.4% [95% CI, 35.9% to 36.8%]; absolute inequality, −14.4 PPs [95% CI, −14.8 to −14.0 PPs]; relative inequality, 0.72 PPs [95% CI, 0.69 to 0.75 PPs]). Contraceptive use generally improved over time (aggregate-level use of contraceptives increased from 17.8% [95% CI, 16.6% to 19.0%] in 2000-2006 to 27.2% [95% CI, 26.6% to 27.8%] in 2013-2017 for adolescents and from 30.9% [95% CI, 29.8% to 32.0%] in 2000-2006 to 40.3% [95% CI, 39.8% to 40.8%] in 2013-2017 for adult women), but inequalities between adolescents and adult women persisted. Aggregate-level prevalence of unmet need for family planning decreased only among adult women (from 45.8% [95% CI, 44.9% to 46.7%] in 2000-2006 to 38.0% [95% CI, 37.3% to 38.7%] in 2013-2017). For adolescents, the prevalence remained at approximately 52% over time. The inequality between the 2 age groups therefore significantly increased from 7.5 PPs (95% CI, 6.5 to 8.4 PPs) in 2000 to 2006 to 14.0 PPs (95% CI, 13.4 to 14.6 PPs) in 2013 to 2017. An increasing gap between adolescent girls and adult women was found in 18 countries for modern contraceptive use and in 20 countries for unmet need for family planning. In India, for example, both age groups had increased percentage of unmet need for family planning over time, from 16.2% (95% CI, 15.9% to 16.6%) in 2006 to 29.8% (95% CI, 29.6% to 30.1%) in 2015 for adult women and from 23.9% (95% CI, 23.0% to 24.9%) to 64.5% (95% CI, 63.3% to 65.7%) among adolescents. Thus, inequality increased from 7.7 PPs (95% CI, 7.2 to 8.2 PPs) in 2006 to 34.7 PPs (95% CI, 34.2 to 35.1 PPs) in 2015.

**Conclusions and Relevance:**

Despite a growing focus on contraception in the past decade with initiatives such as Family Planning 2020, adolescent girls continue to lag behind adult women in contraceptive use. There is a pressing need to develop age-appropriate strategies for increasing access and maintaining contraceptive use in sexually active adolescent girls.

## Key Points

**Question:**

Do adolescent girls (age 15-19 years) have similar levels of contraceptive use as adult women (age 20-34 years) in low- and middle-income countries?

**Findings:**

In this survey study using data from 261 Demographic and Health Surveys or Multiple Cluster Indicator Surveys for 103 low-and middle-income countries between 2000 and 2017, contraceptive use increased over time among both adolescent girls and adult women, but inequality in use by age persisted and, in some countries, increased.

**Meaning:**

Targeted strategies are likely to be required for adolescent girls to have equivalent opportunities to use contraceptives as older women.

## Teaser

This survey study compares rates of contraceptives use by adolescent girls and adult women in low- and middle-income countries (LMICs).

## Introduction

Maternal and child deaths remain global health priorities. The era of the United Nations Millennium Development Goals witnessed great progress in reducing maternal and child mortality rates between 2000 and 2015.^1,2,3^ The United Nations Sustainable Development Goals have now set an ambitious target to reduce the global maternal mortality ratio to less than 70 deaths per 100 000 live births and mortality rates of children younger than 5 years to no more than 25 deaths per 1000 live births by 2030.^4^ Adolescents younger than 20 years are a priority in meeting these targets as they face higher risks for both mothers and infants. It is well documented that, compared with pregnancy in adult women, adolescent pregnancy is associated with a range of adverse outcomes, including increased neonatal mortality; higher incidence of low birth weight, prematurity, and developmental disabilities; and greater risks for pregnancy-related complications and deaths.^1,2,5,6,7,8^

It is estimated that approximately half of pregnancies in girls aged 15 to 19 years are unintended.^5^ One consequence is unsafe abortion in 3.9 million girls annually.^5,9,10^ According to the World Health Organization, meeting the unmet need for modern contraception in adolescents would reduce unintended pregnancies among this age group by 6.0 million annually, leading to 2.1 million fewer unplanned births, 3.2 million fewer abortions, and 5600 fewer maternal deaths.^5^

Meeting adolescents’ contraceptive need has been considered seminal in achieving the Family Planning 2020 (FP2020) goal in the 69 poorest countries^11^ (providing 120 million more women and girls access to contraceptives) and is also addressed in the Every Woman Every Child Global Strategy.^12^ Yet research into age-related differences in contraceptive use has been limited. Two studies^13,14^ reported contraceptive use by maternal age in 75 Countdown countries (countries where >95% of all maternal and child deaths occur) and 84 low- and middle-income countries (LMICs), but did not assess the progress in reducing inequalities between age groups. In this study, we drew data from across 103 LMICs to generate comprehensive analyses on levels and trends of contraceptive use and unmet need among adolescent girls (age 15-19 years) and adult women (age 20-34 years) in LMICs, with analyses of inequalities between the 2 age groups.

## Methods

### Data Sources

We adopted data from 261 Demographic and Health Surveys (DHS) and Multiple Cluster Indicator Surveys (MICS) conducted between 2000 and 2017 in 103 LMICs (eTable 1 in the Supplement). All surveys with available information on contraceptive use or family planning need were included. The DHS and MICS surveys are country led and typically have response rates greater than 90%.^15,16^ According to the World Health Organization, DHS and MICS surveys are highly comparable because the survey design and implementation quality are sufficiently similar between DHS and MICS across countries and over time.^17^ The DHS and MICS usually use a 2-stage stratified cluster sampling design. In the first stage, enumeration areas are drawn from census files, and in the second stage, a sample of households are selected from a list of households in each enumeration area.^18,19^ For each sampled household, women aged between 15 and 49 years are surveyed regarding their contraceptive use and need during the 3 years prior to the survey.

In this study, we only included women younger than 35 years because their sexuality and reproductive capacity varies from women aged 35 years or older, which may be associated with a different pattern of contraceptive use.^20,21^ A total of 832 673 adolescents (defined as girls aged 15-19 years^22^) and 2 156 268 adult women (defined as women aged 20-34 years^22^) had information on contraceptive use or unmet need for family planning and were therefore included in our analyses.

This study used publicly accessible secondary data and therefore obtained an exemption from the institutional review board at Harvard University. Reporting of this study follows the American Association for Public Opinion Research (AAPOR) reporting guideline.

### Indicators of Contraceptive Use

We focused on 2 indicators in the surveys: modern contraceptive use and unmet need for family planning. Modern contraceptive use is defined as whether a woman (sexually active) or her partner is currently using at least 1 modern method of contraception. Modern methods of contraception include female and male sterilization, oral hormonal pills, intrauterine devices, male condoms, injectables, implants, vaginal barrier methods, female condoms, and emergency contraception. Family planning need is unmet if a woman who is sexually active and fecund wishes to stop or delay childbearing and is not currently using any modern method of contraception.

### Inequality Measurements

We adopted 2 commonly used measurements^23^ to estimate the inequalities by age. *Absolute inequality* refers to the difference in service use between adult women and adolescents. For modern contraceptive use, a positive value indicates that adult women used more modern contraceptive than adolescents; for unmet need for family planning, a positive value indicates adults were more likely to have family planning needs unsatisfied. *Relative inequality* is defined as the ratio in service use between adult women and adolescents. Adult women fare better than adolescents if for modern contraceptive use the value is greater than 1, and for unmet family planning the value is less than 1.

In each age group, we also assessed absolute inequalities by wealth and residence. We then made comparisons of inequalities by wealth and residence between the 2 age groups. To assess wealth-based inequalities, we adopted the slope index of inequality based on a generalized linear model with logit link.^24^ Specifically, we ranked from the most disadvantaged subgroup (at rank 0) to the most advantaged subgroup (at rank 1) using the wealth index constructed by DHS and MICS, accounting for the proportional distribution of the population within each subgroup. Based on the regression model, we calculated the absolute difference in use between the 2 extremes of the wealth distribution (rank 0 and 1).^25,26^ We produced residence-based inequalities for each age group by taking the difference between the urban and rural groups. See eTable 2 in the Supplement for details.

### Statistical Analysis

Our study investigated use and inequality in the 2 indicators at both aggregate and country levels. For analyses at both levels, we first generated a snapshot from the latest surveys and then examined the changes in use and inequality over time. We followed the DHS and MICS guidelines^27,28^ and adjusted for sample design, taking into account sampling weight, clustering, and stratification variables provided by DHS and MICS.

To obtain estimates of the latest surveys at the country level, we kept countries with the latest surveys conducted since 2010. Among the 103 countries involved in the study, 90 countries collected data in 2010 or after on modern contraceptive use and 73 countries collected data on family planning need. For both indicators, the mean year of the latest surveys since 2010 was 2012 and the median was 2013, with an interquartile range between 2011 and 2014. We first generated point estimates of service use for both adolescents and adult women and then calculated the difference and ratio measures between the 2 age groups. At the aggregate level, we followed previous practice^29^ by pooling samples from various countries together and reweighting the observations by the country’s population size. The other procedure was the same as at the country level. Consistent with previous studies,^30,31,32^ we used the bootstrap method at both the aggregate and country level to produce standard errors for point estimates, absolute inequality, and relative inequality.

To assess country-level changes in use and inequality over time, we excluded countries with the earliest survey conducted too close to the latest survey (<5 years apart) to ensure adequate time between the 2 surveys. We identified 72 countries for trend analyses on modern contraceptive use and 55 countries on unmet need for family planning. For both indicators, the latest and the earliest surveys were a mean of 9 years apart, with a median of 9 years and an interquartile range between 7 and 10 years. Using the same method as in analyses for the most recent estimates, we generated point estimates, inequalities, and their 95% confidence intervals for each country in each round. We checked the significance of changes between rounds using a bootstrap test (drawing bootstrap samples from each round and computing the difference between means, then repeating the process 1000 times to generate the *P *value for the difference based on bootstrap replicates).^33^ As the number of years between the 2 rounds are not the same for different countries, we also calculated the absolute annual pace of change as the total change in inequalities divided by the number of years between the latest and the earliest surveys. In terms of aggregate-level trend analysis, we divided the survey years into 3 rounds according to the dates of survey implementation: round 1 from 2000 to 2006, round 2 from 2007 to 2012, and round 3 from 2013 to 2017. For countries with multiple years of data available within 1 survey round, we adopted the most recent one. Of the 103 countries, 43 had data in all 3 rounds enabling us to analyze trends of inequality in the 2 indicators over time in these countries. Similar to the country-level analyses, when examining the aggregate-level changes in contraceptive use and inequality status over time, we generated point estimates, absolute inequality, and relative inequality for each round and checked the significance of changes with a bootstrap test.^33^

For wealth and residence-based inequality within each age group, we also used generalized linear regression to generate 95% confidence intervals for the slope index of inequality and bootstrap method to generate the 95% confidence intervals for disparity between rural and urban areas.

We used Stata software version 14.2 (StataCorp) for all analyses. We ascertained statistical significance with 95% confidence intervals.

## Results

Using data from 261 DHS or MICS surveys conducted in 103 LMICs between 2000 and 2017, we included a total of 832 673 adolescent girls and 2 156 268 adult women in the analysis.

### Aggregate-Level Inequalities of Contraceptive Use and Unmet Need in the Latest Surveys Since 2010

Overall, 41% of women (aged 15-34 years) in 90 countries with available data used modern contraceptives and 38% of those in 73 countries with available data had an unmet need for family planning (eTable 3 in the Supplement). The use of modern contraceptives was higher among adult women by 11.9 percentage points (PPs) (95% CI, 11.7 to 12.1 PPs) (31.6% [95% CI, 30.3% to 32.8%] for adolescents and 43.5% [95% CI, 42.4% to 44.7%] for adult women). The prevalence of unmet need for family planning was substantially higher among adolescents (50.8% [95% CI, 49.0% to 52.5%]) compared with adult women (36.4% [95% CI, 35.9% to 36.8%]), with an absolute difference of −14.4 PPs (95% CI, −14.8 to −14.0 PPs). The results by regions, income groups, and FP2020 groups align with the aggregate-level results. Relative inequalities showed consistent results (Table). Inequalities in contraceptive use and unmet need by wealth or place of residence among adolescents was not significantly different compared with adult women, suggesting that income status and place of residence played similar roles in both groups and are unlikely to explain age-related inequalities (eFigure 1 in the Supplement shows aggregate-level inequalities by wealth and place of residence, and eTable 4, eTable 5, eTable 6, and eTable 7 in the Supplement include country-level inequalities by wealth and place of residence).

**Table.** Estimates of Contraception Use and Unmet Need by Maternal Age and Inequalities Between the 2 Age Groups Using the Latest Available Survey Data Since 2010 for 103 Countries

Category	No. of Countries	Service Use, % (95% CI)		Inequality	
		15-19 y	20-34 y	Absolute, Percentage Points	Relative^a^
**Modern Contraceptive Use**					
All available countries	90	31.6 (30.3 to 32.8)	43.5 (42.2 to 44.7)	11.9 (11.7 to 12.1)	1.38 (1.36 to 1.40)
World Health Organization regions					
Africa	40	10.1 (9.4 to 10.7)	18.6 (17.7 to 19.5)	8.6 (8.4 to 8.7)	1.84 (1.81 to 1.87)
Americas	16	46.0 (44.7 to 47.4)	60.1 (58.6 to 61.6)	14.1 (13.8 to 14.4)	1.31 (1.27 to 1.34)
Eastern Mediterranean	9	12.4 (11.1 to 13.7)	38.9 (36.5 to 41.4)	26.5 (26.1 to 26.9)	3.14 (3.09 to 3.18)
Europe	12	22.0 (20.3 to 23.6)	43.1 (41.4 to 44.8)	21.2 (19.9 to 22.4)	1.96 (1.92 to 2.00)
Southeast Asia	8	44.9 (43.9 to 46.0)	56.3 (55.5 to 57.2)	11.4 (10.7 to 12.1)	1.25 (1.19 to 1.32)
Western Pacific	5	22.3 (20.5 to 24.0)	48.9 (47.3 to 50.4)	26.6 (25.4 to 27.8)	2.19 (2.15 to 2.23)
Country income class					
Low	28	26.7 (25.6 to 27.8)	39.4 (38.1 to 40.7)	12.7 (12.5 to 12.9)	1.48 (1.44 to 1.51)
Lower-middle	36	28.1 (26.8 to 29.3)	42.2 (40.9 to 43.5)	14.1 (13.4 to 14.9)	1.50 (1.45 to 1.55)
Upper-middle	26	44.9 (42.9 to 46.9)	58.7 (57.0 to 60.5)	13.8 (13.2 to 14.4)	1.31 (1.28 to 1.33)
SSA category					
Non-SSA	51	37.2 (36.3 to 38.1)	53.0 (52.3 to 53.6)	15.8 (15.5 to 16.1)	1.42 (1.38 to 1.47)
SSA	39	9.8 (9.2 to 10.5)	18.0 (17.1 to 18.9)	8.2 (7.8 to 8.6)	1.84 (1.81 to 1.86)
FP2020 group^b^					
Non-FP2020 countries	33	43.4 (42.6 to 44.2)	55.4 (54.7 to 56.1)	12.0 (11.3 to 12.8)	1.27 (1.22 to 1.32)
FP2020 countries	57	27.6 (27.2 to 28.0)	38.8 (38.3 to 39.2)	11.2 (10.7 to 11.7)	1.41 (1.38 to 1.44)
**Unmet Need for Family Planning (%)**					
All available countries	73	50.8 (49.0 to 52.5)	36.4 (35.9 to 36.8)	−14.4 (−14.8 to −14.0)	0.72 (0.69 to 0.75)
World Health Organization regions					
Africa	37	71.6 (70.4 to 72.7)	57.3 (56.1 to 58.5)	−14.3 (−14.8 to −13.8)	0.80 (0.77 to 0.83)
Americas	15	27.6 (25.9 to 29.3)	20.2 (18.8 to 21.6)	−7.4 (−8.1 to −6.7)	0.73 (0.69 to 0.77)
Eastern Mediterranean	6	49.4 (45.1 to 53.8)	37.2 (34.0 to 40.4)	−12.2 (−13.0 to −11.4)	0.75 (0.71 to 0.80)
Europe	5	42.4 (38.9 to 45.9)	25.7 (23.1 to 28.4)	−16.7 (−18.2 to −15.1)	0.61 (0.56 to 0.65)
Southeast Asia	7	35.9 (32.7 to 39.1)	21.8 (20.6 to 23.0)	−14.1 (−15.3 to −12.8)	0.61 (0.54 to 0.68)
Western Pacific	3	47.8 (45.6 to 50.0)	28.6 (26.9 to 30.3)	−19.2 (−20.3 to −18.1)	0.60 (0.56 to 0.64)
Country-income class					
Low	25	47.3 (45.6 to 49.0)	37.6 (36.1 to 39.1)	−9.7 (−10.3 to −9.0)	0.79 (0.75 to 0.84)
Lower-middle	31	53.1 (51.2 to 55.0)	33.9 (32.5 to 35.4)	−19.2 (−20.2 to −18.1)	0.64 (0.58 to 0.69)
Upper-middle	17	29.5 (27.2 to 31.8)	20.8 (18.9 to 22.8)	−8.7 (−9.7 to −7.6)	0.71 (0.67 to 0.74)
SSA category					
Non-SSA	37	38.1 (36.7 to 39.5)	24.4 (23.6 to 25.1)	−13.8 (−14.6 to −12.9)	0.64 (0.59 to 0.69)
SSA	36	72.1 (70.9 to 73.2)	58.0 (57.0 to 59.0)	−14.5 (−15.0 to −14.0)	0.80 (0.77 to 0.83)
FP2020 group^b^					
Non-FP2020 countries	23	31.7 (30.3 to 33.1)	20.3 (18.4 to 22.2)	−11.4 (−12.3 to −10.4)	0.64 (0.59 to 0.69)
FP2020 countries	50	53.9 (53.3 to 54.5)	38.5 (37.2 to 39.8)	−15.4 (−16.1 to −14.7)	0.71 (0.68 to 0.74)

Abbreviations: FP2020, Family Planning 2020; SSA, sub-Saharan African countries.

^a^ Relative inequality was defined as the ratio in service use between adult women and adolescents.

^b^ A list of FP2020 prioritized countries is available at the FP2020 website.^11^

### Aggregate-Level Inequalities of Contraceptive Use and Unmet Need for Countries With Data Available Over the 3 Rounds

**Figure 1.**
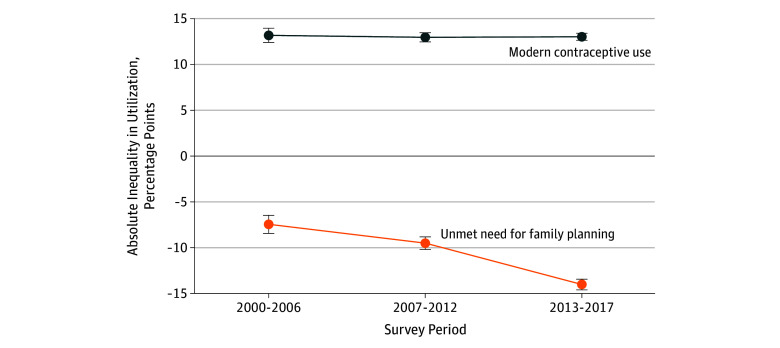
Absolute Inequality in Contraception Use and Unmet Need Between Adult Women 20 to 34 Years Old and Adolescents 15 to 19 Years Old Over 3 Survey Rounds in 43 Countries With Complete Data We kept 43 countries with at least 1 survey taken in each of the 3 rounds to ensure we studied the same cluster of countries in each survey round. The 43 countries were Albania, Armenia, Bangladesh, Belize, Benin, Burundi, Cambodia, Cameroon, Chad, Colombia, Congo, Cuba, Côte d'Ivoire, Dominican Republic, Egypt, Ethiopia, Ghana, Guinea, Guinea-Bissau, Guyana, Haiti, Indonesia, Kazakhstan, Kenya, Kyrgyzstan, Lesotho, Malawi, Mali, Mongolia, Nepal, Nigeria, Philippines, Rwanda, Senegal, Serbia, Sierra Leone, Tajikistan, Tanzania, Thailand, Togo, Uganda, Vietnam, and Zimbabwe. Error bars indicate 95% confidence intervals.

Over the 3 survey rounds, while there was an increase in aggregate-level modern contraceptive use in both age groups (from 17.8% [95% CI, 16.6%-19.0%] in 2000-2006 to 27.2% [95% CI, 26.6%-27.8%] in 2013-2017 for adolescents; and from 30.9% [95% CI, 29.8%-32.0%] in 2000-2006 to 40.3% [95% CI, 39.8%-40.8%] in 2013-2017 for adult women) (eTable 8 in the Supplement), aggregate-level absolute inequalities were significantly positive in all 3 rounds, suggesting that the use of modern contraceptives was higher among adult women compared with adolescents. The inequality level did not change significantly over time (Figure 1).

Aggregate-level prevalence of unmet need for family planning decreased only among adult women (from 45.8% [95% CI, 44.9%-46.7%] in 2000-2006 to 38.0% [95% CI, 37.3%-38.7%] in 2013-2017). Regarding adolescents, the prevalence remained at approximately 52% over time (eTable 8 in the Supplement). The inequality between the 2 age groups therefore significantly increased from 7.5 PPs (95% CI, 6.5-8.4 PPs) in 2000 to 2006 to 14.0 PPs (95% CI, 13.4-14.6 PPs) in 2013 to 2017 (eTable 8 in the Supplement). Similar trends in relative inequalities are presented in eTable 8 in the Supplement.

We stratified the countries by FP2020 group in eTable 9 in the Supplement. We found that aggregate-level inequality in modern contraceptive use remained unchanged for the FP2020 group (33 countries), but decreased significantly for non-FP2020 group (10 countries). Inequality in unmet need for family planning significantly worsened over time for the FP2020 group.

### Country-Level Inequalities of Contraceptive Use and Unmet Need in the Latest Surveys Since 2010

**Figure 2.**
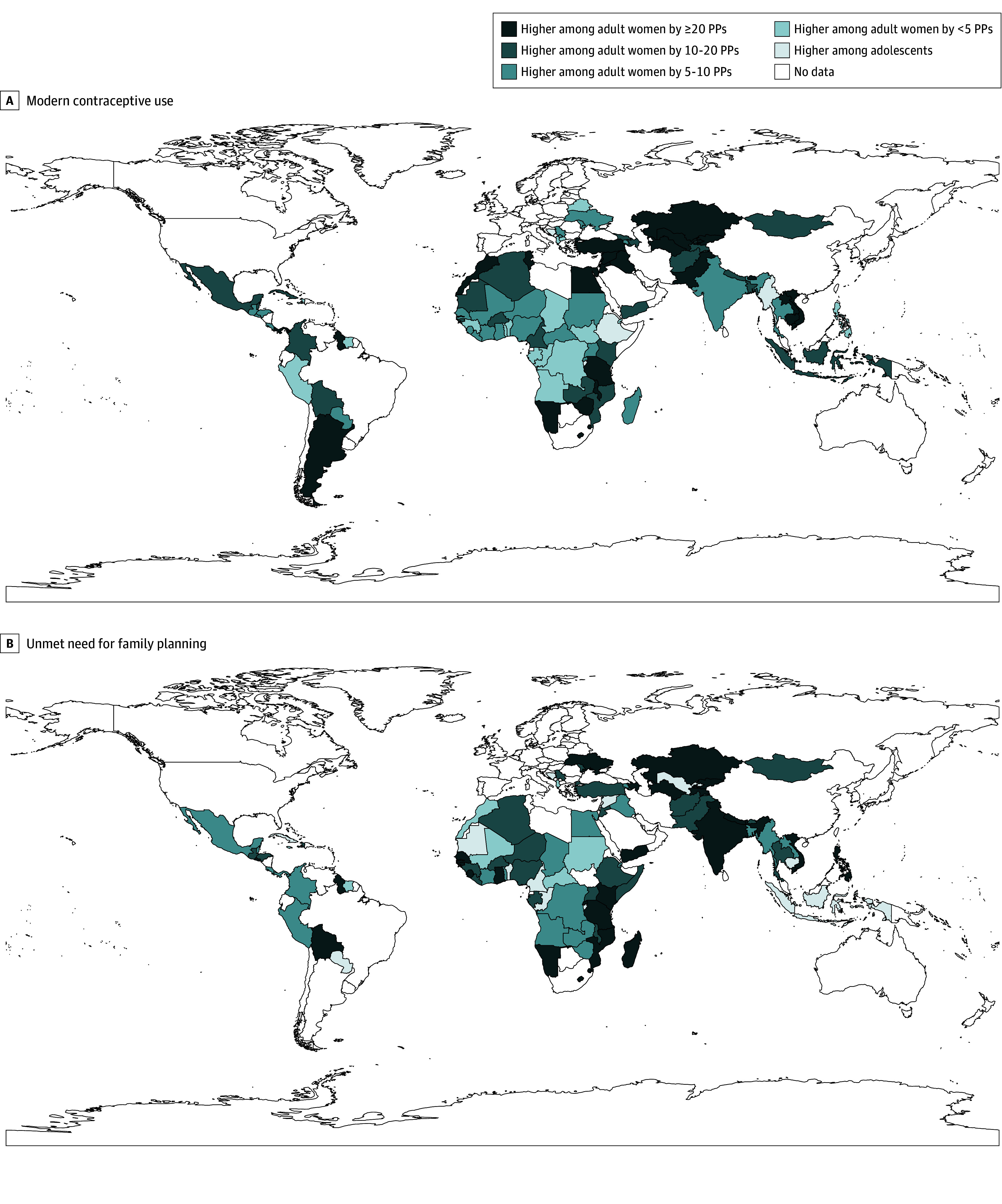
Absolute Inequality in Percentage Points (PPs) in Contraception Use and Unmet Need Between Adult Women and Adolescents for the Most Recent Years of Data Available

Among 90 countries with available data on modern contraceptive use from 2010, 4 had a use of more than 70% among women aged 15 to 34 years (Colombia, Costa Rica, Cuba, and Thailand), and all of which were non-FP2020 upper-middle-income countries. Modern contraceptive use was less than 30% for women aged 15 to 34 years in 37 countries, including 29 FP2020 countries. Of the 90 countries, 77 countries had greater use among adult women. Burundi was the only country where modern contraceptive use was higher among adolescents (eTable 10 in the Supplement; Figure 2A).

Among the 72 countries with available data on unmet need for family planning since 2010, 11 countries had more than 70% of women aged 15 to 34 years not meeting their need for family planning, and 10 of them were FP2020 countries. Adolescents in 58 countries (including 39 FP2020 countries) had a significantly higher level of unmet need for family planning than adult women. The 58 countries spread across various regions and income groups. Only 5 countries (4 FP2020 countries [Burundi, Cambodia, Cameroon, and Mauritania] and 1 non-FP2020 country [Cuba]) had a higher level of unmet need for family planning among adult women (eTable 11 in the Supplement; Figure 2B). The results using relative inequality were largely consistent with those for absolute inequality (eFigure 2 in the Supplement).

### Country-Level Inequalities in Contraceptive Use and Unmet Need Over Time

Among the 72 countries with multiple surveys on modern contraceptive use, 16 countries (including 13 FP2020 countries) increased use among women aged 15 to 34 years by more than 10 PPs, with the largest increase in Rwanda at 37 PPs. Four countries had a reduction in use by more than 10 PPs (Albania, Mauritania, Mongolia [an FP2020 country], and Serbia) (eTable 10 in the Supplement).

**Figure 3.**
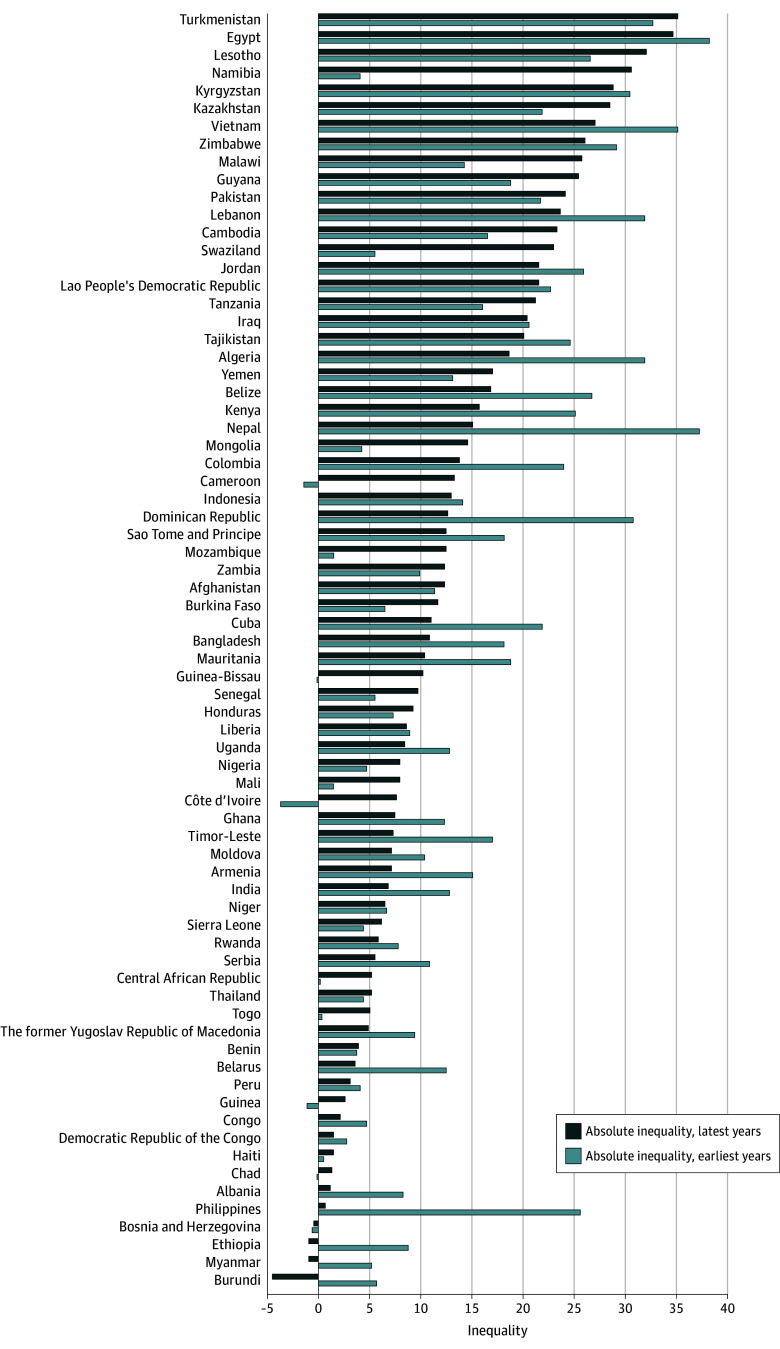
Absolute Inequality in Percentage Points in Modern Contraceptive Use Between Adult Women and Adolescents, Latest Data vs Earliest Data

Of 72 countries, 18 from various regions and income groups had a growing inequality in modern contraceptive use between adolescent girls and adult women, mostly due to a greater increase in use among adult women where use in both groups increased; or a slower decrease in adult women where the use in both groups decreased. Among the 18 countries, 14 were in the FP2020 group. Namibia had the largest deterioration in equity: its absolute inequality increased from 4.0 PPs (95% CI, 1.6-6.4 PPs) in 2007 (55.9% [95% CI, 51.9%-59.9%] for adolescents vs 59.9% [95% CI, 58.1%-61.7%] for adult women) to 30.6 PPs (95% CI, 26.0-35.1 PPs) in 2013 (32.2% [95% CI, 22.5%-41.9%] for adolescents vs 62.8% [95% CI, 61.0%-64.6%] for adult women), with an annual change of 4.4 PPs (Figure 3; eTable 12 in the Supplement).

Twenty-seven countries (including 15 FP2020 countries) from various regions and income groups had significantly reduced inequalities between the 2 age groups, mostly because modern contraceptive use increased faster among adolescents. The Philippines, Nepal, and the Dominican Republic experienced the largest improvement in the equality status over time; both the Philippines and Nepal were FP2020 countries (Figure 3; eTable 12 in the Supplement).

Among the 55 countries with data available in multiple survey rounds, unmet need for family planning decreased in 13 countries by more than 20 PPs; 12 of these were FP2020 countries (eg, Cambodia, Ethiopia, and Kenya). Conversely, 6 FP2020 countries (Benin, Chad, Congo, Mozambique, Mongolia, and Nigeria) had increases in unmet need for family planning of more than 10 PPs, suggesting more women had their need for family planning unsatisfied over time (eTable 11 in the Supplement).

**Figure 4.**
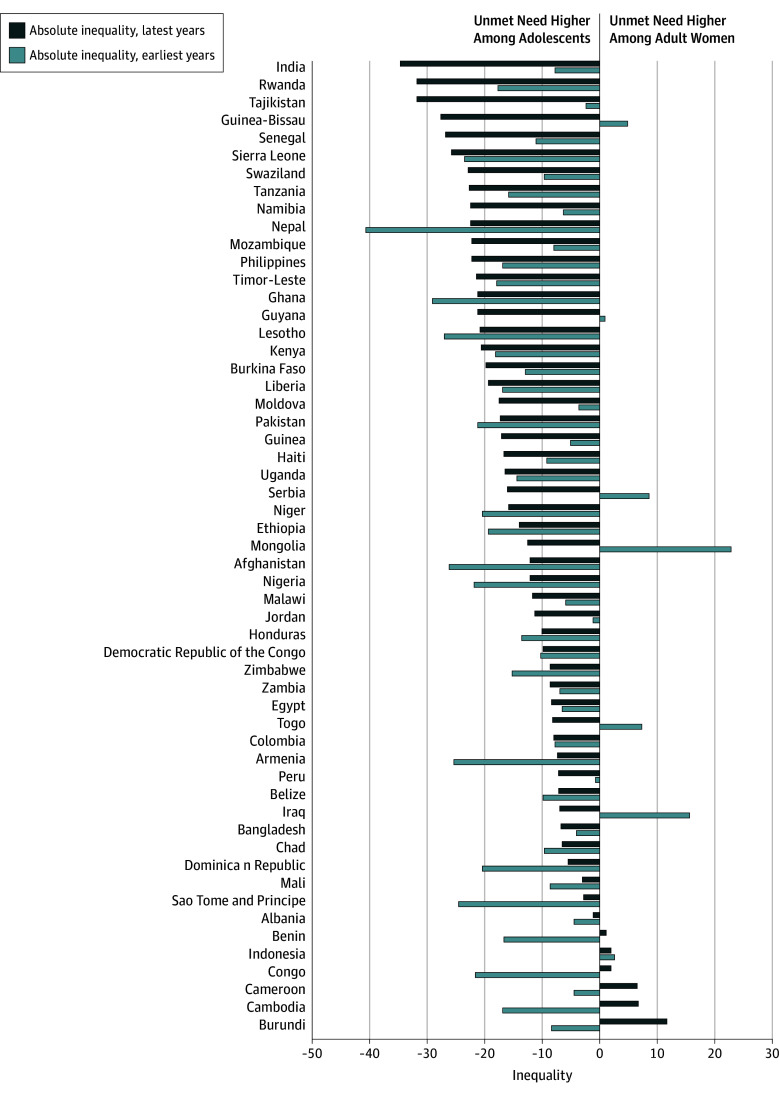
Absolute Inequality in Percentage Points in Unmet Need for Family Planning Between Adult Women and Adolescents, Latest Data vs Earliest Data

Over time, absolute inequalities between adolescent girls and adult women in unmet need for family planning increased in 20 countries, 13 of which were FP2020 countries. In many countries, greater inequality was driven by increased unmet needs in adolescents. In India, for example, both age groups had increased percentage of unmet need for family planning over time, from 16.2% (95% CI, 15.9%-16.6%) in 2006 to 29.8% (95% CI, 29.6%-30.1%) in 2015 for adult women and from 23.9% (95% CI, 23.0%-24.9%) to 64.5% (95% CI, 63.3%-65.7%) among adolescents. The inequality increased from 7.7 PPs (95% CI, 7.2-8.2 PPs) in 2006 to 34.7 PPs (95% CI, 34.2-35.1 PPs) in 2015. In 17 of the 55 countries (including 15 FP2020 countries), the gap in unmet need for family planning decreased, primarily owing to a larger improvement among adolescents. Cambodia, Congo, Sao Tome, and Principe, which were all FP2020 countries, were among the most successful in closing the gap between adolescents and adult women (Figure 4; eTable 13 in the Supplement).

## Discussion

Using nationally representative surveys across 103 countries, we found a low uptake of contraception for both adolescents and adult women: only 41% of women aged 15 to 34 years used modern contraception, and 38% had their family planning need unsatisfied. Inequality between the 2 age groups was striking for both contraceptive use and unmet need, with the gap in many countries becoming larger over time. Worsened inequality could occur in any region or income group and occurred in both FP2020 and non-FP2020 countries, suggesting that the action to close the gap between adolescents and adult women should be universal.

Family planning among young women is essential in avoiding unintended pregnancy and unsafe abortion, ultimately improving maternal and child health.^5,9,10^ Although adolescents were prioritized in FP2020s expansion of access to family planning, adolescent girls had less progress than older women in many countries.^5,34,35,36^ Many of the barriers for adolescents to family planning are shared with older women, but their impact was disproportionally large in the younger group. Unaffordability of services disproportionally affects adolescents, owing to their limited financial resources and weak power over financial decisions.^36^ Adolescents also commonly have poor contraceptive knowledge, perpetuating myths and misinformation,^36,37,38^ including misconceptions about the short- and long-term adverse effects of using contraceptives on health and their future ability to conceive a healthy baby.^35^ Among nonmarried adolescents, the uptake of contraception might be further constrained by stigma around nonmarital sexual activity, particularly in low- and middle-income settings where the provision of family planning services lacks privacy and confidentiality.^39^ Many girls are subject to judgmental attitudes, disrespect, and suboptimal service from health workers, effectively limiting access to contraception.^40^ Among married adolescents, there is often an additional societal pressure to conceive a first child soon after marriage to demonstrate fertility.^37^ Moreover, young women in many cultures are restricted in their mobility after marriage^41^ and excluded from social networks,^42^ which might reduce their autonomy in family planning decision-making.^43^

Some countries appeared to be more effective in bringing family planning to adolescents, with Nepal a remarkable example. Adolescent-friendly health services were launched in Nepal in 2008 with further scaling up of FP2020 from 2015. The aim of this program was to provide high-quality, confidential, nondiscriminating reproductive health services for adolescents in well-equipped facilities, as well as providing information, education, and communication materials and other educational services.^44,45^ Our study found that inequality between adolescents and adult women decreased in Nepal between 2006 and 2016 for both modern contraceptive use and unmet need for family planning.

### Limitations

The study has some limitations. First, although it is, to our knowledge, the most comprehensive report to date, we were only able to include 103 LMICs, and only 43 had data available in all 3 rounds. Moreover, in some DHS and MICS surveys, information on modern contraceptive use and family planning need was not collected. Our results, therefore, were not representative at the global, regional, or income group levels. With more data available in the future, aggregate-level analyses could be strengthened. Second, both indicators were self-reported and subject to poor recall of information and misreporting. Third, our study only focused on contraceptive use, not whether the contraception was used correctly, which may also differ across age groups. Fourth, the classification of wealth quintiles is country specific and time sensitive. The poorest quintile in an upper-middle-income country could be higher than the richest quintile in a lower-income country.^46,47^ Future study on this topic will consider using absolute wealth quintiles.^48^

## Conclusions

Given that adolescents were not a priority group during the Millennium Development Goals, a gap in meeting family planning needs is perhaps understandable. However, since the launch of FP2020 in 2012, more efforts have been made to improve adolescents’ access to contraceptives and address the fundamental right of young people to make their own childbearing decisions.^11^ A growing gap between adolescents and older women, particularly in FP2020 countries, suggests that the measures adopted to date may not have been effective in many countries. There is a now a pressing need for further work to better understand the barriers to uptake of family planning in adolescents and to evaluate adolescent-targeted interventions in promoting contraceptive use. With the adoption of more age-appropriate strategies, we would hope that the gap between adolescent girls and adult women could be closed over the next decade.

## Notes

Supplement. **eTable 1.** Data Sources of the Countries Involved in This Study, 261 Surveys **eTable 2.** Definition and Explanation of Inequality Measurements **eTable 3.** Estimates of Overall Level of Contraception Use and Unmet Need, Using the Most Recent Available Survey Data Since 2010 **eFigure 1.** Wealth- and Residence-Based Inequalities **eTable 4.** Prevalence of Modern Contraceptive Use Among Adolescents 15-19 Years Old and Adult Women 20-34 Years Old in Poorest Wealth Quintile (Q1), Richest Wealth Quintile (Q5), and Slope Index of Inequality (SII), Using the Most Recent Years of Data Available **eTable 5.** Prevalence of Unmet Need for Family Planning Among Adolescents 15-19 Years Old and Adult Women 20-34 Years Old in Poorest Wealth Quintile (Q1), Richest Wealth Quintile (Q5), and Slope Index of Inequality (SII), Using the Most Recent Years of Data Available **eTable 6.** Prevalence of Modern Contraceptive Use Among Adolescents 15-19 Years Old and Adult Women 20-34 Years Old in Urban Area, Rural Area, and Absolute Inequality Between Urban and Rural Areas (Diff), Using the Most Recent Years of Data Available **eTable 7.** Prevalence of Unmet Need for Family Planning Among Adolescents 15-19 Years Old and Adult Women 20-34 Years Old in Urban Area, Rural Area, and Absolute Inequality Between Urban and Rural Areas (Diff), Using the Most Recent Years of Data Available **eTable 8.** Estimates of Contraception Use and Unmet Need, Using Data From 43 Countries With Surveys Available in All Three Rounds **eTable 9.** Absolute Inequality in Contraception Use and Unmet Need by FP2020 Group, Using Data From 43 Countries With Surveys Available in All Three Rounds **eTable 10.** Modern Contraceptive Use Among Adolescents 15-19 Years Old and Adult Women 20-34 Years Old, and Absolute Inequality **eTable 11.** Unmet Need for Family Planning Among Adolescents 15-19 Years Old and Adult Women 20-34 Years Old, and Absolute Inequality **eFigure 2.** Relative Inequality in Contraception Use and Unmet Need Between Adolescents 15-19 Years Old and Adult Women 20-34 Years Old, the Latest Years **eTable 12.** Changes in Absolute Inequality of Modern Contraceptive Use (MC) Between Adult Women and Adolescents at Country-Level, the Latest Year vs. the Earliest Year **eTable 13.** Changes in Absolute Inequality of Unmet Need for Family Planning (unmet_FP) Between Adult Women and Adolescents at Country-Level, the Latest Year vs. the Earliest Year
