# Errors in Abstract, Results, and Table

**DOI:** 10.1001/jamanetworkopen.2020.2596

**Published:** 2020-03-12

In the Original Investigation titled “Contraceptive Use in Adolescent Girls and Adult Women in Low- and Middle-Income Countries,”^1^ published February 19, 2020, there were errors in the text of the Abstract and Results section and in Table 1 for the 95% confidence intervals for unmet need for family planning among women aged 20 to 34 years and absolute inequality between adolescent girls and adult women, which appeared as 36.4% (95% CI, 34.9% to 35.8%) and −14.4 percentage points (95% CI, −15.8 to −15.0 percentage points), respectively. These should have appeared as 36.4% (95% CI, 35.9% to 36.8%) and −14.4 percentage points (95% CI, −14.8 to −14.0 percentage points), respectively. This article has been corrected.^1^
