# Supplementary material for: Contraceptive Use in Adolescent Girls and Adult Women in Low- and Middle-Income Countries
Source: JAMA Netw Open. 2020 Feb 19;3(2):e1921437. doi: 10.1001/jamanetworkopen.2019.21437 (PMC12634135; doi:10.1001/jamanetworkopen.2019.21437)
Supplement: Supplement. — eTable 1. Data Sources of the Countries Involved in This Study, 261 Surveys eTable 2. Definition and Explanation of Inequality Measurements eTable 3. Estimates of Overall Level of Contraception Use and Unmet Need, Using the Most Recent Available Survey Data Since 2010 eFigure 1. Wealth- and Residence-Based Inequalities eTable 4. Prevalence of Modern Contraceptive Use Among Adolescents 15-19 Years Old and Adult Women 20-34 Years Old in Poorest Wealth Quintile (Q1), Richest Wealth Quintile (Q5), and Slope Index of Inequality (SII), Using the Most Recent Years of Data Available eTable 5. Prevalence of Unmet Need for Family Planning Among Adolescents 15-19 Years Old and Adult Women 20-34 Years Old in Poorest Wealth Quintile (Q1), Richest Wealth Quintile (Q5), and Slope Index of Inequality (SII), Using the Most Recent Years of Data Available eTable 6. Prevalence of Modern Contraceptive Use Among Adolescents 15-19 Years Old and Adult Women 20-34 Years Old in Urban Area, Rural Area, and Absolute Inequality Between Urban and Rural Areas (Diff), Using the Most Recent Years of Data Available eTable 7. Prevalence of Unmet Need for Family Planning Among Adolescents 15-19 Years Old and Adult Women 20-34 Years Old in Urban Area, Rural Area, and Absolute Inequality Between Urban and Rural Areas (Diff), Using the Most Recent Years of Data Available eTable 8. Estimates of Contraception Use and Unmet Need, Using Data From 43 Countries With Surveys Available in All Three Rounds eTable 9. Absolute Inequality in Contraception Use and Unmet Need by FP2020 Group, Using Data From 43 Countries With Surveys Available in All Three Rounds eTable 10. Modern Contraceptive Use Among Adolescents 15-19 Years Old and Adult Women 20-34 Years Old, and Absolute Inequality eTable 11. Unmet Need for Family Planning Among Adolescents 15-19 Years Old and Adult Women 20-34 Years Old, and Absolute Inequality eFigure 2. Relative Inequality in Contraception Use and Unmet Need Between Adolescents 15-19 Years Ol [file jamanetwopen-e1921437-s001.pdf]

Supplementary Online Content

Li Z, Patton G, Sabet F, Zhou Z, Subramanian SV, Lu C. Contraceptive use in adolescent girls and adult women in low- and middle-income countries. *JAMA Netw Open*. 2020;3(2):e1921437. doi:10.1001/jamanetworkopen.2019.21437

eTable 1. Data Sources of the Countries Involved in This Study, 261 Surveys
eTable 2. Definition and Explanation of Inequality Measurements
eTable 3. Estimates of Overall Level of Contraception Use and Unmet Need, Using the Most Recent Available Survey Data Since 2010
eFigure 1. Wealth- and Residence-Based Inequalities
eTable 4. Prevalence of Modern Contraceptive Use Among Adolescents 15-19 Years Old and Adult Women 20-34 Years Old in Poorest Wealth Quintile (Q1), Richest Wealth Quintile (Q5), and Slope Index of Inequality (SII), Using the Most Recent Years of Data Available
eTable 5. Prevalence of Unmet Need for Family Planning Among Adolescents 15-19 Years Old and Adult Women 20-34 Years Old in Poorest Wealth Quintile (Q1), Richest Wealth Quintile (Q5), and Slope Index of Inequality (SII), Using the Most Recent Years of Data Available
eTable 6. Prevalence of Modern Contraceptive Use Among Adolescents 15-19 Years Old and Adult Women 20-34 Years Old in Urban Area, Rural Area, and Absolute Inequality Between Urban and Rural Areas (Diff), Using the Most Recent Years of Data Available
eTable 7. Prevalence of Unmet Need for Family Planning Among Adolescents 15-19 Years Old and Adult Women 20-34 Years Old in Urban Area, Rural Area, and Absolute Inequality Between Urban and Rural Areas (Diff), Using the Most Recent Years of Data Available
eTable 8. Estimates of Contraception Use and Unmet Need, Using Data From 43 Countries With Surveys Available in All Three Rounds
eTable 9. Absolute Inequality in Contraception Use and Unmet Need by FP2020 Group, Using Data From 43 Countries With Surveys Available in All Three Rounds
eTable 10. Modern Contraceptive Use Among Adolescents 15-19 Years Old and Adult Women 20-34 Years Old, and Absolute Inequality
eTable 11. Unmet Need for Family Planning Among Adolescents 15-19 Years Old and Adult Women 20-34 Years Old, and Absolute Inequality
eFigure 2. Relative Inequality in Contraception Use and Unmet Need Between Adolescents 15-19 Years Old and Adult Women 20-34 Years Old, the Latest Years
eTable 12. Changes in Absolute Inequality of Modern Contraceptive Use (MC) Between Adult Women and Adolescents at Country-Level, the Latest Year vs. the Earliest Year
eTable 13. Changes in Absolute Inequality of Unmet Need for Family Planning (unmet_FP) Between Adult Women and Adolescents at Country-Level, the Latest Year vs. the Earliest Year

© 2020 Li Z et al. *JAMA Network Open.*

This supplementary material has been provided by the authors to give readers additional information about their work.

© 2020 Li Z et al. *JAMA Network Open.*

eTable 1. Data sources of the countries involved in this study, 261 surveys

Country	Round1 (2000-2006)	Round2 (2007-2012)	Round3 (2013-2017)
Afghanistan		MICS 2010	DHS 2015
Albania	MICS 2005	DHS 2009	DHS 2017
Algeria	MICS 2006	MICS 2012	
Angola			DHS 2015
Argentina		MICS 2011	
Armenia	DHS 2005	DHS 2010	MICS 2016
Azerbaijan	DHS 2006		
Bangladesh	DHS 2004	DHS 2007, DHS 2011	MICS 2013, DHS 2014
Belarus	MICS 2005	MICS 2012	
Belize	MICS 2006	DHS 2011	MICS 2016
Benin	DHS 2006	DHS 2012	MICS 2014
Bhutan		MICS 2010	
Bolivia	MICS 2000, DHS 2003	DHS 2008	
Bosnia and Herzegovina	MICS 2006	MICS 2011	
Burkina Faso	DHS 2003, MICS 2006	DHS 2010	
Burundi	MICS 2005	DHS 2010	DHS 2016
Cambodia	DHS 2005	DHS 2010	DHS 2014
Cameroon	DHS 2004, MICS 2006	DHS 2011	MICS 2014
Central African Republic	MICS 2000, MICS 2006	DHS 2010	
Chad	MICS 2000, DHS 2004	MICS 2010	DHS 2015
Colombia	DHS 2005	DHS 2010	DHS 2015
Comoros		DHS 2012	
Congo, Dem. Rep.		DHS 2007, MICS 2010	DHS 2013
Congo	DHS 2005	DHS 2011	MICS 2015
Costa Rica		MICS 2011	
Côte d'Ivoire	MICS 2006	DHS 2012	MICS 2016
Cuba	MICS 2006	MICS 2010	MICS 2014
Djibouti	MICS 2006		
Dominican Republic	MICS 2000	DHS 2007	DHS 2013, MICS 2014
Egypt	DHS 2005	DHS 2008	DHS 2014
El Salvador			MICS 2014
Ethiopia	DHS 2005	DHS 2011	DHS 2016
Gabon			DHS 2012
Gambia		MICS 2010	DHS 2013
Georgia	MICS 2005		
Ghana	DHS 2003, MICS 2006	DHS 2008, MICS 2011	DHS 2014
Guatemala			DHS 2015
Guinea	DHS 2005	DHS 2012	MICS 2016
Guinea-Bissau	MICS 2000, MICS 2006	MICS 2010	MICS 2014

© 2020 Li Z et al. *JAMA Network Open.*

eTable 1. Data sources of the countries involved in this study, 261 surveys (continued)

Country	Round1 (2000-2006)	Round2 (2007-2012)	Round3 (2013-2017)
Guyana	MICS 2006	DHS 2009	MICS 2014
Haiti	DHS 2006	DHS 2012	DHS 2017
Honduras	DHS 2006	DHS 2012	
India	DHS 2006		DHS 2015
Indonesia	DHS 2002	DHS 2007	DHS 2017
Iraq	MICS 2006	MICS 2011	
Jordan		DHS 2007, DHS 2012	
Kazakhstan	MICS 2006	MICS 2010	MICS 2015
Kenya	DHS 2003	DHS 2009	DHS 2014
Kosovo			MICS 2014
Kyrgyzstan	MICS 2005	DHS 2012	MICS 2014
Lao PDR		MICS 2011	MICS 2017
Lebanon (Palestinians)	MICS 2005	MICS 2011	
Lesotho	DHS 2004	DHS 2009	DHS 2014
Liberia		DHS 2007	DHS 2013
Madagascar	DHS 2004	DHS 2009	
Malawi	DHS 2004, MICS 2006	DHS 2010	MICS 2013, DHS 2015
Maldives		DHS 2009	
Mali	DHS 2006	MICS 2009, DHS 2012	MICS 2015
Mauritania		MICS 2007, MICS 2011	MICS 2015
Mexico			MICS 2015
Moldova	DHS 2005	MICS 2012	
Mongolia	MICS 2005	MICS 2010	MICS 2013
Montenegro	MICS 2005		MICS 2013
Morocco	DHS 2003		
Mozambique	DHS 2003	MICS 2008, DHS 2011	
Myanmar		MICS 2009	DHS 2016
Namibia		DHS 2007	DHS 2013
Nepal	DHS 2006	DHS 2011	MICS 2014, DHS 2016
Niger	DHS 2006	DHS 2012	
Nigeria	DHS 2003	MICS 2007, DHS 2008, MICS 2011	DHS 2013, MICS 2017
Pakistan		DHS 2006, DHS 2012	
Panama			MICS 2013
Paraguay			MICS 2016
Peru	DHS 2006	DHS 2008, DHS 2009, DHS 2010, DHS 2011, DHS 2012	

© 2020 Li Z et al. *JAMA Network Open.*

eTable 1. Data sources of the countries involved in this study, 261 surveys (continued)

Country	Round1 (2000-2006)	Round2 (2007-2012)	Round3 (2013-2017)
Philippines	DHS 2003	DHS 2008	DHS 2013, DHS 2017
Rwanda	DHS 2005	DHS 2010	DHS 2015
Sao Tome and Principe		DHS 2008	MICS 2014
Senegal	DHS 2005	DHS 2011	DHS 2013, DHS 2014, DHS 2015, DHS 2016, DHS 2017
Serbia	MICS 2005	MICS 2010	MICS 2014
Sierra Leone	DHS 2005	DHS 2008, MICS 2010	DHS 2013, MICS 2017
Somalia	MICS 2006		
South Sudan		MICS 2010	
Sudan		MICS 2010	MICS 2014
Suriname	MICS 2006	MICS 2010	
Swaziland		MICS 2010	MICS 2014
Syria		MICS 2006	
Tajikistan	MICS 2005	DHS 2012	DHS 2017
Tanzania	DHS 2004	DHS 2010	DHS 2015
Thailand	MICS 2005, MICS 2006	MICS 2012	MICS 2015
The former Yugoslav Republic of Macedonia	MICS 2005	MICS 2011	
Timor-Leste		DHS 2009	DHS 2016
Togo	MICS 2006	MICS 2010	DHS 2014
Trinidad and Tobago	MICS 2000 MICS 2006	MICS 2011	
Tunisia		MICS 2012	
Turkey	DHS 2003		
Turkmenistan	MICS 2006		MICS 2016
Uganda	DHS 2006	DHS 2011	DHS 2016
Ukraine	MICS 2005	DHS 2007	
Uzbekistan	MICS 2006		
Vanuatu		MICS 2007	
Vietnam	MICS 2006	MICS 2011	MICS 2013
West Bank and Gaza Strip		MICS 2010	MICS 2014
Yemen		MICS 2006	DHS 2013
Zambia		DHS 2007	DHS 2013
Zimbabwe	DHS 2005	MICS 2009, DHS 2010	MICS 2014, DHS 2015

© 2020 Li Z et al. *JAMA Network Open.*

eTable 2. Definition and explanation of inequality measurements

Measurement	Definition	Explanation
Inequalities between adolescent girls and adult women		
Absolute inequality (percentage points)	The difference in use between adult women and adolescents	Modern contraceptive use: a value larger than zero indicates that adult women are in a better situation comparing to adolescents
		Unmet need for family planning: a value larger than zero indicates that adult women are in an inferior situation comparing to adolescents
Relative inequality	The ratio in use between adult women and adolescents	Modern contraceptive use: a value larger than 1 means that adult women are in better situation comparing to adolescents
		Unmet need for family planning: a value larger than 1 means adult women are in inferior situation comparing to adolescents
Wealth-based inequalities		
Slope index of inequality	The absolute difference in use between the two extremes of the wealth distribution	Modern contraceptive use: a positive value represents a pro-rich distribution.
		Unmet need for family planning: a positive value represents a pro-poor distribution.
Residence-based inequalities		
Absolute inequality (percentage points)	The difference in use between urban and rural areas	Modern contraceptive use: A positive value indicators urban areas are in a better situation comparing to rural areas
		Unmet need for family planning: A positive value indicators rural areas are in a superior situation comparing to urban areas

© 2020 Li Z et al. *JAMA Network Open.*

eTable 3. Estimates of overall level of contraception use and unmet need, using the most recent available survey data since 2010

Category	Modern contraceptive use (15-34, %)	Unmet need for family planning (15-34, %)
All available countries	40.5(40.4, 40.6)	38.1(36.5, 39.6)
WHO regions		
Africa	18.0(17.4, 18.6)	60.2(59.0, 61.4)
Americas	57.5(56.9, 58.1)	22.2(20.7, 23.7)
Eastern Mediterranean	35.3(33.0, 37.6)	39.9(37.0, 42.8)
Europe	42.4(40.7, 44.1)	26.3(23.6, 29.0)
South-East Asia	52.0(51.0, 52.9)	23.3(22.5, 24.0)
Western Pacific	46.2(44.1, 48.2)	30.5(29.0, 32.1)
Country-income class		
Low	35.7(34.6, 36.9)	40.8(39.4, 42.3)
Lower-middle	40.2(38.9, 41.4)	35.4(33.9, 36.9)
Upper-middle	58.2(56.5, 60.0)	22.6(20.7, 24.5)
SSA category[1]		
Non-SSA	49.3(48.6, 50.0)	26.8(26.2, 27.5)
SSA	17.5(16.7, 18.3)	60.7(59.6, 61.9)
FP2020 group[2]		
Non-FP2020 countries	52.9(51.5, 54.3)	22.9(21.2, 24.6)
FP2020 countries	36.2(35.4, 37.0)	40.9(38.9, 42.9)

Note:

1. "SSA" represents "Sub-Saharan African countries".
2. "FP2020" represents "Family Planning 2020". See https://www.familyplanning2020.org/countries for the list of FP2020 prioritized countries. (Accessed on Dec 13th, 2019).

© 2020 Li Z et al. *JAMA Network Open.*

eFigure 1. Wealth- and Residence-Based Inequalities

eFigure 1A. Wealth-based inequalities in both the adolescent girl (15-19 years old) and adult woman (20-34 years old) groups, the latest surveys[1]

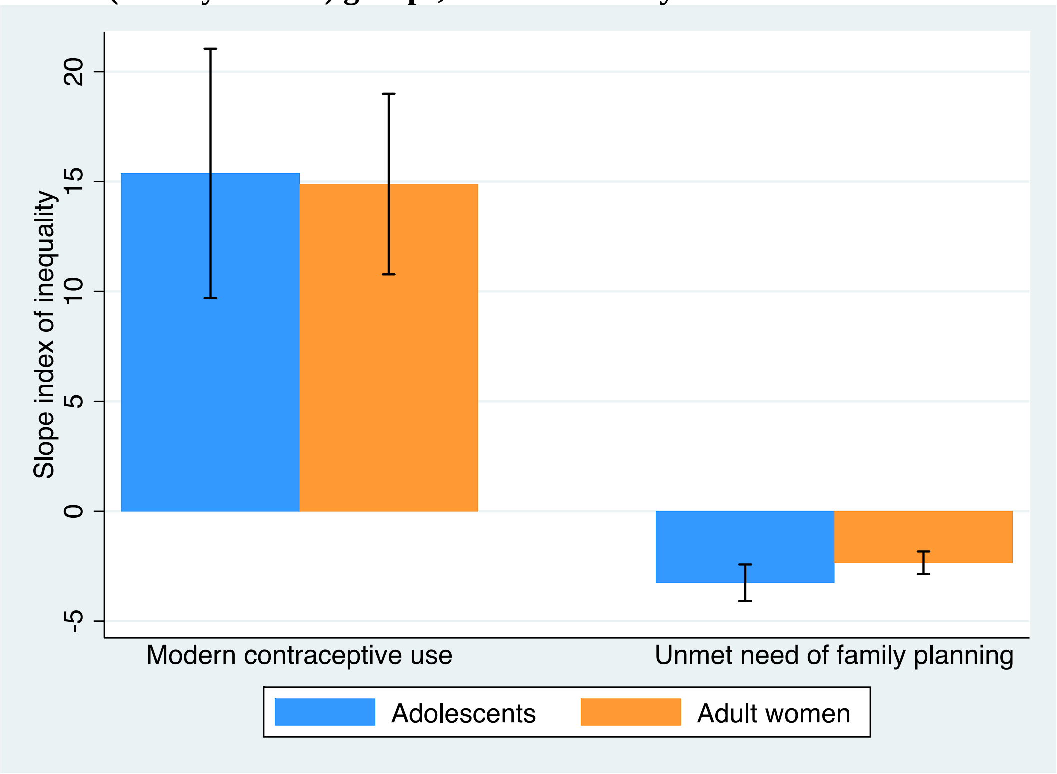

Note:

1. Caps on each bar show 95% CIs.
2. We multiplied the value by 100 to better show the inequalities.

eFigure 1B. Residence-based inequalities in both the adolescent girl (15-19 years old) and adult woman (20-34 years old) groups, the latest surveys[1]

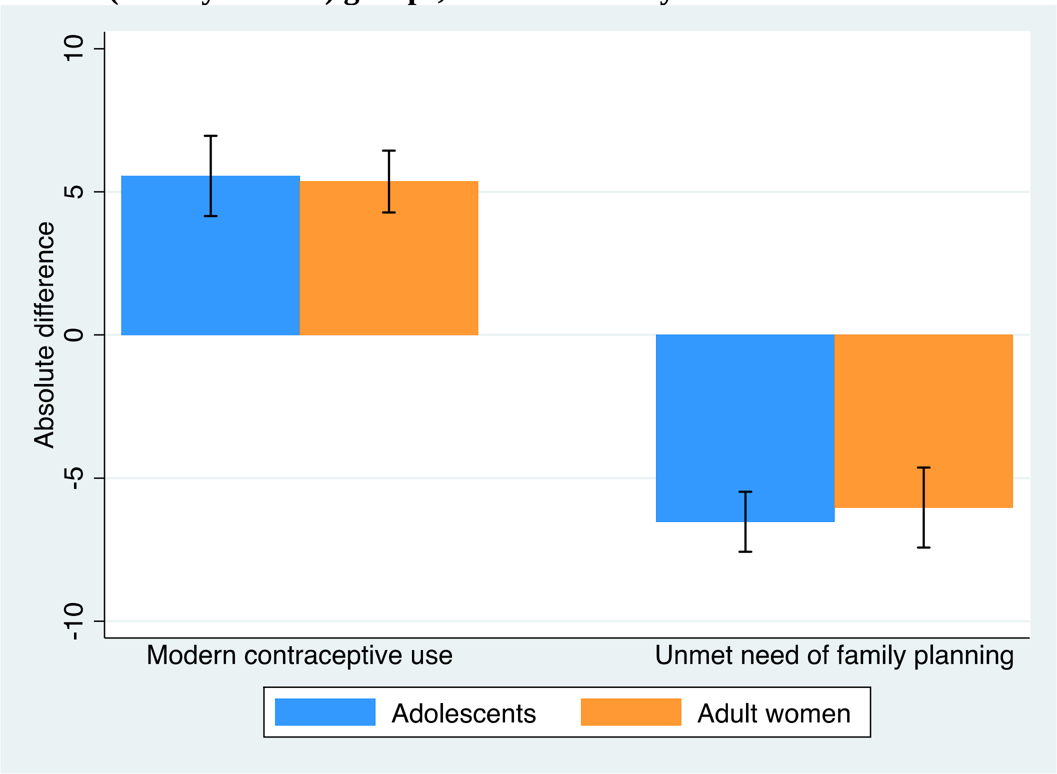

© 2020 Li Z et al. *JAMA Network Open.*

eTable 4. Prevalence of modern contraceptive use among adolescents 15-19 years old and adult women 20-34 years old in poorest wealth quintile (Q1), richest wealth quintile (Q5), and slope index of inequality (SII), using the most recent years of data available

		15-19			20-34		
Country	Year	Q1 (%)	Q5 (%)	SII	Q1 (%)	Q5 (%)	SII
Afghanistan	2015	2.5(0.9, 4.1)	9.7(6.1, 13.2)	19.4(11.9, 26.8)	10.9(9.8, 12.0)	25.9(24.2, 27.6)	43.7(39.4, 48.0)
Albania	2017	1.6(0.0, 4.8)	25.0(0.0, 63.7)	14.9(-10.1, 39.8)	3.1(1.9, 4.2)	5.9(3.5, 8.3)	1.5(-3.8, 6.8)
Algeria	2012	22.9(12.8, 32.9)	34.8(13.7, 55.8)	0.2(-0.2, 0.6)	49.6(47.4, 51.8)	49.0(46.3, 51.6)	-0.1(-0.1, 0.0)
Angola	2015	0.8(0.0, 1.8)	36.8(27.5, 46.1)	44.5(37.0, 52.0)	0.8(0.2, 1.4)	35.4(32.1, 38.7)	60.6(56.9, 64.3)
Bangladesh	2014	46.3(41.3, 51.4)	49.0(43.3, 54.7)	9.7(-4.0, 23.5)	63.3(61.0, 65.5)	57.4(55.3, 59.6)	-7.0(-12.3, -1.6)
Belarus	2012	33.3(2.0, 64.6)	50.0(0.0, 100.0)	-0.4(-1.2, 0.4)	48.1(42.5, 53.6)	58.2(54.6, 61.7)	0.1(0.0, 0.2)
Belize	2016	27.3(13.6, 41.0)	50.0(0.0, 100.0)	0.2(-0.2, 0.7)	38.1(32.9, 43.2)	55.5(49.3, 61.6)	0.3(0.1, 0.4)
Benin	2014	3.8(0.1, 7.5)	15.2(6.3, 24.0)	0.3(0.1, 0.5)	5.9(4.5, 7.3)	18.3(16.4, 20.1)	0.3(0.3, 0.4)
Bhutan	2010	46.2(35.9, 56.6)	27.8(4.9, 50.7)	-0.1(-0.4, 0.2)	66.6(63.7, 69.5)	60.6(57.7, 63.4)	-0.1(-0.2, -0.1)
Burkina Faso	2010	3.8(1.0, 6.5)	35.1(28.8, 41.4)	95.3(82.4, 108.2)	7.5(6.1, 9.0)	37.8(35.5, 40.0)	77.8(72.2, 83.4)
Burundi	2016	23.3(12.3, 34.4)	36.7(22.7, 50.7)	49.7(9.8, 89.6)	22.5(20.1, 25.0)	29.7(27.2, 32.1)	11.2(3.7, 18.6)
Cambodia	2014	23.9(15.7, 32.0)	19.5(10.4, 28.5)	-4.6(-26.9, 17.7)	47.4(44.6, 50.2)	36.5(34.3, 38.7)	-19.8(-26.0, -13.6)
Cameroon	2014	1.1(0.0, 3.4)	12.9(0.4, 25.4)	0.4(0.2, 0.6)	2.6(1.3, 4.0)	30.5(27.1, 33.9)	0.5(0.5, 0.6)
Central African Republic	2010	1.9(0.4, 3.5)	16.3(10.4, 22.3)	0.5(0.4, 0.5)	1.2(0.5, 1.8)	21.8(18.7, 24.8)	0.6(0.5, 0.6)
Chad	2015	1.6(0.2, 2.9)	6.9(4.0, 9.7)	24.0(18.1, 29.9)	2.0(1.3, 2.7)	11.7(10.0, 13.4)	30.4(27.0, 33.8)
Colombia	2015	46.5(42.6, 50.5)	75.8(68.2, 83.4)	46.1(36.6, 55.5)	59.4(57.7, 61.2)	75.2(72.4, 77.9)	21.5(17.6, 25.4)
Comoros	2012	5.7(0.0, 12.1)	14.6(3.3, 25.9)	5.6(-21.1, 32.3)	10.3(7.0, 13.6)	17.3(13.5, 21.0)	13.1(3.8, 22.3)
Congo	2015	12.8(8.1, 17.6)	36.4(2.5, 70.3)	0.2(0.0, 0.3)	13.7(11.9, 15.6)	22.6(18.2, 27.1)	0.1(0.1, 0.2)
Costa Rica	2011	65.3(54.3, 76.4)	71.4(26.3, 100.0)	0.2(-0.3, 0.6)	71.1(67.1, 75.1)	75.3(68.5, 82.1)	0.2(0.1, 0.4)
Côte d'Ivoire	2016	1.8(0.0, 3.8)	16.7(0.0, 35.7)	0.2(0.0, 0.4)	6.9(5.6, 8.3)	23.5(19.7, 27.3)	0.4(0.3, 0.4)
Democratic Republic of the Congo	2013	4.6(2.5, 6.7)	15.4(9.4, 21.4)	27.1(17.8, 36.4)	2.8(2.1, 3.5)	18.0(15.8, 20.1)	25.6(22.6, 28.7)
Dominican Republic	2014	48.7(44.6, 52.8)	53.7(39.3, 68.1)	0.2(0.0, 0.4)	66.3(64.5, 68.0)	65.4(62.9, 67.9)	-0.0(-0.1, 0.0)

© 2020 Li Z et al. *JAMA Network Open.*

eTable 4. Prevalence of modern contraceptive use among adolescents 15-19 years old and adult women 20-34 years old in poorest wealth quintile (Q1), richest wealth quintile (Q5), and slope index of inequality (SII), using the most recent years of data available (continued)

		15-19			20-34		
**Country**	**Year**	**Q1 (%)**	**Q5 (%)**	**SII**	**Q1 (%)**	**Q5 (%)**	**SII**
Egypt	2014	14.6(9.1, 20.2)	15.9(6.6, 25.2)	15.3(-17.2, 47.8)	33.6(31.5, 35.7)	53.9(52.0, 55.8)	60.6(50.5, 70.7)
El Salvador	2014	59.4(53.4, 65.4)	57.2(45.9, 68.4)	0.1(-0.1, 0.2)	58.9(55.9, 61.8)	74.3(70.7, 78.0)	0.2(0.1, 0.3)
Ethiopia	2016	8.0(4.8, 11.2)	39.6(31.5, 47.7)	72.0(40.2, 103.8)	11.1(9.6, 12.6)	51.1(48.8, 53.4)	67.6(59.0, 76.2)
Gabon	2012	1.0(0.0, 6.0)	30.2(17.0, 43.4)	48.1(30.6, 65.6)	16.4(14.2, 18.7)	31.9(26.9, 36.8)	32.1(23.8, 40.4)
Gambia	2013	1.6(0.0, 3.5)	6.1(0.0, 13.1)	8.3(0.0, 16.6)	4.5(3.2, 5.9)	17.2(14.4, 20.0)	25.4(20.6, 30.3)
Ghana	2014	13.8(5.1, 22.5)	19.6(0.0, 41.5)	-17.1(-56.8, 22.6)	22.2(19.3, 25.0)	21.4(17.9, 24.9)	-4.5(-14.4, 5.5)
Guatemala	2015	23.0(18.4, 27.7)	49.6(40.9, 58.3)	53.2(39.5, 66.8)	34.4(32.1, 36.7)	59.8(57.3, 62.2)	47.5(42.3, 52.6)
Guinea	2016	100.0(100.0, 100.0)	9.8(3.2, 16.3)	0.2(0.1, 0.2)	1.6(0.8, 2.4)	10.4(8.2, 12.7)	0.1(0.1, 0.2)
Guinea-Bissau	2014	1.1(0.0, 3.4)	6.2(0.0, 19.6)	0.1(-0.1, 0.3)	6.0(4.5, 7.5)	24.6(20.0, 29.3)	0.5(0.4, 0.6)
Guyana	2014	14.5(5.5, 23.5)	25.0(0.0, 53.7)	0.3(-0.1, 0.6)	37.8(33.6, 42.1)	44.4(38.6, 50.2)	0.1(-0.0, 0.2)
Haiti	2017	31.4(24.6, 38.2)	36.9(26.6, 47.3)	9.0(-9.3, 27.4)	33.2(30.2, 36.3)	31.8(28.7, 34.9)	-0.3(-6.4, 5.8)
Honduras	2012	43.9(39.1, 48.6)	57.6(48.6, 66.7)	19.4(6.9, 31.9)	57.2(55.0, 59.5)	65.7(62.9, 68.5)	8.1(3.2, 13.0)
India	2015	40.1(39.4, 40.7)	46.6(44.6, 48.6)	8.1(5.2, 11.0)	44.2(43.8, 44.6)	55.9(55.5, 56.4)	19.8(18.8, 20.8)
Indonesia	2017	45.4(40.6, 50.1)	49.8(35.4, 64.3)	17.5(-4.0, 39.0)	57.5(56.0, 59.0)	51.8(49.8, 53.8)	-6.6(-11.6, -1.5)
Iraq	2011	9.5(7.3, 11.8)	13.7(9.4, 17.9)	0.1(-0.1, 0.2)	27.2(26.1, 28.4)	31.3(29.3, 33.3)	0.2(0.1, 0.2)
Jordan	2012	13.9(5.7, 22.1)	25.0(0.0, 63.7)	95.4(39.5, 151.4)	36.1(33.4, 38.9)	38.4(33.9, 43.0)	-4.7(-18.0, 8.5)
Kazakhstan	2015	22.2(0.9, 43.5)	20.0(0.0, 50.2)	0.4(-0.1, 0.9)	53.7(49.9, 57.5)	49.9(46.7, 53.2)	-0.0(-0.1, 0.0)
Kenya	2014	20.7(16.3, 25.0)	46.6(34.9, 58.3)	84.1(60.6, 107.6)	24.2(22.7, 25.7)	56.7(54.7, 58.8)	42.6(37.1, 48.1)
Kosovo	2014	100.0(100.0, 100.0)	100.0(100.0, 100.0)	0.4(-0.4, 1.3)	10.5(6.8, 14.3)	13.9(9.8, 18.0)	0.1(-0.1, 0.2)
Kyrgyzstan	2014	6.1(0.0, 14.7)	100.0(100.0, 100.0)	-0.0(-0.3, 0.3)	37.7(34.0, 41.5)	37.9(33.4, 42.3)	0.1(0.0, 0.2)
Lao People's Democratic Republic	2011	12.6(8.9, 16.4)	31.6(22.1, 41.1)	0.4(0.3, 0.6)	44.1(41.9, 46.2)	45.7(43.2, 48.3)	-0.0(-0.1, 0.0)
Lebanon	2011	23.8(3.9, 43.7)	36.4(2.5, 70.3)	0.4(-0.3, 1.0)	48.5(41.5, 55.5)	56.2(50.3, 62.2)	0.2(0.0, 0.4)
Lesotho	2014	30.9(20.6, 41.1)	60.0(42.9, 77.1)	42.8(15.0, 70.5)	57.2(52.8, 61.7)	69.6(65.6, 73.7)	19.1(9.6, 28.6)

© 2020 Li Z et al. *JAMA Network Open.*

eTable 4. Prevalence of modern contraceptive use among adolescents 15-19 years old and adult women 20-34 years old in poorest wealth quintile (Q1), richest wealth quintile (Q5), and slope index of inequality (SII), using the most recent years of data available (continued)

		15-19			20-34		
**Country**	**Year**	**Q1 (%)**	**Q5 (%)**	**SII**	**Q1 (%)**	**Q5 (%)**	**SII**
Liberia	2013	12.6(8.3, 16.9)	27.2(18.9, 35.5)	33.2(17.4, 48.9)	18.3(16.0, 20.6)	29.1(24.6, 33.7)	30.5(22.9, 38.0)
Malawi	2015	40.2(35.1, 45.2)	36.7(30.1, 43.2)	-8.6(-26.3, 9.1)	55.1(52.7, 57.4)	59.2(57.3, 61.2)	-1.4(-6.5, 3.8)
Mali	2015	1.1(0.0, 2.4)	19.1(14.3, 23.9)	0.4(0.3, 0.4)	3.4(2.5, 4.2)	31.7(29.6, 33.9)	0.6(0.5, 0.6)
Mauritania	2015	3.8(0.8, 6.8)	13.4(5.1, 21.8)	0.2(0.1, 0.3)	6.2(4.4, 7.9)	29.5(26.7, 32.3)	0.4(0.4, 0.4)
Mexico	2015	50.7(42.4, 59.0)	73.9(54.5, 93.3)	0.3(0.0, 0.6)	63.7(61.1, 66.4)	68.3(63.8, 72.7)	0.2(0.1, 0.3)
Moldova	2012	14.3(0.0, 35.3)	42.9(13.2, 72.5)	0.5(0.0, 1.1)	34.9(27.7, 42.0)	47.6(44.1, 51.1)	0.2(0.1, 0.3)
Mongolia	2013	31.0(14.7, 47.2)	24.2(0.0, 53.8)	-0.1(-0.5, 0.3)	47.8(44.7, 50.9)	36.4(32.9, 39.8)	-0.1(-0.2, -0.1)
Mozambique	2011	1.8(0.0, 3.6)	36.9(31.9, 41.9)	62.9(56.1, 69.7)	3.2(2.0, 4.4)	38.0(35.8, 40.3)	56.5(52.7, 60.2)
Myanmar	2016	60.9(48.7, 73.1)	48.0(29.1, 66.9)	-3.1(-40.7, 34.5)	49.7(46.4, 53.0)	56.8(52.8, 60.9)	12.7(4.2, 21.2)
Namibia	2013	11.7(0.5, 22.8)	52.7(39.6, 65.9)	54.4(31.8, 77.0)	53.4(48.4, 58.5)	61.9(57.6, 66.1)	0.1(-7.6, 7.8)
Nepal	2016	30.5(25.6, 35.4)	38.5(25.7, 51.4)	8.1(-6.7, 22.8)	49.4(46.6, 52.3)	49.8(46.6, 52.9)	-3.1(-9.1, 3.0)
Niger	2012	0.0(0.0, 0.0)	13.2(8.7, 17.8)	53.6(40.9, 66.3)	3.0(1.9, 4.0)	25.9(23.8, 28.1)	67.9(61.3, 74.5)
Nigeria	2017	0.5(0.0, 1.1)	3.9(0.0, 9.4)	0.1(0.1, 0.2)	2.3(1.7, 2.9)	18.3(16.8, 19.8)	0.3(0.3, 0.4)
Pakistan	2012	0.7(0.0, 2.2)	7.0(0.9, 13.1)	16.6(5.7, 27.4)	11.3(9.6, 13.0)	29.6(27.3, 31.8)	28.2(23.8, 32.6)
Panama	2013	27.7(22.4, 33.0)	66.7(0.0, 100.0)	0.2(0.0, 0.4)	38.8(36.2, 41.4)	69.9(64.3, 75.5)	0.4(0.4, 0.5)
Paraguay	2016	62.6(53.3, 71.9)	60.0(23.1, 96.9)	0.5(0.1, 0.8)	75.5(72.0, 79.0)	65.9(61.3, 70.4)	-0.2(-0.3, -0.1)
Peru	2012	38.7(32.8, 44.5)	63.2(51.5, 75.0)	33.3(20.3, 46.4)	46.9(44.5, 49.4)	62.2(58.9, 65.5)	22.6(18.1, 27.1)
Philippines	2017	25.7(19.3, 32.0)	41.7(20.4, 62.9)	18.9(-6.5, 44.2)	46.4(44.4, 48.4)	31.5(28.3, 34.7)	-32.1(-38.0, -26.2)
Rwanda	2015	45.7(30.1, 61.4)	47.2(34.3, 60.2)	-11.9(-52.7, 28.8)	46.5(43.2, 49.7)	49.2(45.9, 52.4)	1.6(-8.0, 11.1)
Sao Tome and Principe	2014	28.9(15.1, 42.7)	50.0(0.0, 100.0)	-0.1(-0.8, 0.6)	37.3(30.6, 43.9)	37.0(29.3, 44.7)	0.1(-0.1, 0.3)
Senegal	2017	7.2(4.7, 9.6)	15.4(3.5, 27.2)	11.8(4.1, 19.6)	17.4(15.6, 19.2)	35.1(31.5, 38.7)	36.5(31.9, 41.1)
Serbia	2014	100.0(100.0, 100.0)	100.0(100.0, 100.0)	0.0(-0.4, 0.5)	11.7(7.8, 15.7)	25.0(20.9, 29.1)	0.3(0.1, 0.5)

© 2020 Li Z et al. *JAMA Network Open.*

eTable 4. Prevalence of modern contraceptive use among adolescents 15-19 years old and adult women 20-34 years old in poorest wealth quintile (Q1), richest wealth quintile (Q5), and slope index of inequality (SII), using the most recent years of data available (continued)

		15-19			20-34		
**Country**	**Year**	**Q1 (%)**	**Q5 (%)**	**SII**	**Q1 (%)**	**Q5 (%)**	**SII**
Sierra Leone	2013	-7.5(0.0, -2.9)	23.1(17.8, 28.4)	104.6(83.6, 125.5)	12.6(10.8, 14.4)	40.1(37.7, 42.6)	82.6(75.2, 90.1)
Sudan	2014	1.1(0.3, 1.9)	1.0(0.3, 1.8)	-0.0(-0.0, 0.0)	2.3(1.6, 3.0)	15.1(13.4, 16.8)	0.2(0.2, 0.3)
Suriname	2010	41.1(31.6, 50.5)	43.1(0.0, 98.7)	0.9(0.4, 1.3)	26.9(23.1, 30.7)	53.1(46.3, 59.9)	0.5(0.3, 0.6)
Swaziland	2014	42.1(17.7, 66.6)	100.0(100.0, 100.0)	-0.5(-1.4, 0.3)	70.8(64.3, 77.3)	76.6(70.7, 82.6)	0.2(0.0, 0.3)
Tajikistan	2017	9.8(9.8, 9.8)	11.6(7.9, 15.3)	2.3(-10.2, 14.8)	20.3(17.3, 23.3)	28.3(25.8, 30.9)	23.9(14.7, 33.2)
Tanzania	2015	10.2(6.3, 14.1)	24.5(16.0, 33.0)	33.1(14.4, 51.8)	22.6(19.8, 25.4)	36.6(33.7, 39.4)	25.4(17.3, 33.4)
Thailand	2012	69.8(59.9, 79.7)	70.0(56.8, 83.2)	0.2(-0.1, 0.5)	81.0(78.2, 83.9)	70.5(68.2, 72.8)	-0.2(-0.2, -0.1)
Timor-Leste	2016	15.9(9.7, 22.1)	24.7(8.4, 41.0)	22.7(3.7, 41.6)	29.1(25.7, 32.5)	24.6(21.6, 27.6)	-6.4(-13.3, 0.6)
Togo	2014	8.9(3.8, 14.1)	32.1(19.1, 45.1)	45.4(16.4, 74.5)	13.4(11.2, 15.6)	26.5(23.5, 29.6)	28.1(20.0, 36.3)
Turkmenistan	2016	100.0(100.0, 100.0)	100.0(100.0, 100.0)	0.1(-0.1, 0.4)	33.2(28.4, 37.9)	37.2(33.4, 41.0)	-0.0(-0.1, 0.1)
Uganda	2016	13.7(9.8, 17.5)	36.3(27.7, 44.9)	51.2(33.4, 69.0)	21.4(19.4, 23.4)	41.7(39.2, 44.2)	31.2(24.8, 37.5)
Vietnam	2011	22.1(12.6, 31.6)	10.0(0.0, 32.6)	-0.1(-0.4, 0.2)	65.0(61.8, 68.3)	60.6(57.2, 64.0)	-0.1(-0.2, -0.0)
West Bank and Gaza Strip	2014	8.1(2.6, 13.5)	9.1(0.0, 22.1)	-0.1(-0.3, 0.1)	32.4(29.3, 35.4)	43.3(39.7, 46.9)	0.2(0.1, 0.3)
Yemen	2013	1.6(0.0, 3.3)	20.1(13.8, 26.4)	28.4(20.7, 36.1)	8.3(6.9, 9.7)	38.8(36.4, 41.1)	48.4(44.8, 52.1)
Zambia	2013	33.9(28.0, 39.9)	37.7(26.3, 49.1)	33.3(7.3, 59.4)	32.7(29.9, 35.4)	54.8(51.9, 57.7)	47.5(39.3, 55.6)
Zimbabwe	2015	48.1(37.9, 58.3)	41.7(26.7, 56.7)	-12.8(-43.8, 18.2)	65.3(61.6, 68.9)	69.9(67.0, 72.9)	11.1(3.4, 18.8)

© 2020 Li Z et al. *JAMA Network Open.*

eTable 5. Prevalence of unmet need for family planning among adolescents 15-19 years old and adult women 20-34 years old in poorest wealth quintile (Q1), richest wealth quintile (Q5), and slope index of inequality (SII), using the most recent years of data available

		15-19			20-34		
Country	Year	Q1 (%)	Q5 (%)	SII	Q1 (%)	Q5 (%)	SII
Afghanistan	2015	90.5(84.8, 96.2)	0.7(0.6, 0.7)	-0.9(-1.1, -0.6)	72.0(69.6, 74.5)	0.5(0.4, 0.5)	-0.7(-0.8, -0.6)
Albania	2017	55.9(38.3, 73.5)	0.3(0.0, 0.7)	-0.7(-1.5, 0.1)	48.1(43.9, 52.3)	0.4(0.3, 0.5)	-0.3(-0.4, -0.1)
Algeria	2012	100.0(100.0, 100.0)	100.0(100.0, 100.0)	-0.5(-3.8, 2.8)	24.5(16.2, 32.9)	7.9(1.1, 14.8)	-0.3(-0.5, -0.1)
Angola	2015	96.6(93.3, 99.9)	0.6(0.5, 0.7)	-0.5(-0.6, -0.4)	95.7(93.6, 97.8)	0.5(0.4, 0.5)	-0.9(-0.9, -0.8)
Armenia	2016	33.3(0.0, 87.5)	100.0(100.0, 100.0)	0.3(-1.3, 1.9)	21.0(16.3, 25.8)	0.2(0.1, 0.2)	-0.0(-0.2, 0.1)
Bangladesh	2014	25.3(20.0, 30.6)	0.2(0.2, 0.3)	-0.2(-0.3, -0.0)	15.5(13.6, 17.4)	0.2(0.1, 0.2)	-0.0(-0.1, 0.0)
Belize	2016	63.6(29.7, 97.5)	100.0(100.0, 100.0)	-0.8(-1.6, -0.0)	60.4(46.1, 74.8)	37.5(10.9, 64.1)	-0.3(-0.6, 0.0)
Benin	2014	91.7(73.3, 100.0)	50.0(26.0, 74.0)	-0.6(-1.1, -0.1)	85.9(80.3, 91.6)	75.2(70.1, 80.2)	-0.3(-0.4, -0.2)
Burkina Faso	2010	87.3(78.2, 96.4)	0.4(0.4, 0.5)	-1.0(-1.2, -0.8)	77.0(72.9, 81.1)	0.3(0.3, 0.4)	-1.0(-1.1, -0.9)
Burundi	2016	53.6(32.4, 74.7)	22.1(21.9, 22.3)	0.0(-0.5, 0.6)	53.8(49.9, 57.6)	0.4(0.4, 0.4)	-0.3(-0.5, -0.2)
Cambodia	2014	34.1(19.0, 49.3)	0.3(0.2, 0.5)	-0.3(-0.6, 0.1)	19.3(16.7, 21.9)	0.1(0.1, 0.2)	-0.1(-0.2, -0.1)
Cameroon	2011	80.6(70.5, 90.8)	0.4(0.3, 0.4)	-0.6(-0.8, -0.4)	87.5(84.1, 91.0)	0.3(0.2, 0.3)	-1.0(-1.0, -0.9)
Central African Republic	2010	86.2(72.9, 99.6)	64.7(39.4, 90.0)	-0.9(-1.3, -0.4)	83.3(75.7, 90.9)	62.9(52.7, 73.2)	-0.5(-0.7, -0.3)
Chad	2015	89.3(82.5, 96.0)	0.8(0.7, 0.9)	-0.4(-0.6, -0.3)	86.8(83.5, 90.0)	0.7(0.7, 0.7)	-0.5(-0.6, -0.4)
Colombia	2015	27.0(23.2, 30.8)	0.2(0.1, 0.2)	-0.1(-0.2, -0.0)	14.4(13.1, 15.7)	0.1(0.1, 0.1)	-0.1(-0.1, -0.1)
Comoros	2012	90.0(78.6, 100.0)	0.7(0.5, 0.9)	-0.1(-0.5, 0.3)	75.1(69.0, 81.3)	0.5(0.5, 0.6)	-0.4(-0.6, -0.3)
Congo	2011	68.2(62.0, 74.3)	29.8(29.7, 29.9)	-0.1(-0.3, 0.0)	36.1(33.1, 39.0)	0.2(0.1, 0.2)	-0.2(-0.3, -0.2)
Côte d'Ivoire	2016	95.0(84.5, 100.0)	50.0(0.0, 100.0)	-0.2(-0.7, 0.3)	84.5(78.8, 90.3)	55.6(41.9, 69.2)	-0.2(-0.4, -0.1)
Democratic Republic of the Congo	2013	70.7(64.0, 77.3)	0.5(0.4, 0.6)	-0.3(-0.4, -0.1)	71.6(68.6, 74.7)	0.4(0.4, 0.5)	-0.5(-0.5, -0.4)
Dominican Republic	2014	34.4(26.0, 42.7)	22.2(0.0, 56.1)	-0.4(-0.8, -0.1)	27.3(23.4, 31.2)	19.0(12.3, 25.6)	-0.3(-0.5, -0.2)

© 2020 Li Z et al. *JAMA Network Open.*

eTable 5. Prevalence of unmet need for family planning among adolescents 15-19 years old and adult women 20-34 years old in poorest wealth quintile (Q1), richest wealth quintile (Q5), and slope index of inequality (SII), using the most recent years of data available (continued)

		15-19			20-34		
**Country**	**Year**	**Q1 (%)**	**Q5 (%)**	**SII**	**Q1 (%)**	**Q5 (%)**	**SII**
Egypt	2014	33.3(19.5, 47.2)	0.1(0.0, 0.3)	-0.7(-1.4, -0.0)	24.6(22.2, 27.1)	0.1(0.1, 0.1)	-0.4(-0.4, -0.3)
El Salvador	2014	73.7(54.7, 92.7)	75.8(50.3, 100.0)	-0.0(-0.4, 0.4)	63.3(53.2, 73.5)	33.3(22.2, 44.5)	-0.4(-0.6, -0.2)
Ethiopia	2016	80.2(72.6, 87.7)	0.3(0.2, 0.4)	-0.7(-1.1, -0.3)	65.2(61.3, 69.2)	0.2(0.2, 0.2)	-0.7(-0.8, -0.6)
Gabon	2012	58.1(51.4, 64.7)	0.3(0.2, 0.4)	-0.5(-0.7, -0.3)	56.7(52.9, 60.5)	0.3(0.3, 0.4)	-0.5(-0.6, -0.4)
Gambia	2013	90.2(80.8, 99.7)	0.8(0.6, 1.0)	-0.3(-0.6, 0.0)	83.1(78.6, 87.6)	0.6(0.5, 0.6)	-0.5(-0.6, -0.4)
Ghana	2014	100.0(100.0, 100.0)	40.7(40.5, 40.9)	-0.2(-0.7, 0.3)	56.3(51.6, 61.0)	0.4(0.4, 0.5)	-0.4(-0.5, -0.2)
Guatemala	2015	50.3(43.1, 57.4)	0.3(0.2, 0.3)	-0.5(-0.6, -0.3)	32.5(29.9, 35.2)	0.1(0.1, 0.1)	-0.4(-0.4, -0.3)
Guinea	2012	95.0(84.5, 100.0)	66.7(0.0, 100.0)	-0.0(-0.3, 0.3)	86.9(80.1, 93.6)	84.7(76.2, 93.2)	-0.0(-0.2, 0.1)
Guyana	2014	73.3(48.0, 98.7)	50.0(0.0, 100.0)	-0.5(-1.1, 0.2)	53.4(43.6, 63.2)	38.1(15.4, 60.7)	-0.4(-0.7, -0.1)
Haiti	2017	74.0(66.1, 81.8)	0.7(0.5, 0.8)	-0.1(-0.3, 0.1)	56.1(52.6, 59.7)	0.5(0.5, 0.5)	-0.1(-0.2, -0.1)
Honduras	2012	27.2(22.1, 32.3)	0.2(0.1, 0.3)	-0.1(-0.2, 0.0)	17.0(15.1, 18.8)	0.1(0.1, 0.1)	-0.1(-0.1, -0.0)
India	2015	72.3(70.1, 74.5)	0.6(0.5, 0.6)	-0.2(-0.3, -0.1)	36.4(35.9, 37.0)	0.3(0.3, 0.3)	-0.2(-0.2, -0.2)
Indonesia	2017	25.5(19.5, 31.5)	0.3(0.1, 0.5)	-0.4(-0.6, -0.2)	16.7(15.4, 18.0)	0.2(0.1, 0.2)	-0.0(-0.1, -0.0)
Iraq	2011	33.3(0.0, 71.8)	50.0(0.0, 100.0)	-0.9(-2.1, 0.2)	40.1(34.4, 45.9)	14.5(6.7, 22.2)	-0.6(-0.8, -0.3)
Jordan	2012	36.2(20.5, 51.9)	18.0(16.6, 19.4)	-1.2(-2.3, 0.0)	20.4(17.6, 23.1)	0.1(0.1, 0.2)	-0.2(-0.3, -0.1)
Kenya	2014	33.6(24.9, 42.3)	0.2(0.1, 0.3)	-0.6(-0.9, -0.3)	33.8(31.1, 36.5)	0.1(0.1, 0.1)	-0.4(-0.4, -0.3)
Kyrgyzstan	2012	86.1(36.7, 100.0)	44.3(44.3, 44.3)	1.9(0.0, 3.7)	49.4(44.6, 54.2)	21.7(21.6, 21.7)	-0.1(-0.3, 0.1)
Lao People's Democratic Republic	2011	50.0(0.0, 100.0)	83.3(40.5, 100.0)	0.1(-0.7, 0.9)	77.3(68.8, 85.8)	70.6(54.5, 86.7)	-0.1(-0.3, 0.1)
Lesotho	2014	48.0(33.7, 62.3)	0.3(0.1, 0.5)	-0.3(-0.7, -0.0)	29.3(24.7, 33.8)	0.2(0.1, 0.2)	-0.2(-0.3, -0.1)
Liberia	2013	81.4(75.2, 87.6)	0.7(0.6, 0.8)	-0.3(-0.5, -0.1)	66.6(62.9, 70.3)	0.5(0.4, 0.6)	-0.4(-0.5, -0.3)
Malawi	2015	37.1(30.9, 43.2)	0.5(0.4, 0.6)	0.4(0.1, 0.6)	28.3(25.9, 30.7)	0.2(0.2, 0.2)	-0.1(-0.1, -0.0)
Mali	2015	100.0(100.0, 100.0)	63.6(29.7, 97.5)	-0.9(-1.5, -0.4)	95.1(91.5, 98.7)	65.6(57.3, 74.0)	-0.5(-0.7, -0.4)
Mauritania	2015	63.6(36.5, 90.8)	81.8(81.8, 81.8)	-0.4(-1.0, 0.2)	89.0(83.6, 94.3)	63.1(51.0, 75.1)	-0.4(-0.6, -0.3)
Mexico	2015	36.8(20.8, 52.9)	9.1(0.0, 29.3)	-0.4(-0.8, 0.1)	33.0(26.6, 39.4)	25.0(11.0, 39.0)	-0.3(-0.4, -0.1)
Moldova	2012	40.0(0.0, 100.0)	40.0(0.0, 100.0)	-0.4(-2.0, 1.3)	33.6(0.0, 100.0)	11.4(0.0, 34.3)	0.1(-0.4, 0.6)

© 2020 Li Z et al. *JAMA Network Open.*

eTable 5. Prevalence of unmet need for family planning among adolescents 15-19 years old and adult women 20-34 years old in poorest wealth quintile (Q1), richest wealth quintile (Q5), and slope index of inequality (SII), using the most recent years of data available (continued)

		15-19			20-34		
**Country**	**Year**	**Q1 (%)**	**Q5 (%)**	**SII**	**Q1 (%)**	**Q5 (%)**	**SII**
Mozambique	2011	91.8(83.9, 99.8)	0.5(0.5, 0.6)	-0.7(-0.8, -0.6)	86.0(81.1, 90.9)	0.4(0.4, 0.4)	-0.7(-0.8, -0.6)
Myanmar	2016	35.7(20.6, 50.8)	0.4(0.1, 0.7)	-0.1(-0.5, 0.4)	27.0(23.5, 30.6)	0.2(0.1, 0.2)	-0.2(-0.3, -0.1)
Namibia	2013	43.8(31.3, 56.2)	0.1(0.0, 0.3)	-0.4(-0.6, -0.2)	28.6(23.3, 33.8)	0.2(0.1, 0.2)	-0.1(-0.2, -0.0)
Nepal	2016	68.7(60.7, 76.6)	0.5(0.3, 0.7)	-0.4(-0.7, -0.2)	42.7(39.3, 46.1)	0.3(0.3, 0.3)	-0.2(-0.3, -0.1)
Niger	2012	67.6(51.7, 83.4)	0.5(0.4, 0.6)	-0.9(-1.4, -0.3)	64.1(58.6, 69.7)	0.3(0.3, 0.4)	-0.9(-1.0, -0.7)
Nigeria	2017	100.0(100.0, 100.0)	80.0(24.5, 100.0)	-0.3(-0.6, -0.1)	91.9(87.7, 96.2)	76.6(71.6, 81.6)	-0.4(-0.5, -0.3)
Pakistan	2012	84.6(69.8, 99.5)	0.6(0.3, 0.8)	-0.3(-0.7, 0.2)	62.7(58.9, 66.5)	0.3(0.3, 0.3)	-0.5(-0.5, -0.4)
Panama	2013	52.2(40.0, 64.5)	100.0(100.0, 100.0)	0.3(-0.1, 0.6)	51.6(45.8, 57.4)	14.3(0.0, 30.6)	-0.4(-0.5, -0.2)
Paraguay	2016	15.8(0.0, 33.8)	100.0(100.0, 100.0)	0.3(-0.1, 0.8)	23.2(11.8, 34.6)	15.2(2.2, 28.1)	-0.1(-0.3, 0.1)
Peru	2012	27.3(21.6, 33.0)	0.2(0.1, 0.3)	-0.2(-0.3, -0.1)	16.9(15.0, 18.9)	0.1(0.1, 0.1)	-0.1(-0.1, -0.1)
Philippines	2017	41.2(32.1, 50.4)	0.3(0.1, 0.6)	0.1(-0.2, 0.4)	23.8(21.7, 25.8)	0.3(0.3, 0.3)	0.0(-0.0, 0.1)
Rwanda	2015	84.0(46.3, 100.0)	34.7(34.6, 34.9)	0.6(0.1, 1.1)	32.8(29.2, 36.4)	0.2(0.2, 0.3)	-0.1(-0.2, -0.0)
Sao Tome and Principe	2014	46.7(18.1, 75.3)	50.0(0.0, 100.0)	0.2(-0.9, 1.3)	50.9(37.5, 64.3)	50.0(24.4, 75.6)	-0.1(-0.6, 0.3)
Senegal	2017	80.1(73.9, 86.4)	0.6(0.3, 0.9)	-0.4(-0.6, -0.2)	58.5(55.0, 62.0)	0.3(0.2, 0.3)	-0.6(-0.6, -0.5)
Sierra Leone	2013	100.0(100.0, 100.0)	48.0(48.0, 48.1)	-0.6(-0.9, -0.4)	60.9(56.7, 65.1)	0.3(0.3, 0.4)	-0.8(-0.9, -0.7)
Sudan	2014	83.3(40.5, 100.0)	66.7(0.0, 100.0)	0.2(-0.6, 1.0)	94.5(90.1, 98.8)	50.0(40.0, 60.0)	-0.8(-1.0, -0.7)
Suriname	2010	51.4(24.9, 77.9)	74.5(74.5, 74.5)	0.1(-0.8, 1.0)	74.7(64.6, 84.7)	20.0(0.0, 75.5)	-0.6(-1.0, -0.3)
Swaziland	2014	40.0(0.0, 100.0)	100.0(100.0, 100.0)	1.2(-0.1, 2.5)	11.1(1.6, 20.7)	17.6(0.0, 37.9)	0.1(-0.2, 0.4)
Tajikistan	2017	100.0(100.0, 100.0)	44.0(43.6, 44.4)	-0.5(-1.2, 0.2)	56.2(51.0, 61.5)	0.5(0.4, 0.5)	-0.2(-0.3, -0.1)
Tanzania	2015	69.0(59.0, 79.1)	0.5(0.4, 0.7)	-0.2(-0.5, 0.1)	53.7(49.2, 58.2)	0.2(0.2, 0.3)	-0.6(-0.7, -0.5)
Thailand	2012	27.8(4.9, 50.7)	25.0(0.0, 53.7)	-0.3(-0.8, 0.1)	11.5(3.2, 19.7)	8.5(1.2, 15.8)	-0.2(-0.4, 0.0)
Timor-Leste	2016	80.0(60.8, 99.2)	0.7(0.4, 1.0)	-0.3(-0.7, 0.2)	50.1(45.3, 55.0)	0.5(0.5, 0.5)	-0.1(-0.2, 0.1)
Togo	2014	84.3(76.4, 92.3)	0.5(0.3, 0.6)	-0.7(-1.1, -0.4)	70.2(66.0, 74.3)	0.5(0.5, 0.6)	-0.4(-0.6, -0.3)
Uganda	2016	71.2(64.3, 78.1)	0.4(0.3, 0.5)	-0.6(-0.9, -0.4)	59.4(56.4, 62.4)	0.3(0.3, 0.3)	-0.6(-0.7, -0.5)
Vietnam	2011	40.0(0.0, 100.0)	100.0(100.0, 100.0)	-0.6(-2.1, 1.0)	42.4(24.6, 60.2)	13.6(0.0, 29.2)	-0.3(-0.6, 0.0)
Yemen	2013	86.7(78.8, 94.5)	0.5(0.4, 0.6)	-0.6(-0.7, -0.4)	74.3(71.4, 77.1)	0.3(0.2, 0.3)	-0.7(-0.8, -0.7)
Zambia	2013	54.1(45.6, 62.6)	0.6(0.4, 0.7)	-0.1(-0.4, 0.2)	38.2(34.6, 41.7)	0.2(0.2, 0.2)	-0.3(-0.4, -0.3)
Zimbabwe	2015	20.6(10.7, 30.4)	0.2(0.1, 0.4)	0.1(-0.2, 0.4)	16.9(13.7, 20.2)	0.1(0.1, 0.1)	-0.1(-0.1, -0.0)

© 2020 Li Z et al. *JAMA Network Open.*

eTable 6. Prevalence of modern contraceptive use among adolescents 15-19 years old and adult women 20-34 years old in urban area, rural area, and absolute inequality between urban and rural areas (Diff), using the most recent years of data available

Country	Year	15-19			20-34		
		Urban(%)	Rural (%)	Diff (percentage points)	Urban(%)	Rural (%)	Diff (percentage points)
Afghanistan	2015	7.5(4.9, 10.0)	4.1(3.0, 5.1)	3.4(1.8, 4.9)	22.2(20.9, 23.5)	12.0(11.5, 12.6)	10.2(9.4, 11.0)
Albania	2017	12.2(2.7, 21.8)	1.1(0.0, 3.1)	11.2(5.0, 17.4)	5.4(4.2, 6.5)	3.1(2.3, 3.9)	2.2(1.3, 3.2)
Algeria	2012	30.8(22.9, 38.8)	23.6(15.6, 31.7)	7.2(-0.8, 15.2)	45.1(43.8, 46.4)	46.1(44.4, 47.8)	-1.0(-2.5, 0.4)
Angola	2015	17.9(15.1, 20.7)	2.5(1.2, 3.9)	15.4(13.0, 17.7)	18.5(17.2, 19.7)	1.5(1.0, 2.0)	17.0(15.9, 18.0)
Armenia	2016	12.6(12.6, 12.6)	26.9(13.0, 40.7)	-14.3(-24.2, -4.4)	28.4(25.8, 31.0)	24.4(21.6, 27.3)	4.0(1.2, 6.7)
Bangladesh	2014	52.6(48.6, 56.6)	44.5(41.9, 47.1)	8.1(5.0, 11.3)	59.4(57.7, 61.1)	55.7(54.5, 56.9)	3.7(2.3, 5.1)
Belarus	2012	54.5(31.9, 77.1)	23.5(1.0, 46.0)	31.0(8.4, 53.6)	57.1(54.9, 59.3)	49.6(45.7, 53.5)	7.5(4.6, 10.4)
Belize	2016	36.4(21.6, 51.2)	35.3(24.9, 45.7)	1.1(-11.5, 13.7)	55.9(52.1, 59.8)	49.9(46.7, 53.2)	6.0(2.5, 9.5)
Benin	2014	9.3(5.1, 13.6)	6.1(3.3, 8.9)	3.3(-0.5, 7.0)	13.7(12.5, 14.9)	8.6(7.6, 9.6)	5.1(3.9, 6.2)
Bhutan	2010	30.5(15.9, 45.1)	29.9(24.5, 35.3)	0.6(-8.6, 9.9)	61.6(59.2, 64.1)	65.7(64.3, 67.2)	-4.1(-5.9, -2.3)
Burkina Faso	2010	18.0(12.8, 23.3)	0.3(0.0, 2.0)	17.7(14.9, 20.5)	33.6(31.6, 35.5)	11.6(10.8, 12.5)	21.9(20.7, 23.2)
Burundi	2016	37.7(24.2, 51.2)	21.4(15.5, 27.2)	16.4(8.0, 24.8)	31.2(28.4, 34.1)	22.3(21.1, 23.5)	9.0(7.2, 10.7)
Cambodia	2014	18.9(11.4, 26.5)	21.5(17.4, 25.5)	-2.6(-7.7, 2.6)	36.4(34.2, 38.6)	45.3(43.9, 46.7)	-8.9(-10.5, -7.3)
Cameroon	2014	15.9(9.8, 21.9)	6.9(3.8, 10.1)	8.9(3.9, 13.9)	24.4(22.3, 26.5)	11.6(10.1, 13.2)	12.7(10.8, 14.6)
Central African Republic	2010	9.8(7.1, 12.6)	2.3(1.3, 3.2)	7.6(5.7, 9.5)	14.9(13.3, 16.5)	2.5(1.9, 3.0)	12.4(11.3, 13.5)
Chad	2015	5.5(3.1, 8.0)	0.8(0.3, 1.3)	4.7(3.4, 5.9)	10.3(8.8, 11.7)	1.8(1.5, 2.1)	8.5(7.7, 9.2)
Colombia	2015	66.0(63.6, 68.4)	55.0(50.8, 59.2)	11.0(8.0, 14.0)	78.0(77.0, 78.9)	70.5(68.8, 72.2)	7.5(6.3, 8.7)
Comoros	2012	17.4(10.5, 24.2)	9.4(4.3, 14.5)	8.0(2.1, 13.8)	20.8(18.0, 23.7)	10.9(9.0, 12.7)	10.0(7.7, 12.2)
Congo	2015	17.5(10.6, 24.4)	16.3(12.2, 20.5)	1.2(-4.4, 6.7)	21.0(18.8, 23.2)	17.4(15.8, 18.9)	3.6(1.7, 5.4)
Costa Rica	2011	65.3(51.5, 79.1)	63.7(53.7, 73.8)	1.6(-10.5, 13.6)	76.1(73.0, 79.3)	73.1(70.1, 76.1)	3.0(-0.0, 6.1)
Cuba	2014	58.5(50.9, 66.1)	61.3(51.6, 71.1)	-2.9(-11.1, 5.3)	69.9(68.3, 71.4)	71.7(69.2, 74.3)	-1.9(-3.7, 0.0)
Côte d'Ivoire	2016	7.7(2.8, 12.6)	4.6(2.4, 6.8)	3.1(-0.6, 6.8)	20.0(17.9, 22.2)	9.8(8.7, 10.8)	10.2(8.6, 11.9)
Democratic Republic of the Congo	2013	9.0(5.8, 12.1)	1.8(0.4, 3.3)	7.1(5.0, 9.2)	14.5(13.1, 15.9)	3.8(3.3, 4.3)	10.7(9.8, 11.5)

© 2020 Li Z et al. *JAMA Network Open.*

eTable 6. Prevalence of modern contraceptive use among adolescents 15-19 years old and adult women 20-34 years old in urban area, rural area, and absolute inequality between urban and rural areas (Diff), using the most recent years of data available (continued)

Country	Year	15-19			20-34		
		Urban(%)	Rural (%)	Diff (percentage points)	Urban(%)	Rural (%)	Diff (percentage points)
Dominican Republic	2014	48.7(45.0, 52.3)	53.7(49.7, 57.8)	-5.1(-8.9, -1.3)	62.2(61.1, 63.4)	65.4(63.9, 66.9)	-3.1(-4.4, -1.9)
Egypt	2014	19.3(13.7, 24.9)	18.5(15.3, 21.7)	0.8(-3.2, 4.8)	58.2(56.8, 59.7)	48.7(47.5, 49.9)	9.6(8.3, 10.8)
El Salvador	2014	58.0(53.4, 62.5)	58.6(53.9, 63.4)	-0.6(-5.3, 4.0)	71.7(69.8, 73.7)	61.5(59.4, 63.6)	10.3(8.2, 12.3)
Ethiopia	2016	37.4(29.0, 45.8)	21.2(17.8, 24.6)	16.2(10.9, 21.5)	49.8(47.4, 52.1)	26.4(25.1, 27.8)	23.3(21.6, 25.0)
Gabon	2012	24.1(19.8, 28.4)	7.1(1.4, 12.8)	17.0(12.2, 21.8)	25.4(23.6, 27.3)	15.6(13.3, 18.0)	9.8(7.8, 11.8)
Gambia	2013	5.1(1.6, 8.6)	1.1(0.1, 2.0)	4.1(1.9, 6.3)	13.6(11.9, 15.3)	4.3(3.5, 5.0)	9.4(8.2, 10.6)
Ghana	2014	16.1(7.8, 24.4)	17.3(11.1, 23.5)	-1.2(-8.4, 6.0)	24.2(22.0, 26.3)	26.8(24.7, 28.9)	-2.6(-4.8, -0.5)
Guatemala	2015	52.1(47.2, 57.0)	37.7(34.8, 40.6)	14.4(10.6, 18.1)	56.3(54.7, 58.0)	43.6(42.2, 44.9)	12.8(11.3, 14.3)
Guinea	2016	7.1(3.5, 10.8)	1.3(0.3, 2.3)	5.9(3.2, 8.5)	8.9(7.4, 10.4)	3.2(2.5, 3.8)	5.7(4.6, 6.8)
Guinea-Bissau	2014	7.8(1.1, 14.6)	1.8(0.0, 3.6)	6.0(1.4, 10.5)	20.7(18.0, 23.4)	6.4(5.4, 7.4)	14.3(12.4, 16.2)
Guyana	2014	8.6(0.0, 26.7)	16.2(8.9, 23.4)	-7.5(-15.1, 0.1)	36.5(30.8, 42.1)	38.9(36.4, 41.5)	-2.5(-6.0, 1.0)
Haiti	2017	39.9(33.9, 45.9)	28.5(24.3, 32.7)	11.4(6.4, 16.3)	36.9(34.7, 39.1)	33.4(31.7, 35.2)	3.5(1.6, 5.4)
Honduras	2012	63.6(59.2, 67.9)	55.7(52.6, 58.9)	7.8(4.2, 11.5)	71.5(69.7, 73.2)	66.4(65.0, 67.8)	5.1(3.5, 6.6)
India	2015	44.4(43.3, 45.6)	42.2(41.8, 42.7)	2.2(1.5, 2.9)	51.9(51.6, 52.3)	48.1(47.9, 48.4)	3.8(3.5, 4.1)
Indonesia	2017	48.8(43.3, 54.4)	46.4(42.6, 50.1)	2.5(-1.9, 6.9)	58.1(56.9, 59.2)	63.1(62.1, 64.1)	-5.0(-6.1, -3.9)
Iraq	2011	12.8(11.0, 14.7)	9.3(7.5, 11.0)	3.6(1.8, 5.4)	31.9(31.1, 32.8)	28.0(27.0, 29.0)	4.0(3.0, 4.9)
Jordan	2012	16.9(12.4, 21.5)	26.7(15.2, 38.1)	-9.7(-16.2, -3.3)	45.0(43.5, 46.6)	41.6(39.3, 43.9)	3.5(1.7, 5.2)
Kazakhstan	2015	29.2(15.8, 42.5)	14.3(3.2, 25.3)	14.9(2.4, 27.3)	53.6(51.6, 55.6)	54.4(51.9, 56.8)	-0.8(-3.0, 1.4)
Kenya	2014	39.0(33.4, 44.7)	26.2(22.6, 29.8)	12.8(8.3, 17.4)	55.3(53.8, 56.7)	46.4(45.3, 47.6)	8.9(7.6, 10.1)
Kosovo	2014	12.5(0.0, 42.1)	100.0(100.0, 100.0)	-87.5(-95.0, -80.0)	12.6(9.7, 15.5)	10.7(8.6, 12.8)	1.9(-0.6, 4.3)
Kyrgyzstan	2014	10.2(2.5, 17.9)	9.2(3.9, 14.5)	1.1(-5.2, 7.4)	39.9(36.8, 43.0)	36.9(34.6, 39.1)	3.0(0.4, 5.6)
Lao People's Democratic Republic	2011	24.7(17.8, 31.6)	20.1(17.7, 22.6)	4.5(0.4, 8.6)	43.1(40.9, 45.3)	47.2(46.0, 48.4)	-4.1(-5.6, -2.6)

© 2020 Li Z et al. *JAMA Network Open.*

eTable 6. Prevalence of modern contraceptive use among adolescents 15-19 years old and adult women 20-34 years old in urban area, rural area, and absolute inequality between urban and rural areas (Diff), using the most recent years of data available (continued)

Country	Year	15-19			20-34		
		Urban(%)	Rural (%)	Diff (percentage points)	Urban(%)	Rural (%)	Diff (percentage points)
Lebanon	2011	25.0(11.7, 38.3)	28.0(9.1, 46.9)	-3.0(-18.5, 12.5)	50.1(46.7, 53.5)	48.6(43.9, 53.3)	1.5(-2.4, 5.4)
Lesotho	2014	46.4(33.5, 59.2)	24.2(18.4, 30.0)	22.1(13.7, 30.5)	69.9(66.6, 73.2)	62.6(60.3, 65.0)	7.3(4.6, 10.0)
Liberia	2013	20.1(15.5, 24.8)	6.3(3.1, 9.5)	13.8(9.9, 17.7)	25.1(22.8, 27.4)	18.4(16.7, 20.1)	6.7(4.7, 8.7)
Malawi	2015	39.9(33.2, 46.5)	35.1(32.5, 37.8)	4.7(0.9, 8.5)	63.4(61.3, 65.5)	63.0(61.9, 64.1)	0.4(-1.0, 1.7)
Mali	2015	15.9(11.5, 20.2)	5.9(4.6, 7.2)	10.0(7.2, 12.7)	29.6(27.6, 31.6)	11.2(10.4, 12.0)	18.4(17.1, 19.7)
Mauritania	2015	13.6(9.0, 18.3)	5.6(3.5, 7.8)	8.0(4.4, 11.6)	27.3(25.4, 29.1)	10.1(8.9, 11.3)	17.2(15.6, 18.7)
Mexico	2015	54.0(48.1, 60.0)	46.8(39.1, 54.5)	7.2(0.6, 13.9)	65.4(63.7, 67.2)	63.6(61.2, 65.9)	1.9(-0.1, 3.9)
Moldova	2012	41.5(25.7, 57.2)	31.7(16.8, 46.6)	9.8(-5.6, 25.1)	45.4(42.5, 48.2)	40.6(37.2, 44.1)	4.7(1.6, 7.8)
Mongolia	2013	30.6(17.2, 44.0)	21.9(6.7, 37.0)	8.7(-5.2, 22.7)	36.6(34.6, 38.6)	47.6(45.2, 49.9)	-11.0(-13.1, -8.9)
Mozambique	2011	27.9(24.1, 31.6)	5.4(4.0, 6.9)	22.5(19.8, 25.1)	31.9(30.0, 33.7)	9.6(8.6, 10.6)	22.3(20.8, 23.7)
Myanmar	2016	58.3(45.5, 71.2)	44.4(37.0, 51.8)	14.0(4.9, 23.0)	58.8(55.6, 62.0)	51.4(49.6, 53.3)	7.4(5.2, 9.7)
Namibia	2013	35.9(27.5, 44.3)	28.5(21.2, 35.8)	7.4(-0.5, 15.3)	65.9(63.4, 68.3)	59.2(56.2, 62.2)	6.7(4.0, 9.4)
Nepal	2016	36.6(33.0, 40.2)	32.5(28.7, 36.2)	4.2(0.5, 7.8)	51.5(49.9, 53.2)	47.6(45.6, 49.7)	3.9(2.1, 5.7)
Niger	2012	13.9(8.6, 19.3)	2.7(1.6, 3.8)	11.2(8.5, 13.9)	27.6(25.3, 30.0)	6.7(6.0, 7.5)	20.9(19.6, 22.2)
Nigeria	2017	5.7(1.5, 9.8)	1.0(0.5, 1.6)	4.7(2.2, 7.1)	16.3(15.1, 17.5)	6.9(6.4, 7.4)	9.4(8.6, 10.2)
Pakistan	2012	10.8(6.6, 15.1)	4.9(2.6, 7.2)	5.9(2.8, 9.1)	36.1(34.6, 37.7)	25.8(24.6, 27.0)	10.3(9.0, 11.6)
Panama	2013	32.5(22.0, 43.1)	23.3(18.3, 28.2)	9.3(1.7, 16.8)	64.2(61.5, 66.9)	48.8(46.5, 51.0)	15.4(13.0, 17.9)
Paraguay	2016	62.4(54.1, 70.7)	68.6(60.8, 76.4)	-6.2(-14.3, 1.9)	72.3(70.1, 74.6)	76.4(73.9, 78.8)	-4.0(-6.4, -1.7)
Peru	2012	55.8(51.5, 60.1)	43.4(38.4, 48.4)	12.4(7.8, 17.0)	57.7(56.3, 59.1)	50.1(48.2, 52.0)	7.6(6.0, 9.2)
Philippines	2017	43.6(36.0, 51.3)	45.3(40.5, 50.1)	-1.7(-7.6, 4.2)	41.1(39.2, 43.1)	45.5(44.1, 46.9)	-4.4(-6.0, -2.8)
Rwanda	2015	49.9(36.4, 63.5)	43.0(35.0, 51.1)	6.9(-3.2, 17.0)	53.3(50.3, 56.4)	51.2(49.5, 52.9)	2.1(-0.1, 4.3)
Sao Tome and Principe	2014	16.1(6.1, 26.0)	41.1(27.8, 54.4)	-25.0(-36.3, -13.7)	39.2(35.3, 43.2)	47.5(42.5, 52.5)	-8.3(-12.7, -3.8)

© 2020 Li Z et al. *JAMA Network Open.*

eTable 6. Prevalence of modern contraceptive use among adolescents 15-19 years old and adult women 20-34 years old in urban area, rural area, and absolute inequality between urban and rural areas (Diff), using the most recent years of data available (continued)

Country	Year	15-19			20-34		
		Urban(%)	Rural (%)	Diff (percentage points)	Urban(%)	Rural (%)	Diff (percentage points)
Senegal	2017	20.0(15.9, 24.1)	14.0(12.3, 15.7)	6.1(3.3, 8.8)	31.8(30.0, 33.7)	18.3(17.1, 19.5)	13.5(12.1, 15.0)
Sierra Leone	2013	5.8(2.0, 9.5)	10.8(8.1, 13.4)	-5.0(-8.2, -1.8)	35.9(34.0, 37.7)	13.9(12.8, 14.9)	22.0(20.5, 23.4)
Sudan	2014	5.7(5.1, 6.2)	5.9(5.5, 6.4)	-0.3(-0.8, 0.2)	14.6(13.5, 15.8)	9.8(9.2, 10.4)	4.9(4.0, 5.7)
Suriname	2010	46.0(31.7, 60.3)	33.3(23.7, 42.9)	12.7(0.8, 24.6)	46.2(42.3, 50.1)	40.0(36.9, 43.0)	6.2(2.8, 9.7)
Swaziland	2014	42.9(0.0, 92.3)	50.0(32.3, 67.7)	-7.1(-30.3, 16.0)	77.6(72.4, 82.8)	69.7(66.4, 73.1)	7.9(4.2, 11.5)
Tajikistan	2017	10.1(10.1, 10.1)	11.3(9.6, 13.1)	-1.3(-2.6, 0.1)	33.3(31.0, 35.5)	28.1(26.6, 29.7)	5.1(3.3, 7.0)
Tanzania	2015	14.1(8.2, 19.9)	12.5(9.6, 15.4)	1.6(-2.3, 5.5)	38.6(36.1, 41.1)	30.0(28.5, 31.6)	8.5(6.7, 10.4)
Thailand	2012	68.0(62.1, 73.9)	69.8(64.1, 75.5)	-1.8(-7.6, 4.0)	72.4(71.0, 73.8)	73.8(72.3, 75.3)	-1.5(-2.9, -0.0)
The former Yugoslav Republic of Macedonia	2011	12.5(0.0, 42.1)	4.8(0.0, 14.7)	7.7(-0.6, 16.1)	15.2(12.4, 18.0)	10.8(8.4, 13.2)	4.5(1.9, 7.0)
Timor-Leste	2016	23.2(13.1, 33.3)	17.4(13.7, 21.1)	5.8(-0.0, 11.5)	24.3(22.0, 26.6)	27.7(26.0, 29.3)	-3.4(-5.2, -1.6)
Togo	2014	20.1(12.0, 28.1)	8.9(4.8, 13.0)	11.2(5.6, 16.8)	26.0(23.8, 28.3)	17.1(15.6, 18.6)	8.9(7.2, 10.7)
Tunisia	2012	2.2(0.0, 26.1)	100.0(100.0, 100.0)	-97.8(-104.2, -71.3)	45.1(42.1, 48.1)	49.5(45.8, 53.2)	-4.4(-7.7, -1.1)
Turkmenistan	2016	7.7(0.0, 24.5)	2.0(0.0, 6.1)	5.7(0.3, 11.0)	36.1(33.3, 38.8)	40.0(37.5, 42.5)	-3.9(-6.5, -1.3)
Uganda	2016	32.0(24.9, 39.0)	20.3(17.7, 22.9)	11.7(7.8, 15.7)	40.4(38.0, 42.7)	32.8(31.6, 34.0)	7.6(6.1, 9.1)
Vietnam	2011	11.1(0.3, 21.9)	19.8(12.6, 27.0)	-8.7(-17.6, 0.1)	59.2(56.8, 61.7)	61.0(59.0, 63.0)	-1.8(-4.0, 0.4)
West Bank and Gaza Strip	2014	6.2(2.9, 9.6)	10.2(2.2, 18.1)	-3.9(-9.0, 1.2)	34.7(33.1, 36.4)	39.0(36.2, 41.8)	-4.3(-6.3, -2.2)
Yemen	2013	17.2(12.3, 22.0)	6.7(5.0, 8.4)	10.5(7.6, 13.4)	37.8(35.9, 39.6)	19.9(18.9, 20.9)	17.9(16.6, 19.1)
Zambia	2013	41.2(35.8, 46.6)	30.4(26.9, 33.9)	10.8(6.3, 15.3)	52.5(50.6, 54.3)	39.5(37.8, 41.1)	13.0(11.3, 14.8)
Zimbabwe	2015	43.8(34.2, 53.5)	48.3(42.9, 53.7)	-4.5(-11.8, 2.8)	72.3(70.1, 74.5)	69.4(67.4, 71.4)	2.9(0.8, 5.0)

© 2020 Li Z et al. *JAMA Network Open.*

eTable 7. Prevalence of unmet need for family planning among adolescents 15-19 years old and adult women 20-34 years old in urban area, rural area, and absolute inequality between urban and rural areas (Diff), using the most recent years of data available

Country	Year	15-19			20-34		
		Urban(%)	Rural (%)	Diff (percentage points)	Urban(%)	Rural (%)	Diff (percentage points)
Afghanistan	2015	72.0(65.3, 78.7)	74.8(70.7, 78.8)	-2.7(-7.5, 2.1)	53.1(51.0, 55.1)	64.6(63.4, 65.9)	-11.6(-13.0, -10.1)
Albania	2017	37.8(21.4, 54.2)	47.5(34.6, 60.4)	-9.7(-24.2, 4.8)	43.1(40.1, 46.2)	42.4(39.5, 45.3)	0.7(-2.2, 3.7)
Algeria	2012	33.3(0.0, 100.0)	33.3(0.0, 100.0)	0.0(-33.3, 33.3)	17.3(13.0, 21.5)	19.2(12.7, 25.6)	-1.9(-7.0, 3.1)
Angola	2015	72.1(68.2, 76.0)	93.6(90.7, 96.6)	-21.5(-25.1, -17.9)	66.2(64.2, 68.1)	94.4(92.9, 95.9)	-28.3(-30.1, -26.5)
Armenia	2016	33.3(0.0, 100.0)	25.0(0.0, 53.7)	8.3(-21.0, 37.7)	20.1(17.3, 22.9)	18.5(15.5, 21.5)	1.6(-1.4, 4.5)
Bangladesh	2014	20.4(16.6, 24.2)	27.3(24.4, 30.2)	-6.8(-10.1, -3.6)	11.4(10.1, 12.6)	17.9(16.8, 19.0)	-6.5(-7.7, -5.4)
Belize	2016	46.2(14.8, 77.5)	47.6(24.3, 70.9)	-1.5(-28.8, 25.8)	35.6(24.4, 46.9)	42.6(33.4, 51.8)	-7.0(-17.2, 3.2)
Benin	2014	77.1(64.8, 89.4)	81.8(71.3, 92.3)	-4.7(-16.3, 6.9)	78.5(75.3, 81.8)	83.8(80.4, 87.1)	-5.2(-8.5, -1.9)
Burkina Faso	2010	47.7(40.3, 55.2)	78.5(73.5, 83.6)	-30.8(-36.6, -25.0)	37.6(35.0, 40.2)	67.4(65.4, 69.4)	-29.8(-32.0, -27.7)
Burundi	2016	35.3(18.4, 52.2)	39.0(27.8, 50.1)	-3.7(-16.5, 9.1)	38.2(34.4, 42.0)	52.2(50.3, 54.0)	-14.0(-16.5, -11.5)
Cambodia	2014	9.0(0.0, 23.0)	8.5(0.8, 16.3)	0.5(-7.6, 8.6)	14.1(12.3, 16.0)	16.2(14.9, 17.4)	-2.1(-3.5, -0.6)
Cameroon	2014	21.4(0.0, 46.0)	48.1(28.0, 68.3)	-26.7(-47.6, -5.8)	45.2(37.0, 53.4)	46.0(37.1, 54.9)	-0.8(-9.3, 7.7)
Central African Republic	2010	73.8(62.4, 85.1)	88.6(81.4, 95.8)	-14.8(-23.9, -5.8)	72.1(66.6, 77.6)	84.4(80.6, 88.2)	-12.3(-16.8, -7.7)
Chad	2015	84.4(78.6, 90.2)	92.5(89.6, 95.4)	-8.1(-11.9, -4.3)	71.6(68.3, 74.9)	88.4(86.9, 89.9)	-16.8(-18.9, -14.7)
Colombia	2015	17.8(15.6, 19.9)	20.0(16.2, 23.9)	-2.3(-5.0, 0.4)	10.1(9.4, 10.7)	13.1(11.8, 14.4)	-3.0(-3.9, -2.2)
Comoros	2012	64.2(54.4, 74.0)	77.6(68.0, 87.2)	-13.4(-23.1, -3.7)	47.7(43.3, 52.2)	69.0(65.4, 72.6)	-21.2(-25.2, -17.3)
Congo	2015	53.1(34.8, 71.4)	64.4(53.1, 75.6)	-11.3(-26.0, 3.5)	66.0(59.7, 72.4)	61.5(57.3, 65.7)	4.5(-0.7, 9.8)
Costa Rica	2011	18.2(0.0, 45.4)	26.3(4.5, 48.1)	-8.1(-28.3, 12.0)	20.2(11.7, 28.7)	13.4(6.5, 20.3)	6.8(-0.9, 14.5)
Cuba	2014	16.7(0.0, 59.5)	9.1(0.0, 29.3)	7.6(-7.5, 22.7)	16.1(9.2, 23.0)	22.0(10.1, 33.9)	-5.9(-14.4, 2.6)

© 2020 Li Z et al. *JAMA Network Open.*

eTable 7. Prevalence of unmet need for family planning among adolescents 15-19 years old and adult women 20-34 years old in urban area, rural area, and absolute inequality between urban and rural areas (Diff), using the most recent years of data available (continued)

Country	Year	15-19			20-34		
		Urban(%)	Rural (%)	Diff (percentage points)	Urban(%)	Rural (%)	Diff (percentage points)
Côte d'Ivoire	2016	87.5(69.3, 100.0)	87.8(77.3, 98.3)	-0.3(-14.9, 14.3)	74.0(68.0, 80.1)	83.2(79.5, 86.9)	-9.2(-14.1, -4.2)
Democratic Republic of the Congo	2013	65.1(59.5, 70.7)	76.3(72.3, 80.3)	-11.2(-15.7, -6.7)	49.9(47.5, 52.3)	68.5(66.6, 70.3)	-18.6(-20.6, -16.5)
Dominican Republic	2014	30.2(23.2, 37.2)	26.5(18.4, 34.6)	3.7(-3.7, 11.1)	23.4(20.6, 26.2)	22.9(19.2, 26.5)	0.5(-2.6, 3.7)
Egypt	2014	23.3(10.1, 36.4)	30.7(23.5, 37.8)	-7.4(-16.7, 1.9)	15.0(13.7, 16.2)	20.4(19.2, 21.6)	-5.4(-6.6, -4.2)
El Salvador	2014	79.7(70.0, 89.4)	66.7(51.8, 81.5)	13.0(0.7, 25.4)	43.0(36.6, 49.5)	59.5(51.5, 67.5)	-16.4(-23.6, -9.2)
Ethiopia	2016	36.9(26.4, 47.4)	53.2(47.2, 59.3)	-16.3(-23.9, -8.7)	19.9(17.6, 22.3)	43.8(41.6, 45.9)	-23.8(-26.0, -21.6)
Gabon	2012	52.8(48.0, 57.5)	71.6(64.1, 79.1)	-18.8(-24.6, -13.0)	43.3(40.8, 45.8)	55.0(51.0, 58.9)	-11.7(-14.7, -8.6)
Gambia	2013	80.9(69.2, 92.5)	92.5(87.0, 97.9)	-11.6(-19.8, -3.4)	63.4(59.6, 67.2)	84.0(81.4, 86.5)	-20.5(-23.6, -17.5)
Ghana	2014	69.8(59.9, 79.7)	72.4(64.6, 80.2)	-2.7(-11.4, 6.1)	48.4(45.1, 51.6)	51.2(48.2, 54.2)	-2.8(-5.9, 0.3)
Guatemala	2015	30.4(24.9, 35.8)	37.8(33.9, 41.8)	-7.5(-12.0, -2.9)	15.4(14.1, 16.8)	25.5(24.1, 26.9)	-10.1(-11.5, -8.7)
Guinea	2012	80.1(72.0, 88.2)	98.3(92.9, 100.0)	-18.2(-24.7, -11.7)	60.7(56.6, 64.7)	81.3(78.4, 84.1)	-20.6(-23.9, -17.3)
Guinea-Bissau	2014	100.0(100.0, 100.0)	100.0(100.0, 100.0)	0.0(0.0, 0.0)	66.0(52.4, 79.6)	75.5(66.8, 84.2)	-9.5(-20.5, 1.4)
Guyana	2014	60.0(0.0, 100.0)	68.8(51.8, 85.7)	-8.7(-41.1, 23.6)	43.5(28.6, 58.4)	47.1(40.2, 54.0)	-3.6(-12.9, 5.8)
Haiti	2017	61.7(55.3, 68.0)	74.8(70.1, 79.6)	-13.2(-18.6, -7.7)	49.7(47.2, 52.2)	54.8(52.7, 56.9)	-5.1(-7.4, -2.8)
Honduras	2012	22.9(18.8, 26.9)	24.7(21.4, 27.9)	-1.8(-5.4, 1.8)	12.2(10.9, 13.5)	14.9(13.8, 16.0)	-2.7(-3.9, -1.5)
India	2015	61.1(58.2, 64.0)	65.2(63.9, 66.5)	-4.1(-6.0, -2.3)	27.8(27.4, 28.2)	30.6(30.4, 30.9)	-2.8(-3.1, -2.5)
Indonesia	2017	13.0(6.8, 19.2)	12.0(7.8, 16.3)	0.9(-4.1, 6.0)	15.3(14.3, 16.3)	13.6(12.8, 14.5)	1.7(0.8, 2.6)
Iraq	2011	40.0(11.9, 68.1)	27.3(0.0, 58.7)	12.7(-15.0, 40.5)	23.9(20.3, 27.5)	33.4(28.4, 38.5)	-9.5(-13.8, -5.2)
Jordan	2012	25.5(13.4, 37.7)	28.7(7.6, 49.7)	-3.1(-17.4, 11.1)	16.3(14.9, 17.7)	14.9(12.8, 17.0)	1.4(-0.2, 2.9)
Kazakhstan	2010	100.0(100.0, 100.0)	50.0(0.0, 100.0)	50.0(16.8, 83.2)	31.0(13.1, 48.9)	35.1(19.0, 51.3)	-4.1(-21.3, 13.1)

© 2020 Li Z et al. *JAMA Network Open.*

eTable 7. Prevalence of unmet need for family planning among adolescents 15-19 years old and adult women 20-34 years old in urban area, rural area, and absolute inequality between urban and rural areas (Diff), using the most recent years of data available (continued)

Country	Year	15-19			20-34		
		Urban(%)	Rural (%)	Diff (percentage points)	Urban(%)	Rural (%)	Diff (percentage points)
Kenya	2014	30.1(23.3, 36.9)	40.5(34.6, 46.4)	-10.4(-16.7, -4.1)	11.5(10.4, 12.6)	17.0(15.8, 18.1)	-5.5(-6.6, -4.4)
Kyrgyzstan	2012	100.0(100.0, 100.0)	54.2(32.7, 75.7)	45.8(27.6, 64.1)	34.3(30.1, 38.5)	36.1(33.3, 38.9)	-1.8(-5.1, 1.5)
Lao People's Democratic Republic	2011	83.3(40.5, 100.0)	69.2(50.2, 88.2)	14.1(-13.2, 41.4)	52.9(40.8, 65.1)	70.7(64.3, 77.1)	-17.8(-26.1, -9.5)
Lesotho	2014	21.7(9.4, 34.1)	44.1(36.7, 51.5)	-22.3(-31.4, -13.2)	15.6(12.7, 18.5)	24.1(21.8, 26.4)	-8.5(-11.0, -6.0)
Liberia	2013	71.7(66.6, 76.9)	83.5(79.1, 87.9)	-11.8(-16.5, -7.0)	53.9(50.8, 57.1)	61.3(58.7, 63.9)	-7.3(-10.2, -4.5)
Malawi	2015	35.2(27.5, 42.9)	36.2(32.9, 39.6)	-1.0(-5.5, 3.5)	20.9(18.9, 22.9)	24.9(23.8, 25.9)	-4.0(-5.3, -2.7)
Mali	2015	92.3(75.5, 100.0)	84.0(75.5, 92.5)	8.3(-3.6, 20.2)	69.9(62.0, 77.8)	84.8(82.0, 87.5)	-14.8(-19.9, -9.8)
Mauritania	2015	56.2(28.9, 83.6)	79.5(66.2, 92.7)	-23.2(-44.7, -1.8)	65.3(58.5, 72.1)	84.7(81.0, 88.4)	-19.4(-24.8, -13.9)
Mexico	2015	28.3(18.9, 37.6)	40.5(25.0, 56.0)	-12.2(-24.2, -0.3)	25.4(21.1, 29.6)	28.5(22.8, 34.1)	-3.1(-7.9, 1.7)
Moldova	2012	33.3(0.0, 100.0)	50.0(0.0, 100.0)	-16.7(-58.5, 25.2)	25.0(6.3, 43.7)	18.8(0.0, 40.2)	6.2(-12.5, 25.0)
Mongolia	2013	50.0(0.0, 100.0)	100.0(100.0, 100.0)	-50.0(-91.2, -8.8)	62.7(50.8, 74.6)	40.9(25.8, 56.0)	21.8(8.8, 34.8)
Mozambique	2011	57.2(52.2, 62.2)	82.7(78.4, 87.0)	-25.4(-30.1, -20.8)	43.5(40.8, 46.1)	71.2(68.6, 73.8)	-27.7(-30.3, -25.1)
Myanmar	2016	16.3(4.8, 27.8)	35.2(26.6, 43.8)	-19.0(-28.4, -9.6)	16.1(13.3, 18.9)	23.9(21.9, 25.8)	-7.7(-9.9, -5.6)
Namibia	2013	40.0(31.8, 48.3)	46.4(38.9, 53.8)	-6.4(-14.2, 1.5)	17.3(15.1, 19.4)	25.6(22.7, 28.5)	-8.3(-10.9, -5.8)
Nepal	2016	57.6(51.6, 63.7)	65.8(59.1, 72.6)	-8.2(-14.5, -1.9)	36.6(34.7, 38.4)	42.5(40.0, 45.1)	-5.9(-8.1, -3.8)
Niger	2012	44.6(31.2, 58.1)	67.6(60.5, 74.8)	-23.0(-31.9, -14.1)	33.1(29.6, 36.5)	57.5(54.9, 60.2)	-24.5(-27.4, -21.6)
Nigeria	2017	80.0(57.1, 100.0)	98.6(95.8, 100.0)	-18.6(-32.0, -5.2)	78.0(74.0, 82.1)	85.8(83.4, 88.1)	-7.8(-10.8, -4.8)
Pakistan	2012	51.2(40.1, 62.4)	65.5(55.1, 75.9)	-14.2(-24.9, -3.5)	34.8(32.6, 36.9)	49.3(47.1, 51.6)	-14.6(-16.8, -12.4)
Panama	2013	45.8(24.3, 67.3)	50.6(39.4, 61.9)	-4.8(-20.6, 11.0)	34.3(27.2, 41.3)	43.9(38.7, 49.1)	-9.6(-15.6, -3.7)
Paraguay	2016	25.0(6.3, 43.7)	9.1(0.0, 22.1)	15.9(0.7, 31.2)	16.7(11.0, 22.4)	21.7(12.6, 30.7)	-5.0(-12.4, 2.3)
Peru	2012	18.4(14.8, 21.9)	23.1(18.6, 27.6)	-4.7(-8.7, -0.7)	11.1(10.2, 12.1)	14.5(13.1, 15.9)	-3.3(-4.5, -2.2)

© 2020 Li Z et al. *JAMA Network Open.*

eTable 7. Prevalence of unmet need for family planning among adolescents 15-19 years old and adult women 20-34 years old in urban area, rural area, and absolute inequality between urban and rural areas (Diff), using the most recent years of data available (continued)

Country	Year	15-19			20-34		
		Urban(%)	Rural (%)	Diff (percentage points)	Urban(%)	Rural (%)	Diff (percentage points)
Philippines	2017	43.8(33.6, 53.9)	46.0(39.4, 52.6)	-2.2(-10.2, 5.7)	22.8(20.9, 24.8)	23.2(21.8, 24.6)	-0.4(-2.0, 1.2)
Rwanda	2015	62.9(46.0, 79.7)	57.1(43.8, 70.5)	5.7(-8.8, 20.3)	25.6(22.5, 28.7)	28.2(26.4, 30.0)	-2.6(-4.9, -0.3)
Sao Tome and Principe	2014	76.5(54.0, 99.0)	22.2(0.9, 43.5)	54.2(32.2, 76.3)	52.7(43.3, 62.1)	36.5(26.0, 46.9)	16.2(6.4, 26.0)
Senegal	2017	68.2(59.3, 77.2)	79.2(74.4, 84.0)	-11.0(-17.5, -4.4)	37.0(34.4, 39.7)	57.7(55.4, 60.0)	-20.7(-23.1, -18.3)
Sierra Leone	2013	69.7(65.7, 73.8)	82.1(78.2, 86.0)	-12.4(-16.4, -8.4)	39.0(36.7, 41.4)	61.2(58.8, 63.5)	-22.1(-24.5, -19.8)
Sudan	2014	57.1(7.7, 100.0)	84.6(69.8, 99.5)	-27.5(-58.4, 3.5)	68.3(62.1, 74.5)	83.6(79.8, 87.3)	-15.2(-19.9, -10.5)
Suriname	2010	62.5(19.2, 100.0)	63.2(39.3, 87.0)	-0.7(-34.5, 33.2)	40.6(22.6, 58.6)	64.5(55.5, 73.6)	-23.9(-37.7, -10.2)
Swaziland	2010	25.0(0.0, 100.0)	27.3(0.0, 58.7)	-2.3(-29.0, 24.4)	18.5(8.8, 28.2)	22.7(15.1, 30.3)	-4.2(-12.4, 4.0)
Tajikistan	2017	70.0(35.4, 100.0)	86.4(70.8, 100.0)	-16.4(-41.1, 8.3)	46.3(43.0, 49.6)	51.4(48.8, 53.9)	-5.1(-8.0, -2.2)
Tanzania	2015	59.3(49.4, 69.2)	59.7(53.9, 65.5)	-0.4(-7.5, 6.8)	26.6(23.9, 29.4)	41.7(39.6, 43.9)	-15.1(-17.4, -12.8)
Thailand	2012	20.8(8.9, 32.8)	28.6(14.3, 42.8)	-7.7(-20.8, 5.3)	13.3(8.5, 18.1)	12.1(7.4, 16.8)	1.2(-3.5, 6.0)
Timor-Leste	2016	60.0(36.5, 83.5)	75.0(64.1, 85.9)	-15.0(-29.7, -0.3)	50.8(47.2, 54.4)	49.7(47.2, 52.2)	1.1(-1.6, 3.9)
Togo	2014	54.7(44.5, 64.9)	74.2(68.4, 80.0)	-19.5(-26.9, -12.0)	53.9(50.6, 57.1)	64.5(61.9, 67.0)	-10.6(-13.4, -7.8)
Uganda	2016	42.3(32.7, 52.0)	62.0(57.9, 66.1)	-19.7(-25.4, -13.9)	33.1(30.4, 35.7)	45.1(43.5, 46.7)	-12.0(-13.9, -10.2)
Vietnam	2011	33.3(0.0, 100.0)	60.0(0.0, 100.0)	-26.7(-77.5, 24.1)	22.4(10.3, 34.6)	28.9(19.7, 38.0)	-6.4(-16.9, 4.1)
Yemen	2013	49.6(40.3, 58.8)	75.3(70.7, 79.9)	-25.7(-32.0, -19.5)	28.7(26.5, 30.8)	53.8(52.3, 55.4)	-25.1(-26.9, -23.4)
Zambia	2013	33.2(26.7, 39.6)	44.0(39.2, 48.8)	-10.9(-16.5, -5.2)	24.3(22.5, 26.1)	35.2(33.3, 37.2)	-10.9(-12.8, -9.0)
Zimbabwe	2015	24.4(13.1, 35.8)	18.0(12.2, 23.7)	6.5(-1.9, 14.8)	12.2(10.5, 14.0)	12.9(11.3, 14.5)	-0.7(-2.4, 1.0)

© 2020 Li Z et al. *JAMA Network Open.*

eTable 8. Estimates of contraception use and unmet need, using data from 43 countries with surveys available in all three rounds[1]

	15-19		20-34		Absolute inequality		Relative inequality	
	Value (%)	P value of change comparing to 2000-2006	Value (%)	P value of change comparing to 2000-2006	Value (percentage points)	P value of change comparing to 2000-2006	Value	P value of change comparing to 2000-2006
Modern contraceptive use								
2000-2006	17.8 (16.6, 19.0)	NA	30.9 (29.8, 32.0)	NA	13.2(12.4, 13.9)	NA	1.74(1.69, 1.79)	NA
2007-2012	22.7 (22.0, 23.4)	0.000	35.6 (34.8, 36.4)	0.000	13.0(12.5, 13.5)	0.449	1.57(1.53, 1.60)	0.886
2013-2017	27.2 (26.6, 27.8)	0.000	40.3 (39.8, 40.8)	0.000	13.0(12.6, 13.4)	0.469	1.48(1.45, 1.51)	0.743
Unmet need for family planning								
2000-2006	53.3 (52.1, 54.5)	NA	45.8 (44.9, 46.7)	NA	-7.5(-8.4, -6.5)	NA	0.86(0.82, 0.90)	NA
2007-2012	51.9 (50.9, 52.9)	0.059	42.5 (41.8, 43.2)	0.005	-9.5(-10.2, -8.8)	0.028	0.82(0.79, 0.85)	0.097
2013-2017	52.0 (51.1, 52.9)	0.144	38.0 (37.3, 38.7)	0.000	-14.0(-14.6, -13.4)	0.004	0.73(0.70, 0.76)	0.029

Note:

1. We kept 43 countries with at least one survey taken in each of the three rounds to ensure we studied the same cluster of countries in each survey round. The 43 countries were: Albania, Armenia, Bangladesh, Belize, Benin, Burundi, Cambodia, Cameroon, Chad, Colombia, Congo, Cuba, Côte d'Ivoire, Dominican Republic, Egypt, Ethiopia, Ghana, Guinea, Guinea-Bissau, Guyana, Haiti, Indonesia, Kazakhstan, Kenya, Kyrgyzstan, Lesotho, Malawi, Mali, Mongolia, Nepal, Nigeria, Philippines, Rwanda, Senegal, Serbia, Sierra Leone, Tajikistan, Tanzania, Thailand, Togo, Uganda, Vietnam, Zimbabwe.

© 2020 Li Z et al. *JAMA Network Open.*

eTable 9. Absolute inequality in contraception use and unmet need by FP2020 group, using data from 43 countries with surveys available in all three rounds[1]

	FP2020 group		Non-FP2020 group		P value between the FP2020 group and non-FP2020 group
	Value (percentage points)	P value of change comparing to 2000-2006	Value (percentage points)	P value of change comparing to 2000-2006	
Modern contraceptive use					
2000-2006	12.2 (11.3, 13.1)	NA	16.2 (14.7, 17.6)	NA	0.000
2007-2012	12.8 (12.2, 13.5)	0.433	13.3 (12.4, 14.1)	0.025	0.663
2013-2017	12.9 (12.5, 13.4)	0.295	13.3 (12.4, 14.1)	0.019	0.524
Unmet need for family planning					
2000-2006	-8.5 (-10.5, -6.5)	NA	-6.3 (-7.7, -4.9)	NA	0.219
2007-2012	-11.0 (-11.8, -10.2)	0.079	-8.2 (-10.1, -6.3)	0.315	0.042
2013-2017	-14.2 (-14.8, -13.7)	0.000	-12.3 (-13.1, -11.5)	0.000	0.012

Note:

1. We identified 33 FP2020 countries and 10 non-FP2020 countries. The FP2020 countries are Bangladesh, Benin, Burundi, Cambodia, Cameroon, Chad, Congo, Côte d'Ivoire, Egypt, Ethiopia, Ghana, Guinea, Guinea-Bissau, Haiti, Indonesia, Kenya, Kyrgyzstan, Lesotho, Malawi, Mali, Mongolia, Nepal, Nigeria, Philippines, Rwanda, Senegal, Sierra Leone, Tajikistan, Tanzania, Togo, Uganda, Vietnam, Zimbabwe. The non-FP2020 countries are Albania, Armenia, Belize, Colombia, Cuba, Dominican Republic, Guyana, Kazakhstan, Serbia, Thailand,

© 2020 Li Z et al. *JAMA Network Open.*

eTable 10. Modern contraceptive use among adolescents 15-19 years old and adult women 20-34 years old, and absolute inequality [1]

Country	Year	WHO region	Income classification[2]	FP2020 country	SSA[3]	15-19 (%)	20-34 (%)	Absolute inequality (percentage points) between 20-34 and 15-19
Afghanistan	2015	Eastern Mediterranean	L	Yes	0	6.0[4.4,7.6]	18.2[17.7,18.8]	12.2[11.4,13.0]
Afghanistan	2010	Eastern Mediterranean	L	Yes	0	5.9[4.5,7.3]	17.2[15.7,18.7]	11.3[9.8,12.8]
Albania	2017	Europe	UM	No	0	2.9[1.1,4.7]	4.0[3.5,4.5]	1.1[0.5,1.8]
Albania	2009	Europe	UM	No	0	11.0[6.0,16.0]	10.9[10.0,11.9]	-0.1[-1.5,1.2]
Albania	2005	Europe	LM	No	0	16.3[8.1,24.5]	24.6[23.5,25.7]	8.3[3.6,13.0]
Algeria	2012	Africa	UM	No	0	27.0[20.0,34.0]	45.6[44.4,46.8]	18.6[15.0,22.2]
Algeria	2006	Africa	LM	No	0	17.0[10.0,24.0]	48.8[47.6,50.0]	31.8[26.5,37.1]
Angola	2015	Africa	UM	No	1	7.6[5.0,10.2]	10.6[9.8,11.5]	3.0[1.5,4.5]
Argentina	2011	Americas	UM	No	0	32.8[29.7,35.9]	64.1[62.5,65.7]	31.3[29.2,33.4]
Armenia	2016	Europe	LM	No	0	19.7[15.1,24.4]	26.8[25.3,28.3]	7.1[5.1,9.0]
Armenia	2010	Europe	LM	No	0	3.1[0.4,5.8]	25.6[24.0,27.1]	22.5[20.8,24.2]
Armenia	2005	Europe	LM	No	0	4.5[1.1,7.9]	19.6[18.3,20.9]	15.1[13.5,16.7]
Azerbaijan	2006	Europe	LM	No	0	3.0[0.9,5.1]	14.1[13.1,15.2]	11.1[10.0,12.3]
Bangladesh	2014	South-East Asia	LM	Yes	0	46.7[44.2,49.2]	57.5[56.6,58.5]	10.8[9.3,12.4]
Bangladesh	2013	South-East Asia	L	Yes	0	45.7[43.9,47.5]	63.8[63.2,64.5]	18.1[17.1,19.2]
Bangladesh	2011	South-East Asia	L	Yes	0	42.4[39.7,45.1]	55.5[54.6,56.5]	13.1[11.5,14.7]
Bangladesh	2007	South-East Asia	L	Yes	0	37.6[34.0,41.2]	51.0[49.7,52.2]	13.4[11.2,15.5]
Bangladesh	2004	South-East Asia	L	Yes	0	34.1[31.8,36.4]	52.1[50.9,53.4]	18.0[16.4,19.6]
Belarus	2012	Europe	UM	No	0	48.4[27.8,69.0]	51.9[49.7,54.2]	3.5[-3.6,10.6]
Belarus	2005	Europe	LM	No	0	50.7[32.3,69.1]	63.2[61.0,65.4]	12.5[5.1,19.9]
Belize	2016	Americas	UM	No	0	35.7[27.3,44.0]	52.4[49.9,54.9]	16.7[11.9,21.6]
Belize	2011	Americas	LM	No	0	33.8[26.8,40.8]	51.9[49.1,54.7]	18.1[13.6,22.6]
Belize	2006	Americas	UM	No	0	11.0[7.8,14.2]	37.6[34.8,40.4]	26.6[23.7,29.5]
Benin	2014	Africa	L	Yes	1	7.4[5.0,9.7]	11.3[10.5,12.0]	3.9[2.5,5.3]
Benin	2012	Africa	L	Yes	1	6.0[4.7,7.3]	7.4[6.8,7.9]	1.3[0.6,2.1]
Benin	2006	Africa	L	Yes	1	2.9[1.8,4.0]	6.6[6.1,7.1]	3.7[3.0,4.3]

Note:

1. We mark the countries with absolute inequality (difference) between mothers 20-34 and 15-19 years old larger than 5% in orange.
2. Income classification: "L" represents "low-income country"; "LM" represents "lower-middle-income country"; "UM" represents "upper-middle-income country".
3. SSA: "1" represents "Sub-Saharan African country"; "0" represents "Not a Sub-Saharan African country".

© 2020 Li Z et al. *JAMA Network Open.*

eTable 10. Modern contraceptive use among adolescents 15-19 years old and adult women 20-34 years old, and absolute inequality[1] (continued)

Country	Year	WHO region	Income classification[2]	FP2020 country	SSA[3]	15-19 (%)	20-34 (%)	Absolute inequality (percentage points) between 20-34 and 15-19
Bhutan	2010	South-East Asia	LM	Yes	0	30.2[24.5,35.9]	64.4[63.1,65.7]	34.2[31.3,37.1]
Bolivia	2008	Americas	LM	Yes	0	26.6[22.4,30.8]	40.0[38.9,41.2]	13.4[11.4,15.5]
Bolivia	2003	Americas	LM	Yes	0	26.3[21.6,31.0]	38.3[37.1,39.4]	12.0[9.8,14.1]
Bolivia	2000	Americas	LM	Yes	0	0.7[0.0,2.2]	2.0[1.4,2.7]	1.3[0.3,2.3]
Bosnia and Herzegovina	2011	Europe	UM	No	0	12.5[0.0,42.1]	12.0[10.8,13.2]	-0.5[-12.1,11.1]
Bosnia and Herzegovina	2006	Europe	LM	No	0	11.5[3.5,19.5]	10.8[9.6,12.0]	-0.7[-3.8,2.4]
Burkina Faso	2010	Africa	L	Yes	1	6.2[4.4,8.0]	17.8[16.9,18.6]	11.6[10.5,12.6]
Burkina Faso	2006	Africa	L	Yes	1	6.0[3.5,8.5]	15.3[13.7,16.9]	9.3[7.4,11.2]
Burkina Faso	2003	Africa	L	Yes	1	4.4[3.1,5.7]	10.8[10.0,11.6]	6.4[5.5,7.3]
Burundi	2016	Africa	L	Yes	1	29.0[25.5,32.5]	24.5[23.5,25.6]	-4.5[-6.1,-3.0]
Burundi	2010	Africa	L	Yes	1	8.1[4.0,12.2]	21.5[20.1,22.9]	13.4[11.4,15.4]
Burundi	2005	Africa	L	Yes	1	2.6[1.0,4.2]	8.2[7.0,9.4]	5.6[4.3,6.9]
Cambodia	2014	Western Pacific	L	Yes	0	20.2[16.1,24.3]	43.5[42.4,44.5]	23.3[21.6,24.9]
Cambodia	2010	Western Pacific	L	Yes	0	18.8[14.2,23.4]	38.8[37.8,39.9]	20.0[18.3,21.8]
Cambodia	2005	Western Pacific	L	Yes	0	13.7[10.1,17.3]	30.1[29.0,31.2]	16.4[14.8,18.0]
Cameroon	2014	Africa	LM	Yes	1	9.3[6.6,12.0]	22.5[21.6,23.4]	13.2[11.5,14.9]
Cameroon	2011	Africa	LM	Yes	1	12.2[9.6,14.8]	16.3[15.4,17.2]	4.1[2.6,5.6]
Cameroon	2006	Africa	LM	Yes	1	6.5[4.3,8.7]	12.2[11.3,13.1]	5.7[4.3,7.1]
Cameroon	2004	Africa	L	Yes	1	14.8[12.0,17.6]	13.3[12.3,14.3]	-1.5[-3.2,0.2]
Central African Republic	2010	Africa	L	Yes	1	6.7[4.7,8.7]	11.9[10.9,12.9]	5.2[3.8,6.6]
Central African Republic	2006	Africa	L	Yes	1	9.3[7.0,11.6]	9.7[8.7,10.7]	0.4[-1.1,1.9]
Central African Republic	2000	Africa	L	Yes	1	0.1[0.0,0.2]	0.2[0.1,0.4]	0.2[0.1,0.3]
Chad	2015	Africa	L	Yes	1	2.4[1.8,3.0]	3.6[3.2,4.0]	1.2[0.8,1.7]
Chad	2010	Africa	L	Yes	1	0.9[0.5,1.3]	1.8[1.4,2.2]	0.9[0.5,1.3]
Chad	2004	Africa	L	Yes	1	1.4[0.8,2.0]	2.9[2.2,3.5]	1.5[0.9,2.1]
Chad	2000	Africa	L	Yes	1	0.7[0.0,1.4]	0.6[0.3,1.0]	-0.0[-0.5,0.5]
Colombia	2015	Americas	UM	No	0	60.5[56.3,64.7]	74.2[73.5,74.9]	13.7[11.7,15.7]
Colombia	2010	Americas	UM	No	0	55.1[51.7,58.5]	75.8[75.2,76.4]	20.7[19.1,22.3]
Colombia	2005	Americas	LM	No	0	47.1[43.0,51.2]	71.1[70.4,71.8]	24.0[22.1,25.9]

© 2020 Li Z et al. *JAMA Network Open.*

eTable 10. Modern contraceptive use among adolescents 15-19 years old and adult women 20-34 years old, and absolute inequality[1] (continued)

Country	Year	WHO region	Income classification[2]	FP2020 country	SSA[3]	15-19 (%)	20-34 (%)	Absolute inequality (percentage points) between 20-34 and 15-19
Comoros	2012	Africa	L	Yes	1	13.5[8.9,18.1]	14.5[12.9,16.1]	1.0[-1.4,3.3]
Congo	2015	Africa	LM	Yes	1	16.7[13.1,20.2]	18.7[17.4,20.0]	2.0[-0.1,4.2]
Congo	2011	Africa	LM	Yes	1	26.3[20.9,31.7]	16.7[15.6,17.7]	-9.6[-12.4,-6.8]
Congo	2005	Africa	LM	Yes	1	11.1[7.2,15.0]	15.8[14.4,17.1]	4.7[2.4,7.0]
Costa Rica	2011	Americas	UM	No	0	64.1[48.9,79.3]	73.5[70.0,77.0]	9.4[1.3,17.5]
Cuba	2014	Americas	UM	No	0	59.9[44.6,75.2]	70.8[68.4,73.2]	10.9[4.8,17.0]
Cuba	2010	Americas	UM	No	0	67.0[52.0,82.0]	74.5[72.1,76.9]	7.5[1.2,13.8]
Cuba	2006	Americas	LM	No	0	54.8[50.7,58.9]	76.6[74.2,79.0]	21.8[19.0,24.6]
Côte d'Ivoire	2016	Africa	LM	Yes	1	5.4[3.3,7.4]	12.9[11.9,13.9]	7.6[6.2,8.9]
Côte d'Ivoire	2012	Africa	LM	Yes	1	12.8[10.8,14.9]	14.0[13.0,15.1]	1.2[-0.1,2.5]
Côte d'Ivoire	2006	Africa	L	Yes	1	12.5[8.4,16.6]	8.8[7.6,10.0]	-3.7[-6.2,-1.2]
Democratic Republic of the Congo	2013	Africa	L	Yes	1	5.4[3.8,7.0]	6.9[6.3,7.4]	1.5[0.6,2.4]
Democratic Republic of the Congo	2010	Africa	L	Yes	1	3.7[2.2,5.2]	5.9[5.1,6.7]	2.2[1.2,3.2]
Democratic Republic of the Congo	2007	Africa	L	Yes	1	4.4[2.6,6.2]	7.2[6.4,8.0]	2.8[1.7,3.8]
Djibouti	2006	Africa	LM	Yes	1	16.3[6.2,26.4]	20.2[17.9,22.5]	3.9[-1.8,9.6]
Dominican Republic	2014	Americas	UM	No	0	51.2[47.0,55.4]	63.8[62.3,65.3]	12.6[10.1,15.1]
Dominican Republic	2013	Americas	UM	No	0	51.7[45.1,58.3]	75.8[74.5,77.1]	24.1[21.0,27.3]
Dominican Republic	2007	Americas	LM	No	0	43.8[39.5,48.1]	78.2[77.4,78.9]	34.4[32.3,36.4]
Dominican Republic	2000	Americas	LM	No	0	1.2[0.0,2.9]	31.9[29.3,34.6]	30.7[28.3,33.1]
Egypt	2014	Eastern Mediterranean	LM	Yes	0	18.9[15.7,22.1]	53.5[52.7,54.2]	34.6[33.4,35.7]
Egypt	2008	Eastern Mediterranean	LM	Yes	0	19.8[16.5,23.1]	63.5[62.6,64.4]	43.7[42.4,45.0]
Egypt	2005	Eastern Mediterranean	LM	Yes	0	24.1[19.0,29.2]	62.2[61.4,63.0]	38.1[36.4,39.8]
El Salvador	2014	Americas	LM	No	0	58.3[52.7,63.9]	66.6[65.0,68.2]	8.3[5.1,11.5]
Ethiopia	2016	Africa	L	Yes	1	32.3[29.6,35.0]	31.3[30.2,32.5]	-1.0[-2.5,0.5]
Ethiopia	2011	Africa	L	Yes	1	23.0[18.4,27.6]	26.4[25.3,27.4]	3.4[1.2,5.6]
Ethiopia	2005	Africa	L	Yes	1	8.6[6.2,11.0]	17.3[16.3,18.3]	8.7[7.3,10.1]
Gabon	2012	Africa	UM	No	1	15.6[10.2,21.0]	17.9[16.6,19.3]	2.3[-0.8,5.5]

© 2020 Li Z et al. *JAMA Network Open.*

eTable 10. Modern contraceptive use among adolescents 15-19 years old and adult women 20-34 years old, and absolute inequality[1] (continued)

Country	Year	WHO region	Income classification[2]	FP2020 country	SSA[3]	15-19 (%)	20-34 (%)	Absolute inequality (percentage points) between 20-34 and 15-19
Gambia	2013	Africa	L	Yes	1	2.2[1.0,3.4]	9.4[8.5,10.3]	7.2[6.2,8.1]
Gambia	2010	Africa	L	Yes	1	2.4[2.0,2.8]	8.1[7.6,8.6]	5.7[5.3,6.1]
Georgia	2005	Europe	LM	No	0	8.6[4.9,12.3]	25.6[24.0,27.2]	17.0[14.7,19.3]
Ghana	2014	Africa	LM	Yes	1	16.7[10.0,23.4]	24.2[22.8,25.6]	7.5[4.7,10.3]
Ghana	2011	Africa	LM	Yes	1	12.1[6.8,17.4]	26.0[24.4,27.6]	13.9[10.7,17.1]
Ghana	2008	Africa	L	Yes	1	7.6[3.5,11.7]	18.1[16.4,19.8]	10.5[8.3,12.7]
Ghana	2006	Africa	L	Yes	1	6.0[2.6,9.4]	13.1[11.5,14.7]	7.1[4.8,9.4]
Ghana	2003	Africa	L	Yes	1	6.9[3.7,10.1]	19.2[17.6,20.8]	12.3[10.3,14.2]
Guatemala	2015	Americas	LM	No	0	44.9[42.8,47.0]	54.3[53.4,55.3]	9.4[8.2,10.7]
Guinea	2016	Africa	L	Yes	1	5.7[4.3,7.1]	8.3[7.6,9.0]	2.6[1.6,3.6]
Guinea	2012	Africa	L	Yes	1	2.6[1.5,3.7]	4.6[4.0,5.3]	2.0[1.3,2.8]
Guinea	2005	Africa	L	Yes	1	5.0[3.2,6.8]	3.8[3.2,4.4]	-1.2[-2.2,-0.2]
Guinea-Bissau	2014	Africa	L	Yes	1	4.1[1.9,6.3]	14.2[13.4,15.0]	10.1[8.7,11.5]
Guinea-Bissau	2010	Africa	L	Yes	1	4.5[3.0,6.0]	11.2[10.4,12.0]	6.7[5.6,7.8]
Guinea-Bissau	2006	Africa	L	Yes	1	5.1[3.3,6.9]	6.0[5.2,6.8]	0.9[-0.3,2.1]
Guinea-Bissau	2000	Africa	L	Yes	1	0.2[0.0,0.6]	0.1[0.0,0.3]	-0.1[-0.3,0.2]
Guyana	2014	Americas	LM	No	0	12.4[8.7,16.1]	37.7[35.8,39.6]	25.3[22.8,27.8]
Guyana	2009	Americas	LM	No	0	29.8[20.9,38.7]	43.8[41.7,46.0]	14.0[9.8,18.3]
Guyana	2006	Americas	LM	No	0	19.7[13.2,26.2]	38.4[36.5,40.3]	18.7[14.8,22.6]
Haiti	2017	Americas	L	Yes	0	34.2[31.1,37.2]	35.6[34.2,36.9]	1.4[-0.4,3.1]
Haiti	2012	Americas	L	Yes	0	24.0[18.8,29.2]	35.0[33.6,36.3]	11.0[8.4,13.5]
Haiti	2006	Americas	L	Yes	0	25.9[22.7,29.0]	26.4[24.9,27.8]	0.5[-1.3,2.4]
Honduras	2012	Americas	LM	Yes	0	59.7[57.6,61.7]	68.9[68.0,69.9]	9.3[8.0,10.5]
Honduras	2006	Americas	LM	Yes	0	51.9[49.6,54.1]	59.1[58.0,60.2]	7.2[5.8,8.6]
India	2015	South-East Asia	LM	Yes	0	43.3[42.9,43.8]	50.0[49.9,50.2]	6.7[6.5,6.9]
India	2006	South-East Asia	L	Yes	0	44.3[43.4,45.3]	57.1[56.8,57.5]	12.8[12.3,13.3]
Indonesia	2017	South-East Asia	LM	Yes	0	47.6[42.7,52.5]	60.6[59.9,61.2]	13.0[11.2,14.8]

© 2020 Li Z et al. *JAMA Network Open.*

eTable 10. Modern contraceptive use among adolescents 15-19 years old and adult women 20-34 years old, and absolute inequality[1] (continued)

Country	Year	WHO region	Income classification[2]	FP2020 country	SSA[3]	15-19 (%)	20-34 (%)	Absolute inequality (percentage points) between 20-34 and 15-19
Indonesia	2007	South-East Asia	LM	Yes	0	46.2[41.0,51.4]	60.0[59.3,60.6]	13.8[11.8,15.7]
Indonesia	2002	South-East Asia	L	Yes	0	46.8[41.5,52.1]	60.8[60.1,61.5]	14.0[11.9,16.1]
Iraq	2011	Eastern Mediterranean	LM	Yes	0	11.1[9.4,12.8]	31.5[30.8,32.2]	20.4[19.3,21.5]
Iraq	2006	Eastern Mediterranean	LM	Yes	0	10.0[8.1,11.9]	30.5[29.8,31.2]	20.5[19.3,21.7]
Jordan	2012	Eastern Mediterranean	UM	No	0	21.8[14.0,29.6]	43.3[42.2,44.5]	21.5[19.5,23.5]
Jordan	2007	Eastern Mediterranean	LM	No	0	15.1[8.8,21.4]	41.0[39.9,42.2]	25.9[24.1,27.7]
Kazakhstan	2015	Europe	UM	No	0	25.5[16.6,34.4]	54.0[52.5,55.5]	28.5[24.5,32.5]
Kazakhstan	2010	Europe	UM	No	0	18.4[12.0,24.8]	49.6[48.1,51.1]	31.2[27.8,34.6]
Kazakhstan	2006	Europe	UM	No	0	28.5[20.1,36.9]	50.3[48.8,51.8]	21.8[17.0,26.6]
Kenya	2014	Africa	LM	Yes	1	36.8[32.2,41.4]	52.5[51.7,53.4]	15.7[13.7,17.7]
Kenya	2009	Africa	L	Yes	1	28.3[24.9,31.7]	41.1[39.5,42.7]	12.7[10.7,14.8]
Kenya	2003	Africa	L	Yes	1	12.7[9.4,16.0]	37.7[36.1,39.4]	25.0[23.0,27.1]
Kosovo	2014	Europe	LM	No	0	4.0[0.0,12.3]	11.4[9.7,13.1]	7.4[2.7,12.1]
Kyrgyzstan	2014	Europe	LM	Yes	0	9.7[5.1,14.3]	38.4[36.2,40.6]	28.7[25.7,31.7]
Kyrgyzstan	2012	Europe	L	Yes	0	5.2[2.7,7.7]	39.6[38.1,41.1]	34.4[32.8,36.0]
Kyrgyzstan	2005	Europe	L	Yes	0	14.7[7.6,21.8]	45.1[42.9,47.3]	30.4[25.9,34.9]
Lao People's Democratic Republic	2017	Western Pacific	LM	Yes	0	29.1[26.3,31.9]	50.6[49.3,51.9]	21.5[19.8,23.2]
Lao People's Democratic Republic	2011	Western Pacific	LM	Yes	0	22.3[19.5,25.1]	44.9[43.6,46.2]	22.6[20.7,24.5]
Lebanon	2011	Eastern Mediterranean	UM	No	0	26.1[15.5,36.7]	49.6[46.9,52.4]	23.5[17.1,29.9]
Lebanon	2005	Eastern Mediterranean	UM	No	0	21.3[10.7,31.9]	53.2[50.4,56.0]	31.9[28.1,35.7]
Lesotho	2014	Africa	LM	Yes	1	35.3[28.6,42.0]	67.3[65.5,69.1]	32.0[28.5,35.5]
Lesotho	2009	Africa	LM	Yes	1	26.8[21.7,31.9]	50.4[48.6,52.3]	23.6[20.7,26.6]
Lesotho	2004	Africa	L	Yes	1	14.7[10.8,18.6]	41.2[39.3,43.2]	26.5[24.0,29.0]
Liberia	2013	Africa	L	Yes	1	13.2[8.3,18.1]	21.8[20.5,23.1]	8.6[5.9,11.2]
Liberia	2007	Africa	L	Yes	1	4.4[2.1,6.7]	13.2[12.0,14.4]	8.8[7.3,10.4]
Madagascar	2009	Africa	L	Yes	1	25.3[23.5,27.2]	33.7[32.6,34.8]	8.4[7.0,9.7]
Madagascar	2004	Africa	L	Yes	1	18.7[15.9,21.5]	25.4[23.9,26.9]	6.7[4.9,8.5]

© 2020 Li Z et al. *JAMA Network Open.*

eTable 10. Modern contraceptive use among adolescents 15-19 years old and adult women 20-34 years old, and absolute inequality[1] (continued)

Country	Year	WHO region	Income classification[2]	FP2020 country	SSA[3]	15-19 (%)	20-34 (%)	Absolute inequality (percentage points) between 20-34 and 15-19
Malawi	2015	Africa	L	Yes	1	37.5[34.0,41.0]	63.2[62.3,64.1]	25.7[23.9,27.5]
Malawi	2013	Africa	L	Yes	1	39.2[35.2,43.2]	60.1[58.8,61.4]	20.9[18.5,23.3]
Malawi	2010	Africa	L	Yes	1	26.4[23.2,29.6]	46.7[45.8,47.7]	20.3[18.6,22.0]
Malawi	2006	Africa	L	Yes	1	22.7[20.1,25.3]	41.0[39.7,42.3]	18.3[16.6,20.0]
Malawi	2004	Africa	L	Yes	1	16.6[13.8,19.4]	30.8[29.5,32.1]	14.2[12.5,15.9]
Maldives	2009	South-East Asia	LM	No	0	9.6[4.7,14.5]	30.2[28.9,31.6]	20.6[18.8,22.5]
Mali	2015	Africa	L	Yes	1	7.7[6.4,9.1]	15.6[14.8,16.4]	7.9[6.9,8.9]
Mali	2012	Africa	L	Yes	1	6.5[4.8,8.2]	11.2[10.3,12.0]	4.7[3.6,5.8]
Mali	2009	Africa	L	Yes	1	5.4[4.3,6.5]	8.9[8.1,9.7]	3.5[2.6,4.4]
Mali	2006	Africa	L	Yes	1	5.8[4.5,7.1]	7.3[6.6,7.9]	1.5[0.6,2.3]
Mauritania	2015	Africa	LM	Yes	1	8.2[6.1,10.2]	18.5[17.4,19.7]	10.4[8.9,11.9]
Mauritania	2011	Africa	L	Yes	1	6.9[5.0,8.8]	12.6[11.7,13.5]	5.7[4.4,7.0]
Mauritania	2007	Africa	L	Yes	1	55.0[53.7,56.3]	73.7[72.8,74.6]	18.7[17.7,19.9]
Mexico	2015	Americas	UM	No	0	50.4[42.9,57.9]	64.5[61.6,67.4]	14.1[9.7,18.5]
Moldova	2012	Europe	LM	No	0	35.8[25.6,46.0]	42.9[41.7,44.1]	7.1[2.0,12.2]
Moldova	2005	Europe	LM	No	0	33.9[25.6,42.2]	44.2[42.6,45.8]	10.3[7.1,13.5]
Mongolia	2013	Western Pacific	LM	Yes	0	27.6[18.6,36.6]	42.1[40.8,43.4]	14.5[10.2,18.8]
Mongolia	2010	Western Pacific	LM	Yes	0	51.7[37.0,66.4]	52.9[51.6,54.2]	1.2[-5.9,8.3]
Mongolia	2005	Western Pacific	L	Yes	0	59.9[44.9,74.9]	64.1[62.8,65.4]	4.2[-3.5,11.9]
Morocco	2003	Eastern Mediterranean	LM	No	0	35.9[30.6,41.2]	57.9[56.6,59.1]	22.0[19.9,24.0]
Mozambique	2011	Africa	L	Yes	1	5.8[4.4,7.2]	18.2[17.2,19.2]	12.4[11.3,13.6]
Mozambique	2008	Africa	L	Yes	1	7.8[6.1,9.5]	13.8[12.6,15.0]	6.0[4.6,7.4]
Mozambique	2003	Africa	L	Yes	1	15.8[13.2,18.4]	17.3[16.2,18.3]	1.5[-0.2,3.1]
Myanmar	2016	South-East Asia	LM	Yes	0	54.5[49.8,59.1]	53.4[52.1,54.8]	-1.0[-2.9,0.8]
Myanmar	2009	South-East Asia	L	Yes	0	48.6[42.5,54.7]	53.8[52.3,55.3]	5.2[2.1,8.3]
Namibia	2013	Africa	UM	No	1	32.2[22.5,41.9]	62.8[61.0,64.6]	30.6[26.0,35.1]
Namibia	2007	Africa	LM	No	1	55.9[51.9,59.9]	59.9[58.1,61.7]	4.0[1.6,6.4]
Nepal	2016	South-East Asia	L	Yes	0	34.5[32.1,37.0]	49.6[48.4,50.7]	15.1[13.6,16.5]

© 2020 Li Z et al. *JAMA Network Open.*

eTable 10. Modern contraceptive use among adolescents 15-19 years old and adult women 20-34 years old, and absolute inequality[1] (continued)

Country	Year	WHO region	Income classification[2]	FP2020 country	SSA[3]	15-19 (%)	20-34 (%)	Absolute inequality (percentage points) between 20-34 and 15-19
Nepal	2014	South-East Asia	L	Yes	0	16.6[13.4,19.8]	41.9[39.5,44.3]	25.3[22.6,28.0]
Nepal	2011	South-East Asia	L	Yes	0	14.4[11.3,17.5]	50.8[49.6,52.0]	36.4[34.8,38.1]
Nepal	2006	South-East Asia	L	Yes	0	13.8[11.3,16.3]	50.9[49.6,52.2]	37.1[35.5,38.7]
Niger	2012	Africa	L	Yes	1	5.9[4.4,7.4]	12.4[11.5,13.2]	6.5[5.5,7.5]
Niger	2006	Africa	L	Yes	1	1.7[0.9,2.5]	8.3[7.5,9.1]	6.6[5.8,7.4]
Nigeria	2017	Africa	LM	Yes	1	1.4[0.8,2.1]	9.4[8.9,9.9]	8.0[7.4,8.5]
Nigeria	2013	Africa	LM	Yes	1	1.2[0.7,1.7]	11.1[10.7,11.6]	9.9[9.4,10.4]
Nigeria	2011	Africa	LM	Yes	1	2.2[1.4,3.0]	8.8[8.0,9.6]	6.6[5.8,7.4]
Nigeria	2008	Africa	LM	Yes	1	2.4[1.7,3.1]	8.9[8.4,9.4]	6.5[6.0,7.0]
Nigeria	2007	Africa	L	Yes	1	2.0[1.1,2.9]	8.0[7.2,8.8]	6.0[5.2,6.8]
Nigeria	2003	Africa	L	Yes	1	3.8[1.8,5.8]	8.5[7.5,9.5]	4.7[3.5,6.0]
Pakistan	2012	Eastern Mediterranean	LM	Yes	0	6.9[4.5,9.3]	31.0[30.0,31.9]	24.1[22.9,25.2]
Pakistan	2006	Eastern Mediterranean	L	Yes	0	4.2[2.7,5.7]	25.9[24.8,27.0]	21.7[20.6,22.8]
Panama	2013	Americas	UM	No	0	27.9[21.3,34.5]	54.6[52.9,56.4]	26.7[22.9,30.6]
Paraguay	2016	Americas	UM	No	0	65.6[59.9,71.2]	74.0[72.4,75.7]	8.5[5.3,11.7]
Peru	2012	Americas	UM	No	0	50.6[45.4,55.8]	53.7[52.7,54.6]	3.1[0.7,5.4]
Peru	2011	Americas	UM	No	0	44.4[39.0,49.8]	53.0[52.0,54.0]	8.6[6.3,10.9]
Peru	2010	Americas	UM	No	0	44.6[39.0,50.2]	53.0[52.0,54.0]	8.4[6.0,10.8]
Peru	2009	Americas	UM	No	0	40.9[35.3,46.5]	53.1[52.2,54.1]	12.2[9.8,14.7]
Peru	2008	Americas	UM	No	0	44.5[37.3,51.7]	51.7[51.0,52.5]	7.2[4.3,10.2]
Peru	2006	Americas	LM	No	0	47.8[35.3,60.3]	51.7[51.0,52.5]	3.9[-1.1,9.0]
Philippines	2017	Western Pacific	LM	Yes	0	44.5[41.8,47.1]	45.2[44.2,46.1]	0.7[-0.6,2.0]
Philippines	2013	Western Pacific	LM	Yes	0	20.6[16.5,24.7]	42.0[40.8,43.2]	21.4[19.6,23.2]
Philippines	2008	Western Pacific	LM	Yes	0	14.3[10.5,18.1]	39.7[38.5,41.0]	25.4[23.7,27.1]
Philippines	2003	Western Pacific	LM	Yes	0	13.2[9.2,17.2]	38.8[37.6,40.0]	25.6[23.9,27.3]
Rwanda	2015	Africa	L	Yes	1	46.5[41.0,51.9]	52.3[50.9,53.6]	5.8[3.7,7.9]
Rwanda	2010	Africa	L	Yes	1	30.6[21.5,39.7]	50.6[49.2,52.0]	20.0[17.1,23.0]

© 2020 Li Z et al. *JAMA Network Open.*

eTable 10. Modern contraceptive use among adolescents 15-19 years old and adult women 20-34 years old, and absolute inequality[1] (continued)

Country	Year	WHO region	Income classification[2]	FP2020 country	SSA[3]	15-19 (%)	20-34 (%)	Absolute inequality (percentage points) between 20-34 and 15-19
Rwanda	2005	Africa	L	Yes	1	3.2[0.9,5.5]	10.9[9.9,11.9]	7.7[6.5,8.8]
Sao Tome and Principe	2014	Africa	LM	Yes	1	27.6[18.4,36.8]	40.1[38.0,42.2]	12.5[7.0,18.0]
Sao Tome and Principe	2008	Africa	LM	Yes	1	21.4[14.3,28.5]	39.5[36.6,42.3]	18.1[14.1,22.1]
Senegal	2017	Africa	L	Yes	1	17.0[15.0,19.1]	26.6[25.6,27.7]	9.6[8.3,10.9]
Senegal	2016	Africa	L	Yes	1	11.6[9.2,13.9]	23.7[22.3,25.1]	12.1[10.5,13.7]
Senegal	2015	Africa	L	Yes	1	5.5[3.7,7.3]	20.9[19.6,22.3]	15.4[14.0,16.9]
Senegal	2014	Africa	LM	Yes	1	12.3[5.8,18.8]	18.1[17.2,19.1]	5.8[3.0,8.7]
Senegal	2013	Africa	LM	Yes	1	8.2[6.1,10.2]	16.1[14.9,17.3]	7.9[6.5,9.3]
Senegal	2011	Africa	LM	Yes	1	5.5[4.3,6.7]	11.9[11.1,12.7]	6.4[5.5,7.2]
Senegal	2005	Africa	L	Yes	1	4.7[3.1,6.3]	10.1[9.4,10.9]	5.4[4.5,6.4]
Serbia	2014	Europe	UM	No	0	1.6[0.2,3.0]	7.0[5.2,8.8]	5.4[3.6,7.2]
Serbia	2010	Europe	UM	No	0	10.8[1.8,19.8]	23.9[22.1,25.7]	13.1[9.5,16.7]
Serbia	2005	Europe	UM	No	0	6.3[1.8,10.8]	17.1[15.3,18.9]	10.8[8.2,13.4]
Sierra Leone	2017	Africa	L	Yes	1	14.0[13.0,15.0]	20.1[19.3,20.9]	6.1[5.2,7.0]
Sierra Leone	2013	Africa	L	Yes	1	7.8[5.8,9.8]	18.7[17.8,19.6]	10.9[9.7,12.2]
Sierra Leone	2010	Africa	L	Yes	1	4.6[3.1,6.1]	9.3[8.5,10.1]	4.7[3.6,5.8]
Sierra Leone	2008	Africa	L	Yes	1	1.2[0.3,2.1]	9.3[8.3,10.2]	8.1[7.2,9.0]
Sierra Leone	2005	Africa	L	Yes	1	0.7[0.2,1.2]	5.0[4.2,5.8]	4.3[3.5,5.1]
South Sudan	2010	Africa	L	Yes	1	1.0[0.3,1.7]	1.3[1.0,1.6]	0.3[-0.1,0.7]
Sudan	2014	Africa	LM	Yes	1	5.8[4.0,7.6]	12.2[11.2,13.2]	6.4[5.1,7.7]
Sudan	2010	Africa	LM	Yes	1	4.1[2.7,5.5]	8.2[7.2,9.2]	4.1[3.0,5.2]
Suriname	2010	Americas	UM	No	0	42.1[32.7,51.5]	44.0[42.0,46.0]	1.9[-3.4,7.2]
Suriname	2006	Americas	LM	No	0	37.7[28.0,47.4]	45.0[43.0,47.0]	7.3[1.9,12.7]
Swaziland	2014	Africa	LM	No	1	48.8[32.8,64.8]	71.7[68.9,74.5]	23.0[13.5,32.4]
Swaziland	2010	Africa	LM	No	1	54.5[39.2,69.9]	68.9[66.1,71.8]	14.4[5.5,23.3]
Syrian Arab Republic	2006	Eastern Mediterranean	LM	No	0	11.2[8.7,13.7]	38.5[37.3,39.7]	27.3[25.6,29.0]

© 2020 Li Z et al. *JAMA Network Open.*

eTable 10. Modern contraceptive use among adolescents 15-19 years old and adult women 20-34 years old, and absolute inequality[1] (continued)

Country	Year	WHO region	Income classification[2]	FP2020 country	SSA[3]	15-19 (%)	20-34 (%)	Absolute inequality (percentage points) between 20-34 and 15-19
Tajikistan	2017	Europe	Yes	Yes	0	10.7[7.5,13.8]	30.7[29.5,31.9]	20.0[18.6,21.4]
Tajikistan	2012	Europe	Yes	Yes	0	1.8[0.7,2.9]	31.5[30.2,32.9]	29.7[28.4,31.0]
Tajikistan	2005	Europe	Yes	Yes	0	3.9[1.7,6.1]	28.5[26.9,30.1]	24.6[22.8,26.4]
Tanzania	2015	Africa	Yes	Yes	1	13.3[10.5,16.1]	34.5[33.2,35.7]	21.2[19.5,22.9]
Tanzania	2010	Africa	Yes	Yes	1	12.0[8.2,15.8]	28.5[27.1,29.8]	16.5[14.4,18.5]
Tanzania	2004	Africa	Yes	Yes	1	6.9[4.9,8.9]	22.9[21.6,24.1]	16.0[14.6,17.4]
Thailand	2015	South-East Asia	No	No	0	68.2[63.2,73.2]	73.3[72.0,74.6]	5.1[2.7,7.5]
Thailand	2012	South-East Asia	No	No	0	68.3[61.8,74.8]	73.1[71.8,74.4]	4.8[1.5,8.1]
Thailand	2006	South-East Asia	No	No	0	68.6[65.1,72.0]	74.5[73.6,75.4]	5.9[4.1,7.8]
Thailand	2005	South-East Asia	No	No	0	66.6[61.7,71.5]	70.9[69.6,72.2]	4.3[1.3,7.3]
The former Yugoslav Republic of Macedonia	2011	Europe	No	No	0	8.2[1.8,14.6]	13.0[11.2,14.9]	4.8[1.4,8.3]
The former Yugoslav Republic of Macedonia	2005	Europe	No	No	0	1.2[0.3,2.1]	10.5[9.5,11.6]	9.3[8.3,10.4]
Timor-Leste	2016	South-East Asia	Yes	Yes	0	20.3[16.4,24.2]	27.6[26.4,28.8]	7.3[5.7,8.9]
Timor-Leste	2009	South-East Asia	Yes	Yes	0	6.8[4.2,9.4]	23.8[22.6,25.0]	17.0[15.6,18.4]
Togo	2014	Africa	Yes	Yes	1	14.5[11.7,17.2]	19.5[18.3,20.6]	5.0[3.5,6.5]
Togo	2010	Africa	Yes	Yes	1	4.3[2.2,6.4]	13.3[12.1,14.5]	9.0[7.5,10.5]
Togo	2006	Africa	Yes	Yes	1	11.7[7.5,15.9]	12.0[10.8,13.2]	0.3[-2.1,2.7]
Tunisia	2012	Eastern Mediterranean	No	No	0	9.5[0.0,23.2]	46.9[44.5,49.2]	37.3[30.0,44.7]
Turkey	2003	Europe	No	No	0	16.9[11.7,22.1]	44.8[43.6,46.1]	27.9[25.9,29.9]
Turkmenistan	2016	Europe	No	Yes	0	3.2[0.0,7.7]	38.2[36.4,40.1]	35.0[32.3,37.7]
Turkmenistan	2006	Europe	No	Yes	0	5.5[2.3,8.7]	38.1[36.0,40.2]	32.6[30.1,35.1]
Uganda	2016	Africa	Yes	Yes	1	29.0[26.8,31.1]	37.3[36.2,38.4]	8.3[7.0,9.7]
Uganda	2011	Africa	Yes	Yes	1	13.1[9.7,16.5]	28.8[27.3,30.3]	15.7[13.7,17.7]
Uganda	2006	Africa	Yes	Yes	1	8.3[5.5,11.1]	21.0[19.7,22.4]	12.7[11.0,14.5]
Ukraine	2007	Europe	No	No	0	42.7[28.7,56.7]	50.0[48.3,51.7]	7.3[1.5,13.1]
Ukraine	2005	Europe	No	No	0	40.4[25.6,55.2]	60.4[57.5,63.3]	20.0[12.8,27.2]

© 2020 Li Z et al. *JAMA Network Open.*

eTable 10. Modern contraceptive use among adolescents 15-19 years old and adult women 20-34 years old, and absolute inequality[1] (continued)

Country	Year	WHO region	Income classification[2]	FP2020 country	SSA[3]	15-19 (%)	20-34 (%)	Absolute inequality (percentage points) between 20-34 and 15-19
Uzbekistan	2006	Europe	L	Yes	0	18.5[11.8,25.2]	57.0[55.4,58.6]	38.5[34.5,42.5]
Vanuatu	2007	Western Pacific	LM	No	0	34.5[19.6,49.4]	41.5[38.2,44.8]	7.0[-0.9,14.9]
Vietnam	2013	Western Pacific	LM	Yes	0	29.4[22.0,36.8]	56.4[54.6,58.2]	27.0[22.3,31.7]
Vietnam	2011	Western Pacific	LM	Yes	0	17.8[11.8,23.9]	60.3[58.7,61.8]	42.5[39.1,45.8]
Vietnam	2006	Western Pacific	L	Yes	0	22.2[13.7,30.7]	57.2[55.4,59.0]	35.0[30.8,39.2]
West Bank and Gaza Strip	2014	Eastern Mediterranean	LM	Yes	0	10.1[8.5,11.7]	37.6[36.9,38.3]	27.5[26.4,28.6]
West Bank and Gaza Strip	2010	Eastern Mediterranean	LM	Yes	0	7.8[6.2,9.4]	35.5[34.8,36.2]	27.7[26.9,28.5]
Yemen	2013	Eastern Mediterranean	LM	Yes	0	12.1[9.9,14.3]	29.1[28.2,30.0]	17.0[15.8,18.2]
Yemen	2006	Eastern Mediterranean	L	Yes	0	6.1[3.5,8.7]	19.2[17.3,21.1]	13.1[11.0,15.2]
Zambia	2013	Africa	LM	Yes	1	35.8[31.1,40.5]	48.1[46.9,49.3]	12.3[9.8,14.7]
Zambia	2007	Africa	L	Yes	1	22.0[17.3,26.7]	31.9[30.2,33.6]	9.9[7.2,12.5]
Zimbabwe	2015	Africa	L	Yes	1	44.9[39.3,50.5]	70.9[69.5,72.2]	26.0[23.3,28.6]
Zimbabwe	2014	Africa	L	Yes	1	48.0[43.8,52.2]	69.6[68.0,71.2]	21.6[18.9,24.3]
Zimbabwe	2010	Africa	L	Yes	1	35.4[29.2,41.6]	63.9[62.4,65.4]	28.5[25.5,31.5]
Zimbabwe	2009	Africa	L	Yes	1	38.3[33.7,42.9]	67.6[66.0,69.2]	29.3[26.4,32.2]
Zimbabwe	2005	Africa	L	Yes	1	35.7[28.9,42.5]	64.9[63.3,66.5]	29.2[25.7,32.6]

© 2020 Li Z et al. *JAMA Network Open.*

eTable 11. Unmet need for family planning among adolescents 15-19 years old and adult women 20-34 years old, and absolute inequality[1]

Country	Year	WHO region	Income classification[2]	FP2020 country	SSA[3]	15-19 (%)	20-34 (%)	Absolute inequality (percentage points) between 20-34 and 15-19
Afghanistan	2015	Eastern Mediterranean	L	Yes	0	73.4[67.0,79.8]	61.2[60.2,62.3]	-12.2[-14.8,-9.6]
Afghanistan	2010	Eastern Mediterranean	L	Yes	0	92.9[82.7,100.0]	66.6[62.2,70.9]	-26.3[-33.3,-19.3]
Albania	2017	Europe	UM	No	0	43.9[33.9,53.9]	42.8[40.7,44.9]	-1.1[-4.2,2.0]
Albania	2009	Europe	UM	No	0	23.4[14.7,32.1]	19.0[17.0,21.0]	-4.4[-7.0,-1.8]
Algeria	2012	Africa	UM	No	0	33.3[0.0,87.5]	17.9[14.3,21.4]	-15.5[-42.6,11.7]
Angola	2015	Africa	UM	No	1	83.4[78.2,88.6]	74.3[72.7,75.8]	-9.1[-12.1,-6.2]
Armenia	2016	Europe	LM	No	0	26.7[1.3,52.0]	19.4[17.3,21.4]	-7.3[-14.9,0.3]
Armenia	2010	Europe	LM	No	0	58.5[36.7,80.3]	20.4[18.1,22.6]	-38.1[-45.0,-31.2]
Armenia	2005	Europe	LM	No	0	50.0[27.3,72.7]	24.7[22.4,27.0]	-25.3[-32.2,-18.4]
Azerbaijan	2006	Europe	LM	No	0	72.1[57.5,86.7]	23.5[21.4,25.5]	-48.6[-53.2,-44.1]
Bangladesh	2014	South-East Asia	LM	Yes	0	22.3[19.2,25.4]	15.6[14.8,16.5]	-6.7[-8.5,-4.9]
Bangladesh	2013	South-East Asia	L	Yes	0	30.3[22.4,38.2]	32.1[29.2,35.0]	1.8[-2.9,6.6]
Bangladesh	2011	South-East Asia	L	Yes	0	21.8[19.1,24.5]	17.9[17.0,18.8]	-3.9[-5.5,-2.3]
Bangladesh	2007	South-East Asia	L	Yes	0	31.7[27.2,36.2]	23.5[22.2,24.7]	-8.2[-10.8,-5.7]
Bangladesh	2004	South-East Asia	L	Yes	0	25.3[22.2,28.4]	21.2[20.0,22.4]	-4.1[-6.0,-2.1]
Belize	2016	Americas	UM	No	0	47.1[29.4,64.7]	39.9[32.8,47.0]	-7.2[-18.2,3.8]
Belize	2011	Americas	LM	No	0	56.2[28.9,83.6]	46.4[37.5,55.3]	-9.9[-26.7,7.0]
Benin	2014	Africa	L	Yes	1	79.6[71.7,87.5]	80.8[78.5,83.2]	1.2[-3.5,5.9]
Benin	2012	Africa	L	Yes	1	65.7[61.6,69.8]	73.0[71.5,74.4]	7.3[5.3,9.3]
Benin	2006	Africa	L	Yes	1	76.8[71.5,82.1]	60.1[58.6,61.7]	-16.7[-19.1,-14.2]
Bhutan	2010	South-East Asia	LM	Yes	0	45.5[10.4,80.5]	31.9[25.9,37.9]	-13.5[-30.5,3.5]
Bolivia	2008	Americas	LM	Yes	0	48.0[42.5,53.5]	25.3[24.1,26.6]	-22.7[-25.2,-20.1]
Bolivia	2003	Americas	LM	Yes	0	42.0[35.4,48.6]	28.3[27.1,29.5]	-13.7[-16.6,-10.7]
Bosnia and Herzegovina	2006	Europe	LM	No	0	13.4[0.0,29.9]	46.4[41.4,51.5]	33.0[25.4,40.6]
Burkina Faso	2010	Africa	L	Yes	1	75.8[69.9,81.7]	56.0[54.3,57.6]	-19.8[-22.7,-16.9]
Burkina Faso	2006	Africa	L	Yes	1	78.7[71.5,85.9]	82.2[80.5,84.0]	3.5[-0.7,7.7]
Burkina Faso	2003	Africa	L	Yes	1	74.9[68.5,81.3]	61.9[60.0,63.8]	-13.0[-16.3,-9.8]
Burundi	2016	Africa	L	Yes	1	37.8[28.7,47.0]	49.6[47.9,51.2]	11.7[8.2,15.2]
Burundi	2010	Africa	L	Yes	1	64.9[50.8,79.0]	56.4[54.2,58.7]	-8.5[-14.0,-3.0]

Note:

1. We mark the countries with absolute inequality (difference) between mothers 20-34 and 15-19 years old larger than 5% in orange.
2. Income classification: "L" represents "low-income country"; "LM" represents "lower-middle-income country"; "UM" represents "upper-middle-income country".
3. SSA: "1" represents "Sub-Saharan African country"; "0" represents "Not a Sub-Saharan African country".

© 2020 Li Z et al. *JAMA Network Open.*

eTable 11. Unmet need for family planning among adolescents 15-19 years old and adult women 20-34 years old, and absolute inequality [1] (continued)

Country	Year	WHO region	Income classification[2]	FP2020 country	SSA[3]	15-19 (%)	20-34 (%)	Absolute inequality (percentage points) between 20-34 and 15-19
Cambodia	2014	Western Pacific	L	Yes	0	8.8[2.8,14.8]	15.6[14.6,16.6]	6.8[4.6,9.0]
Cambodia	2010	Western Pacific	L	Yes	0	35.1[25.9,44.3]	22.8[21.7,23.9]	-12.3[-15.3,-9.3]
Cambodia	2005	Western Pacific	L	Yes	0	55.3[45.6,65.0]	38.3[36.7,40.0]	-17.0[-20.5,-13.4]
Cameroon	2014	Africa	LM	Yes	1	39.0[23.4,54.6]	45.6[39.6,51.5]	6.5[-3.3,16.3]
Cameroon	2011	Africa	LM	Yes	1	61.4[55.6,67.2]	47.0[45.3,48.7]	-14.4[-17.5,-11.2]
Cameroon	2004	Africa	L	Yes	1	45.2[39.1,51.3]	40.8[38.8,42.9]	-4.4[-8.0,-0.8]
Central African Republic	2010	Africa	L	Yes	1	82.1[75.7,88.6]	79.2[75.9,82.4]	-3.0[-7.3,1.4]
Central African Republic	2006	Africa	L	Yes	1	56.5[50.5,62.5]	77.1[75.2,79.0]	20.6[17.0,24.2]
Chad	2015	Africa	L	Yes	1	89.9[87.1,92.6]	83.4[81.9,84.9]	-6.5[-8.3,-4.6]
Chad	2010	Africa	L	Yes	1	95.1[91.6,98.7]	93.4[91.7,95.2]	-1.7[-4.1,0.7]
Chad	2004	Africa	L	Yes	1	71.3[62.7,79.9]	61.7[58.6,64.7]	-9.6[-14.6,-4.6]
Colombia	2015	Americas	UM	No	0	18.9[15.5,22.3]	10.8[10.3,11.4]	-8.1[-9.7,-6.4]
Colombia	2010	Americas	UM	No	0	23.4[20.2,26.6]	9.2[8.8,9.6]	-14.2[-15.7,-12.7]
Colombia	2005	Americas	LM	No	0	20.5[16.9,24.1]	12.7[12.0,13.3]	-7.8[-9.5,-6.2]
Comoros	2012	Africa	L	Yes	1	70.7[63.0,78.4]	59.8[56.9,62.6]	-10.9[-15.0,-6.9]
Congo	2015	Africa	LM	Yes	1	61.0[51.5,70.4]	62.8[59.3,66.4]	1.9[-3.9,7.6]
Congo	2011	Africa	LM	Yes	1	49.0[41.2,56.8]	28.3[26.7,29.9]	-20.7[-24.8,-16.7]
Congo	2005	Africa	LM	Yes	1	45.2[36.0,54.4]	23.5[21.7,25.3]	-21.7[-26.6,-16.8]
Costa Rica	2011	Americas	UM	No	0	23.3[7.3,39.4]	16.7[11.3,22.1]	-6.7[-15.9,2.6]
Cuba	2014	Americas	UM	No	0	11.8[0.0,28.8]	17.9[11.9,23.9]	6.1[-2.3,14.5]
Cuba	2010	Americas	UM	No	0	8.2[1.8,14.7]	12.4[8.8,16.1]	4.2[-0.0,8.4]
Côte d'Ivoire	2016	Africa	LM	Yes	1	87.7[78.9,96.5]	80.1[76.9,83.3]	-7.7[-13.0,-2.4]
Côte d'Ivoire	2012	Africa	LM	Yes	1	61.6[57.2,65.9]	58.1[56.1,60.1]	-3.4[-6.1,-0.7]
Democratic Republic of the Congo	2013	Africa	L	Yes	1	70.7[65.0,76.4]	60.9[59.4,62.5]	-9.8[-12.8,-6.8]
Democratic Republic of the Congo	2010	Africa	L	Yes	1	80.8[71.6,90.1]	75.7[72.7,78.8]	-5.1[-10.7,0.5]
Democratic Republic of the Congo	2007	Africa	L	Yes	1	64.3[53.9,74.7]	54.1[52.1,56.2]	-10.2[-15.2,-5.2]
Djibouti	2006	Africa	LM	Yes	1	92.9[77.4,100.0]	84.4[80.8,88.1]	-8.4[-17.2,0.4]
Dominican Republic	2014	Americas	UM	No	0	28.7[23.4,33.9]	23.2[21.0,25.4]	-5.5[-8.7,-2.2]
Dominican Republic	2013	Americas	UM	No	0	33.9[26.2,41.6]	17.7[16.3,19.2]	-16.2[-19.8,-12.5]
Dominican Republic	2007	Americas	LM	No	0	38.0[33.0,43.0]	17.6[16.8,18.5]	-20.4[-22.7,-18.0]

© 2020 Li Z et al. *JAMA Network Open.*

eTable 11. Unmet need for family planning among adolescents 15-19 years old and adult women 20-34 years old, and absolute inequality[1] (continued)

Country	Year	WHO region	Income classification[2]	FP2020 country	SSA[3]	15-19 (%)	20-34 (%)	Absolute inequality (percentage points) between 20-34 and 15-19
Egypt	2014	Eastern Mediterranean	LM	Yes	0	26.4[18.4,34.4]	18.1[17.2,18.9]	-8.3[-10.6,-6.0]
Egypt	2008	Eastern Mediterranean	LM	Yes	0	24.8[17.9,31.7]	16.8[15.8,17.8]	-8.0[-10.2,-5.7]
Egypt	2005	Eastern Mediterranean	LM	Yes	0	24.7[14.6,34.8]	18.3[17.3,19.2]	-6.4[-9.6,-3.3]
El Salvador	2014	Americas	LM	No	0	74.8[66.6,83.0]	49.5[44.4,54.5]	-25.3[-31.4,-19.2]
Ethiopia	2016	Africa	L	Yes	1	49.3[44.0,54.6]	35.4[33.7,37.1]	-13.9[-16.5,-11.2]
Ethiopia	2011	Africa	L	Yes	1	58.0[50.4,65.6]	43.9[42.2,45.6]	-14.1[-17.7,-10.5]
Ethiopia	2005	Africa	L	Yes	1	80.6[75.3,85.9]	61.2[59.3,63.1]	-19.4[-22.3,-16.5]
Gabon	2012	Africa	UM	No	1	62.2[53.3,71.1]	46.7[44.5,48.8]	-15.5[-20.7,-10.4]
Gambia	2013	Africa	L	Yes	1	84.3[75.4,93.2]	74.9[72.7,77.1]	-9.4[-13.7,-5.1]
Ghana	2014	Africa	LM	Yes	1	71.1[60.5,81.7]	49.9[47.7,52.1]	-21.2[-25.7,-16.8]
Ghana	2011	Africa	LM	Yes	1	71.1[55.9,86.2]	59.5[54.9,64.1]	-11.6[-20.7,-2.4]
Ghana	2008	Africa	L	Yes	1	81.9[73.6,90.2]	60.6[57.7,63.5]	-21.3[-25.5,-17.0]
Ghana	2003	Africa	L	Yes	1	87.1[81.7,92.5]	58.0[55.3,60.7]	-29.1[-32.4,-25.8]
Guatemala	2015	Americas	LM	No	0	35.4[32.2,38.6]	21.3[20.3,22.3]	-14.1[-15.8,-12.5]
Guinea	2012	Africa	L	Yes	1	89.2[84.9,93.5]	72.2[69.7,74.6]	-17.0[-20.0,-14.0]
Guinea	2005	Africa	L	Yes	1	69.6[62.6,76.6]	64.6[61.7,67.5]	-5.0[-9.1,-0.9]
Guinea-Bissau	2014	Africa	L	Yes	1	100.0[100.0,100.0]	72.3[65.0,79.6]	-27.7[-33.8,-21.6]
Guinea-Bissau	2006	Africa	L	Yes	1	81.7[76.0,87.4]	86.6[84.8,88.4]	4.9[1.4,8.4]
Guyana	2014	Americas	LM	No	0	67.6[51.7,83.4]	46.4[40.2,52.6]	-21.2[-30.9,-11.5]
Guyana	2009	Americas	LM	No	0	54.0[42.5,65.5]	40.8[38.0,43.6]	-13.2[-18.7,-7.7]
Guyana	2006	Americas	LM	No	0	63.1[51.6,74.6]	64.0[59.0,69.0]	0.9[-6.7,8.5]
Haiti	2017	Americas	L	Yes	0	69.4[65.5,73.3]	52.7[51.1,54.3]	-16.7[-18.8,-14.5]
Haiti	2012	Americas	L	Yes	0	68.8[62.3,75.3]	50.5[48.9,52.1]	-18.3[-21.5,-15.2]
Haiti	2006	Americas	L	Yes	0	62.8[58.6,67.1]	53.6[51.7,55.5]	-9.3[-11.7,-6.8]
Honduras	2012	Americas	LM	Yes	0	24.0[21.4,26.5]	13.8[13.0,14.7]	-10.1[-11.5,-8.8]
Honduras	2006	Americas	LM	Yes	0	37.8[34.4,41.3]	24.3[23.2,25.4]	-13.5[-15.4,-11.6]
India	2015	South-East Asia	LM	Yes	0	64.5[63.3,65.7]	29.8[29.6,30.1]	-34.7[-35.1,-34.2]
India	2006	South-East Asia	L	Yes	0	23.9[23.0,24.9]	16.2[15.9,16.6]	-7.7[-8.2,-7.2]

© 2020 Li Z et al. *JAMA Network Open.*

eTable 11. Unmet need for family planning among adolescents 15-19 years old and adult women 20-34 years old, and absolute inequality[1] (continued)

Country	Year	WHO region	Income classification[2]	FP2020 country	SSA[3]	15-19 (%)	20-34 (%)	Absolute inequality (percentage points) between 20-34 and 15-19
Indonesia	2017	South-East Asia	LM	Yes	0	12.5[8.7,16.3]	14.4[13.7,15.0]	1.9[0.4,3.3]
Indonesia	2007	South-East Asia	LM	Yes	0	17.3[12.3,22.3]	15.6[14.9,16.2]	-1.7[-3.6,0.2]
Indonesia	2002	South-East Asia	L	Yes	0	12.6[7.9,17.3]	15.2[14.5,15.9]	2.6[0.7,4.5]
Iraq	2011	Eastern Mediterranean	LM	Yes	0	34.6[15.0,54.2]	27.6[24.6,30.5]	-7.0[-18.4,4.3]
Iraq	2006	Eastern Mediterranean	LM	Yes	0	37.5[31.5,43.5]	53.0[51.1,54.9]	15.5[11.8,19.3]
Jordan	2012	Eastern Mediterranean	UM	No	0	27.1[9.9,44.3]	15.9[14.7,17.0]	-11.2[-15.1,-7.4]
Jordan	2007	Eastern Mediterranean	LM	No	0	24.4[6.8,42.0]	23.2[21.9,24.6]	-1.2[-5.3,2.9]
Kazakhstan	2010	Europe	UM	No	0	80.0[24.5,100.0]	33.3[21.7,45.0]	-46.7[-76.0,-17.4]
Kenya	2014	Africa	LM	Yes	1	35.3[26.5,44.1]	14.7[13.9,15.5]	-20.6[-24.3,-17.0]
Kenya	2009	Africa	L	Yes	1	59.7[52.3,67.0]	37.9[35.9,40.0]	-21.7[-25.4,-18.1]
Kenya	2003	Africa	L	Yes	1	60.3[52.6,68.0]	42.1[40.0,44.3]	-18.2[-22.0,-14.3]
Kyrgyzstan	2012	Europe	L	Yes	0	65.2[49.0,81.4]	35.6[33.2,37.9]	-29.6[-34.6,-24.7]
Lao People's Democratic Republic	2011	Western Pacific	LM	Yes	0	71.9[55.4,88.3]	66.2[60.4,71.9]	-5.7[-16.0,4.6]
Lesotho	2014	Africa	LM	Yes	1	42.3[33.5,51.1]	21.5[19.6,23.3]	-20.8[-25.2,-16.5]
Lesotho	2009	Africa	LM	Yes	1	51.0[42.9,59.1]	33.1[31.1,35.1]	-17.9[-22.2,-13.6]
Lesotho	2004	Africa	L	Yes	1	70.4[62.7,78.1]	43.3[40.9,45.7]	-27.1[-31.4,-22.9]
Liberia	2013	Africa	L	Yes	1	77.6[69.6,85.6]	58.2[56.2,60.2]	-19.4[-23.7,-15.1]
Liberia	2007	Africa	L	Yes	1	88.6[83.2,94.0]	71.6[69.5,73.8]	-17.0[-20.2,-13.7]
Madagascar	2009	Africa	L	Yes	1	54.7[51.5,57.9]	28.8[27.4,30.2]	-25.9[-27.9,-23.9]
Madagascar	2004	Africa	L	Yes	1	57.4[51.2,63.5]	37.1[34.9,39.4]	-20.2[-23.6,-16.8]
Malawi	2015	Africa	L	Yes	1	35.7[31.4,40.0]	24.1[23.1,25.0]	-11.6[-13.8,-9.4]
Malawi	2013	Africa	L	Yes	1	36.2[29.8,42.7]	35.7[33.4,37.9]	-0.6[-4.5,3.4]
Malawi	2010	Africa	L	Yes	1	46.4[41.6,51.2]	36.4[35.3,37.5]	-10.0[-12.4,-7.6]
Malawi	2004	Africa	L	Yes	1	56.4[50.7,62.1]	50.6[48.9,52.2]	-5.8[-8.9,-2.7]
Maldives	2009	South-East Asia	LM	No	0	63.4[50.0,76.8]	44.4[42.7,46.2]	-19.0[-22.9,-15.0]
Mali	2015	Africa	L	Yes	1	85.2[77.7,92.8]	82.3[79.6,84.9]	-3.0[-7.6,1.7]
Mali	2012	Africa	L	Yes	1	75.7[69.5,81.9]	68.6[66.6,70.7]	-7.1[-10.5,-3.7]
Mali	2009	Africa	L	Yes	1	89.7[83.1,96.2]	81.9[78.8,84.9]	-7.8[-12.2,-3.3]
Mali	2006	Africa	L	Yes	1	82.2[78.3,86.1]	73.6[71.8,75.3]	-8.6[-11.1,-6.2]

© 2020 Li Z et al. *JAMA Network Open.*

eTable 11. Unmet need for family planning among adolescents 15-19 years old and adult women 20-34 years old, and absolute inequality[1] (continued)

Country	Year	WHO region	Income classification[2]	FP2020 country	SSA[3]	15-19 (%)	20-34 (%)	Absolute inequality (percentage points) between 20-34 and 15-19
Mauritania	2015	Africa	LM	Yes	1	72.7[60.6,84.9]	78.1[74.6,81.5]	5.3[-1.9,12.5]
Mauritania	2011	Africa	L	Yes	1	85.5[76.5,94.5]	78.0[74.2,81.7]	-7.5[-13.4,-1.6]
Mexico	2015	Americas	UM	No	0	32.1[24.1,40.1]	26.5[23.1,30.0]	-5.5[-10.4,-0.7]
Moldova	2012	Europe	LM	No	0	40.0[0.0,100.0]	22.5[9.0,36.0]	-17.5[-52.9,17.9]
Moldova	2005	Europe	LM	No	0	17.2[8.4,26.0]	13.6[12.1,15.1]	-3.6[-6.9,-0.3]
Mongolia	2013	Western Pacific	LM	Yes	0	66.7[0.0,100.0]	54.1[44.6,63.5]	-12.6[-80.1,54.8]
Mongolia	2005	Western Pacific	L	Yes	0	7.1[0.0,24.5]	30.0[10.8,49.2]	22.9[4.1,41.7]
Morocco	2003	Eastern Mediterranean	LM	No	0	17.9[10.6,25.2]	14.6[13.4,15.9]	-3.3[-5.8,-0.7]
Mozambique	2011	Africa	L	Yes	1	78.8[73.8,83.8]	56.5[54.5,58.4]	-22.3[-25.3,-19.3]
Mozambique	2003	Africa	L	Yes	1	45.5[38.6,52.4]	37.5[35.6,39.3]	-8.0[-12.0,-4.1]
Myanmar	2016	South-East Asia	LM	Yes	0	30.3[23.2,37.4]	21.8[20.2,23.4]	-8.5[-11.0,-6.0]
Namibia	2013	Africa	UM	No	1	43.2[29.8,56.6]	20.8[19.0,22.5]	-22.4[-28.5,-16.3]
Namibia	2007	Africa	LM	No	1	30.1[25.2,35.0]	23.9[22.1,25.7]	-6.2[-9.0,-3.5]
Nepal	2016	South-East Asia	L	Yes	0	61.1[56.6,65.6]	38.7[37.2,40.2]	-22.4[-24.7,-20.1]
Nepal	2014	South-East Asia	L	Yes	0	72.7[52.5,92.9]	57.6[49.2,65.9]	-15.2[-28.2,-2.2]
Nepal	2011	South-East Asia	L	Yes	0	69.8[64.6,75.0]	40.2[38.7,41.8]	-29.6[-32.1,-27.1]
Nepal	2006	South-East Asia	L	Yes	0	70.4[66.1,74.7]	29.7[28.4,31.1]	-40.7[-42.8,-38.5]
Niger	2012	Africa	L	Yes	1	64.8[57.9,71.7]	48.9[46.7,51.1]	-15.9[-19.5,-12.3]
Niger	2006	Africa	L	Yes	1	74.1[68.3,79.9]	53.7[51.1,56.3]	-20.4[-23.9,-16.9]
Nigeria	2017	Africa	LM	Yes	1	95.3[90.8,99.9]	83.3[81.2,85.4]	-12.1[-15.0,-9.1]
Nigeria	2013	Africa	LM	Yes	1	84.7[80.7,88.7]	50.1[48.8,51.4]	-34.6[-36.6,-32.6]
Nigeria	2011	Africa	LM	Yes	1	87.5[78.6,96.4]	74.5[71.3,77.7]	-13.0[-18.3,-7.7]
Nigeria	2008	Africa	LM	Yes	1	86.4[82.9,89.9]	57.0[55.7,58.4]	-29.4[-31.3,-27.4]
Nigeria	2007	Africa	L	Yes	1	80.4[74.2,86.6]	87.7[86.6,88.8]	7.3[4.1,10.5]
Nigeria	2003	Africa	L	Yes	1	77.1[65.9,88.3]	55.2[52.2,58.2]	-21.9[-27.6,-16.2]
Pakistan	2012	Eastern Mediterranean	LM	Yes	0	59.5[49.5,69.5]	42.1[40.6,43.7]	-17.4[-20.7,-14.0]
Pakistan	2006	Eastern Mediterranean	L	Yes	0	75.0[67.1,82.9]	53.8[51.9,55.6]	-21.2[-24.3,-18.1]
Panama	2013	Americas	UM	No	0	49.5[39.7,59.3]	40.7[36.5,44.9]	-8.8[-15.2,-2.5]
Paraguay	2016	Americas	UM	No	0	17.4[6.0,28.8]	18.3[13.5,23.1]	0.9[-6.1,8.0]

eTable 11. Unmet need for family planning among adolescents 15-19 years old and adult women 20-34 years old, and absolute inequality[1] (continued)

Country	Year	WHO region	Income classification[2]	FP2020 country	SSA[3]	15-19 (%)	20-34 (%)	Absolute inequality (percentage points) between 20-34 and 15-19
Peru	2012	Americas	UM	No	0	19.5[15.1,23.9]	12.3[11.6,13.1]	-7.2[-9.1,-5.2]
Peru	2011	Americas	UM	No	0	18.3[13.2,23.4]	12.9[12.0,13.7]	-5.4[-7.6,-3.3]
Peru	2010	Americas	UM	No	0	15.6[11.3,19.9]	14.4[13.5,15.2]	-1.2[-3.1,0.7]
Peru	2009	Americas	UM	No	0	19.0[14.3,23.7]	14.8[14.0,15.6]	-4.2[-6.2,-2.1]
Peru	2008	Americas	UM	No	0	17.5[11.4,23.6]	15.8[15.1,16.5]	-1.7[-4.2,0.8]
Peru	2006	Americas	LM	No	0	16.6[6.7,26.5]	15.8[15.1,16.5]	-0.8[-4.8,3.2]
Philippines	2017	Western Pacific	LM	Yes	0	45.3[39.8,50.8]	23.1[22.0,24.2]	-22.2[-24.4,-20.1]
Philippines	2013	Western Pacific	LM	Yes	0	40.6[33.5,47.7]	24.7[23.2,26.1]	-15.9[-18.7,-13.2]
Philippines	2008	Western Pacific	LM	Yes	0	58.0[50.4,65.6]	30.9[29.3,32.5]	-27.1[-29.9,-24.2]
Philippines	2003	Western Pacific	LM	Yes	0	51.0[42.5,59.5]	34.1[32.4,35.7]	-16.9[-20.0,-13.9]
Rwanda	2015	Africa	L	Yes	1	59.3[49.1,69.6]	27.5[26.0,29.1]	-31.8[-35.3,-28.3]
Rwanda	2010	Africa	L	Yes	1	16.2[0.0,33.6]	28.0[26.4,29.6]	11.8[6.5,17.1]
Rwanda	2005	Africa	L	Yes	1	87.5[75.4,99.6]	69.8[67.7,71.8]	-17.7[-21.7,-13.7]
Sao Tome and Principe	2014	Africa	LM	Yes	1	48.6[31.2,66.0]	45.7[38.7,52.7]	-2.9[-14.4,8.6]
Sao Tome and Principe	2008	Africa	LM	Yes	1	68.5[58.0,79.0]	43.9[40.5,47.2]	-24.6[-30.1,-19.2]
Senegal	2017	Africa	L	Yes	1	76.1[71.8,80.4]	49.2[47.5,51.0]	-26.9[-29.3,-24.5]
Senegal	2016	Africa	L	Yes	1	81.4[76.1,86.7]	52.3[49.9,54.8]	-29.1[-32.2,-26.0]
Senegal	2015	Africa	L	Yes	1	78.7[72.9,84.5]	56.0[53.6,58.4]	-22.7[-25.9,-19.4]
Senegal	2014	Africa	LM	Yes	1	62.5[45.4,79.6]	62.4[60.7,64.1]	-0.1[-7.5,7.4]
Senegal	2013	Africa	LM	Yes	1	87.9[83.2,92.6]	65.7[63.4,68.1]	-22.2[-25.1,-19.3]
Senegal	2011	Africa	LM	Yes	1	85.3[81.8,88.8]	72.9[71.2,74.6]	-12.4[-14.6,-10.2]
Senegal	2005	Africa	L	Yes	1	86.9[83.0,90.8]	75.9[74.2,77.6]	-11.0[-13.3,-8.7]
Serbia	2014	Europe	UM	No	0	50.0[0.0,100.0]	34.0[20.0,48.1]	-16.0[-252.6,220.6]
Serbia	2005	Europe	UM	No	0	46.6[28.4,64.8]	55.1[50.6,59.6]	8.5[-0.5,17.4]
Sierra Leone	2013	Africa	L	Yes	1	75.9[70.3,81.5]	50.2[48.5,51.9]	-25.7[-28.8,-22.6]
Sierra Leone	2010	Africa	L	Yes	1	92.2[84.5,99.8]	89.5[85.8,93.2]	-2.7[-7.8,2.4]
Sierra Leone	2008	Africa	L	Yes	1	94.9[91.2,98.6]	71.5[69.1,73.8]	-23.4[-26.1,-20.8]
Somalia	2006	Africa	L	Yes	1	89.6[84.4,94.8]	78.0[75.4,80.5]	-11.7[-15.3,-8.0]
Sudan	2014	Africa	LM	Yes	1	78.8[64.1,93.5]	78.0[74.7,81.3]	-0.8[-9.2,7.5]
Suriname	2010	Americas	UM	No	0	63.0[43.5,82.4]	59.2[51.0,67.3]	-3.8[-16.2,8.6]
Suriname	2006	Americas	LM	No	0	36.5[22.2,50.8]	55.7[50.3,61.0]	19.2[10.3,28.0]

© 2020 Li Z et al. *JAMA Network Open.*

eTable 11. Unmet need for family planning among adolescents 15-19 years old and adult women 20-34 years old, and absolute inequality[1] (continued)

Country	Year	WHO region	Income classification[2]	FP2020 country	SSA[3]	15-19 (%)	20-34 (%)	Absolute inequality (percentage points) between 20-34 and 15-19
Swaziland	2014	Africa	LM	No	1	38.5[7.9,69.1]	15.6[9.9,21.3]	-22.8[-40.9,-4.7]
Swaziland	2010	Africa	LM	No	1	26.7[1.3,52.0]	21.2[15.2,27.2]	-5.5[-20.5,9.6]
Syrian Arab Republic	2006	Eastern Mediterranean	LM	No	0	36.6[28.8,44.4]	49.4[47.0,51.8]	12.8[8.0,17.6]
Tajikistan	2017	Europe	LM	Yes	0	81.2[67.0,95.5]	49.5[47.4,51.5]	-31.8[-35.8,-27.7]
Tajikistan	2012	Europe	L	Yes	0	85.2[76.9,93.5]	52.5[50.2,54.8]	-32.7[-35.8,-29.6]
Tajikistan	2005	Europe	L	Yes	0	67.5[53.0,82.0]	65.2[62.1,68.2]	-2.3[-11.2,6.5]
Tanzania	2015	Africa	L	Yes	1	59.5[52.8,66.2]	36.8[35.1,38.6]	-22.7[-26.0,-19.3]
Tanzania	2010	Africa	L	Yes	1	51.8[40.7,62.9]	40.6[38.7,42.5]	-11.2[-16.1,-6.2]
Tanzania	2004	Africa	L	Yes	1	64.1[55.9,72.3]	48.2[46.2,50.2]	-15.9[-20.0,-11.8]
Thailand	2012	South-East Asia	UM	No	0	24.4[15.4,33.5]	12.7[9.4,16.1]	-11.7[-16.9,-6.5]
Timor-Leste	2016	South-East Asia	LM	Yes	0	71.4[61.6,81.3]	50.0[48.0,52.1]	-21.4[-24.8,-18.0]
Timor-Leste	2009	South-East Asia	LM	Yes	0	77.4[69.4,85.4]	59.6[57.5,61.6]	-17.8[-21.0,-14.7]
Togo	2014	Africa	L	Yes	1	68.4[63.2,73.5]	60.2[58.2,62.2]	-8.1[-10.8,-5.5]
Togo	2010	Africa	L	Yes	1	94.6[87.0,100.0]	82.1[78.5,85.8]	-12.5[-17.5,-7.4]
Togo	2006	Africa	L	Yes	1	73.6[65.4,81.8]	80.9[78.8,83.0]	7.3[2.7,11.9]
Turkey	2003	Europe	LM	No	0	22.8[11.0,34.6]	11.0[9.8,12.3]	-11.8[-15.6,-7.9]
Turkmenistan	2006	Europe	LM	No	0	92.3[81.3,100.0]	66.3[62.1,70.4]	-26.0[-33.0,-19.1]
Uganda	2016	Africa	L	Yes	1	58.8[55.0,62.6]	42.3[40.9,43.6]	-16.5[-18.6,-14.4]
Uganda	2011	Africa	L	Yes	1	67.8[59.9,75.7]	52.1[50.0,54.2]	-15.7[-19.7,-11.8]
Uganda	2006	Africa	L	Yes	1	74.8[68.1,81.5]	60.3[58.3,62.4]	-14.5[-18.0,-11.0]
Ukraine	2007	Europe	LM	No	0	36.7[19.5,53.9]	11.6[10.1,13.1]	-25.1[-32.1,-18.2]
Uzbekistan	2006	Europe	L	Yes	0	30.9[13.9,47.9]	34.7[32.5,36.9]	3.8[-5.9,13.4]
Vietnam	2011	Western Pacific	LM	Yes	0	50.0[5.3,94.7]	26.7[19.5,34.0]	-23.3[-46.7,0.1]
Yemen	2013	Eastern Mediterranean	LM	Yes	0	67.6[62.5,72.7]	46.4[45.2,47.7]	-21.2[-23.5,-18.8]
Zambia	2013	Africa	LM	Yes	1	38.6[32.3,44.9]	30.0[28.7,31.4]	-8.6[-11.7,-5.4]
Zambia	2007	Africa	L	Yes	1	44.2[34.9,53.5]	37.2[35.1,39.3]	-7.0[-11.7,-2.4]
Zimbabwe	2015	Africa	L	Yes	1	21.2[14.7,27.7]	12.6[11.4,13.8]	-8.6[-11.6,-5.6]
Zimbabwe	2014	Africa	L	Yes	1	15.2[7.1,23.3]	21.2[17.9,24.5]	6.0[0.8,11.2]
Zimbabwe	2010	Africa	L	Yes	1	31.5[23.0,40.0]	18.1[16.7,19.5]	-13.4[-17.3,-9.4]
Zimbabwe	2005	Africa	L	Yes	1	31.6[23.3,39.9]	16.5[15.0,17.9]	-15.1[-19.2,-11.1]

© 2020 Li Z et al. *JAMA Network Open.*

eFigure 2. Relative inequality in contraception use and unmet need between adolescents 15-19 years old and adult women 20-34 years old, the latest years

A) Modern contraceptive use

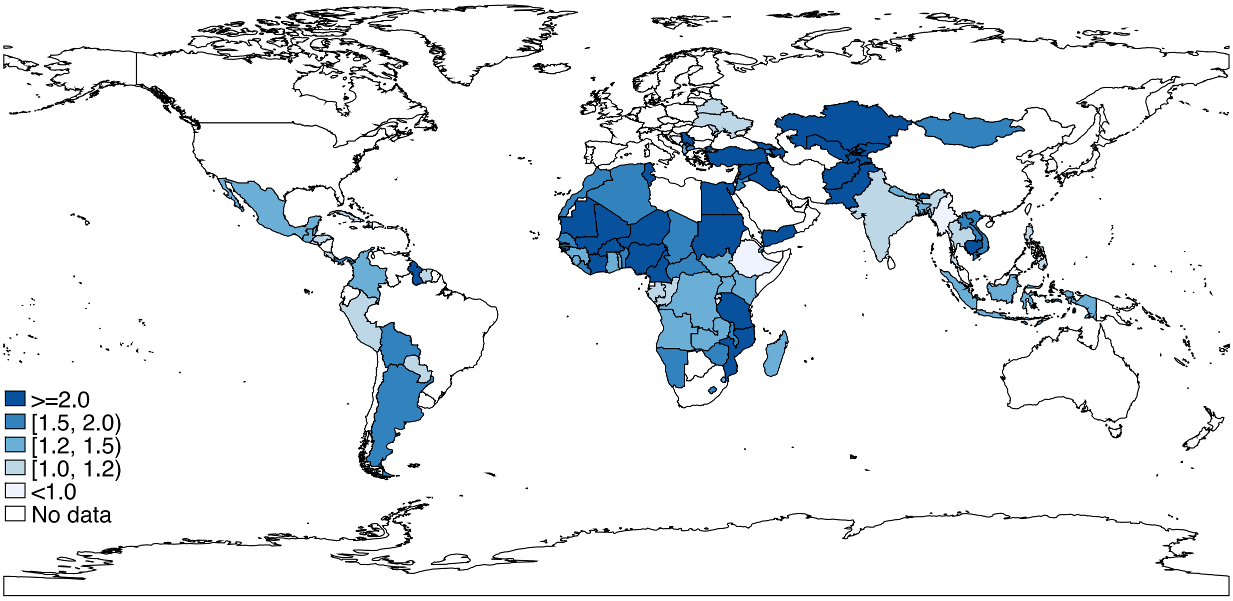

B) Unmet need for family planning

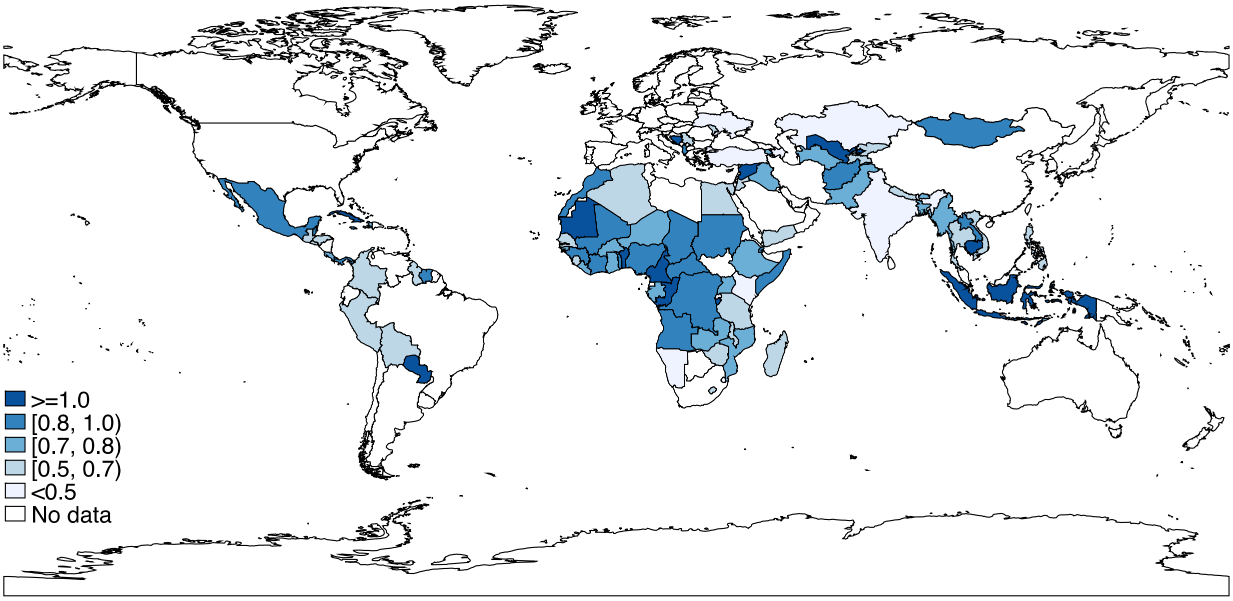

© 2020 Li Z et al. *JAMA Network Open.*

eTable 12. Changes in absolute inequality of modern contraceptive use(MC) between adult women and adolescents at country-level, the latest year Vs. the earliest year[1]

Country	Absolute inequality in MC, latest year (percentage points)	Absolute inequality in MC, earliest year (percentage points)	Change in absolute inequality[2]			Absolute annual change (percentage points)
			Value (percentage points)	P value	Rank in change[3]	
Namibia	30.6	4.0	26.6	0.000	1	4.4
Swaziland	23.0	5.5	17.4	0.000	2	2.2
Cameroon	13.2	-1.5	14.7	0.007	3	1.5
Malawi	25.7	14.2	11.5	0.004	4	1.0
Côte d'Ivoire	7.6	-3.7	11.3	0.002	5	1.1
Mozambique	12.4	1.5	11.0	0.006	6	1.4
Mongolia	14.5	4.2	10.3	0.004	7	1.3
Guinea-Bissau	10.1	-0.1	10.2	0.006	8	0.7
Cambodia	23.3	16.4	6.9	0.018	9	0.8
Kazakhstan	28.5	21.8	6.7	0.038	10	0.7
Guyana	25.3	18.7	6.6	0.029	11	0.8
Mali	7.9	1.5	6.4	0.035	12	0.7
Lesotho	32.0	26.5	5.5	0.011	13	0.5
Tanzania	21.2	16.0	5.2	0.010	14	0.5
Burkina Faso	11.6	6.4	5.2	0.011	15	0.7
Central African Republic	5.2	0.2	5.0	0.046	16	0.5
Togo	5.0	0.3	4.7	0.026	17	0.6
Senegal	9.6	5.4	4.2	0.027	18	0.3
Yemen	17.0	13.1	3.9	0.099	19	0.6
Guinea	2.6	-1.2	3.8	0.361	20	0.3
Nigeria	8.0	4.7	3.2	0.473	21	0.2
Turkmenistan	35.0	32.6	2.4	0.167	22	0.2
Zambia	12.3	9.9	2.4	0.215	23	0.4
Pakistan	24.1	21.7	2.4	0.062	24	0.4
Honduras	9.3	7.2	2.0	0.052	25	0.3
Sierra Leone	6.1	4.3	1.8	0.590	26	0.2
Chad	1.2	0.0	1.2	0.637	27	0.1
Afghanistan	12.2	11.3	0.9	0.934	28	0.2
Haiti	1.4	0.5	0.9	0.302	29	0.1
Thailand	5.1	4.3	0.8	0.855	30	0.1
Benin	3.9	3.7	0.2	0.713	31	0.0
Bosnia and Herzegovina	-0.5	-0.7	0.2	0.909	32	0.0

Note:

1. We keep countries with the latest survey conducted in 2010 or later. The time elapsed between two surveys must be five years or more.
2. "Change in absolute inequality" is "Absolute inequality in MC, latest year" minus "Absolute inequality in MC, earliest year".
3. "Rank in change" is the rank of "change in absolute inequality". The larger the "change in absolute inequality", the higher the rank is. "1" represents the largest increase in absolute inequality over year.

© 2020 Li Z et al. *JAMA Network Open.*

eTable 12. Changes in absolute inequality of modern contraceptive use(MC) between adult women and adolescents at country-level, the latest year Vs. the earliest year (continued) [1]

Country	Absolute inequality in MC, latest year (percentage points)	Absolute inequality in MC, earliest year (percentage points)	Change in absolute inequality[2]			Absolute annual change (percentage points)
			Value (percentage points)	P value	Rank in change[3]	
Iraq	20.4	20.5	-0.1	0.602	33	0.0
Niger	6.5	6.6	-0.1	0.840	34	0.0
Liberia	8.6	8.8	-0.3	0.909	35	0.0
Peru	3.1	3.9	-0.9	0.751	36	-0.1
Indonesia	13.0	14.0	-1.0	0.390	37	-0.1
Lao People's Democratic Republic	21.5	22.6	-1.1	0.990	38	-0.2
Democratic Republic of the Congo	1.5	2.8	-1.3	0.745	39	-0.2
Kyrgyzstan	28.7	30.4	-1.7	0.954	40	-0.2
Rwanda	5.8	7.7	-1.9	0.790	41	-0.2
Congo	2.0	4.7	-2.6	0.067	42	-0.3
Zimbabwe	26.0	29.2	-3.2	0.113	43	-0.3
Moldova	7.1	10.3	-3.2	0.068	44	-0.5
Egypt	34.6	38.1	-3.5	0.200	45	-0.4
Uganda	8.3	12.7	-4.4	0.028	46	-0.4
Jordan	21.5	25.9	-4.4	0.004	47	-0.9
The former Yugoslav Republic of Macedonia	4.8	9.3	-4.5	0.008	48	-0.8
Tajikistan	20.0	24.6	-4.6	0.010	49	-0.4
Ghana	7.5	12.3	-4.8	0.003	50	-0.4
Serbia	5.4	10.8	-5.4	0.035	51	-0.6
Sao Tome and Principe	12.5	18.1	-5.6	0.003	52	-0.9
India	6.7	12.8	-6.1	0.026	53	-0.7
Myanmar	-1.0	5.2	-6.2	0.032	54	-0.9
Albania	1.1	8.3	-7.2	0.020	55	-0.6
Bangladesh	10.8	18.0	-7.2	0.014	56	-0.7
Vietnam	27.0	35.0	-8.0	0.017	57	-1.1
Armenia	7.1	15.1	-8.0	0.002	58	-0.7
Mauritania	10.4	18.7	-8.3	0.010	59	-1.0
Lebanon	23.5	31.9	-8.4	0.005	60	-1.4
Belarus	3.5	12.5	-9.0	0.007	61	-1.3
Kenya	15.7	25.0	-9.3	0.010	62	-0.8
Timor-Leste	7.3	17.0	-9.7	0.004	63	-1.4
Ethiopia	-1.0	8.7	-9.7	0.000	64	-0.9
Belize	16.7	26.6	-9.9	0.004	65	-1.0
Burundi	-4.5	5.6	-10.1	0.000	66	-0.9
Colombia	13.7	24.0	-10.3	0.000	67	-1.0
Cuba	10.9	21.8	-10.9	0.000	68	-1.4
Algeria	18.6	31.8	-13.2	0.000	69	-2.2
Dominican Republic	12.6	30.7	-18.1	0.000	70	-1.3
Nepal	15.1	37.1	-22.1	0.000	71	-2.2
Philippines	0.7	25.6	-24.9	0.000	72	-1.8

© 2020 Li Z et al. *JAMA Network Open.*

e Table 13. Changes in absolute inequality of unmet need for family planning (unmet_FP) between adult women and adolescents at country-level, the latest year Vs. the earliest year[1]

Country	Absolute inequality in unmet_FP, latest year (percentage points)	Absolute inequality in unmet_FP, earliest year (percentage points)	Change in absolute inequality[2]			Absolute annual change (percentage points)
			Value (percentage points)	P value	Rank in change[3]	
Mongolia	-12.60	22.90	-35.50	0.000	1	-4.4
Guinea-Bissau	-27.7	4.9	-32.6	0.000	2	-4.1
Tajikistan	-31.8	-2.3	-29.4	0.000	3	-2.5
India	-34.7	-7.7	-27.0	0.000	4	-3.0
Serbia	-16.0	8.5	-24.5	0.000	5	-2.7
Iraq	-7.0	15.5	-22.6	0.000	6	-4.5
Guyana	-21.2	0.9	-22.0	0.000	7	-2.8
Namibia	-22.4	-6.2	-16.2	0.001	8	-2.7
Senegal	-26.9	-11.0	-15.8	0.005	9	-1.3
Togo	-8.1	7.3	-15.4	0.007	10	-1.9
Mozambique	-22.3	-8.0	-14.3	0.050	11	-1.8
Rwanda	-31.8	-17.7	-14.1	0.012	12	-1.4
Moldova	-17.5	-3.6	-13.9	0.039	13	-2.0
Swaziland	-22.8	-9.6	-13.3	0.006	14	-1.7
Guinea	-17.0	-5.0	-12.0	0.044	15	-1.7
Jordan	-11.2	-1.2	-10.1	0.044	16	-2.0
Haiti	-16.7	-9.3	-7.4	0.006	17	-0.7
Burkina Faso	-19.8	-13.0	-6.8	0.035	18	-1.0
Tanzania	-22.7	-15.9	-6.8	0.006	19	-0.6
Peru	-7.2	-0.8	-6.4	0.000	20	-1.1
Malawi	-11.6	-5.8	-5.8	0.047	21	-0.5
Philippines	-22.2	-16.9	-5.3	0.032	22	-0.4
Timor-Leste	-21.4	-17.8	-3.5	0.197	23	-0.5
Bangladesh	-6.7	-4.1	-2.6	0.051	24	-0.3
Kenya	-20.6	-18.2	-2.5	0.372	25	-0.2
Liberia	-19.4	-17.0	-2.4	0.292	26	-0.4
Sierra Leone	-25.7	-23.4	-2.2	0.331	27	-0.4
Uganda	-16.5	-14.5	-2.1	0.379	28	-0.2
Egypt	-8.3	-6.4	-1.9	0.376	29	-0.2
Zambia	-8.6	-7.0	-1.5	0.557	30	-0.3
Indonesia	1.9	2.6	-0.7	0.483	31	-0.1
Colombia	-8.1	-7.8	-0.2	0.319	32	0.0

Note:

1. We keep countries with the latest survey conducted in 2010 or later. The time elapsed between two surveys must be five years or more.
2. "Change in absolute inequality" is "Absolute inequality in unmet_FP, latest year" minus "Absolute inequality in unmet_FP, earliest year".
3. "Rank in change" is the rank of "change in absolute inequality". The larger the "change in absolute inequality", the higher the rank is. "1" represents the largest increase in absolute inequality over year.

© 2020 Li Z et al. *JAMA Network Open.*

eTable 13. Changes in absolute inequality of unmet need for family planning (unmet_FP) between adult women and adolescents at country-level, the latest year Vs. the earliest year[1]

Country	Absolute inequality in unmet_FP, latest year (percentage points)	Absolute inequality in unmet_FP, earliest year (percentage points)	Change in absolute inequality[2]			Absolute annual change (percentage points)
			Value (percentage points)	P value	Rank in change[3]	
Democratic Republic of the Congo	-9.8	-10.2	0.4	0.583	33	0.1
Belize	-7.2	-9.9	2.7	0.239	34	0.5
Chad	-6.5	-9.6	3.1	0.056	35	0.3
Albania	-1.1	-4.4	3.3	0.052	36	0.4
Honduras	-10.1	-13.5	3.3	0.327	37	0.6
Pakistan	-17.4	-21.2	3.9	0.453	38	0.6
Niger	-15.9	-20.4	4.5	0.038	39	0.7
Ethiopia	-13.9	-19.4	5.5	0.011	40	0.5
Mali	-3.0	-8.6	5.6	0.032	41	0.6
Lesotho	-20.8	-27.1	6.3	0.029	42	0.6
Zimbabwe	-8.6	-15.1	6.5	0.033	43	0.7
Ghana	-21.2	-29.1	7.9	0.018	44	0.7
Nigeria	-12.1	-21.9	9.9	0.018	45	0.7
Cameroon	6.5	-4.4	10.9	0.064	46	1.1
Afghanistan	-12.2	-26.3	14.1	0.007	47	2.8
Dominican Republic	-5.5	-20.4	14.9	0.004	48	2.1
Benin	1.2	-16.7	17.9	0.000	49	2.2
Armenia	-7.3	-25.3	18.0	0.000	50	1.6
Nepal	-22.4	-40.7	18.3	0.000	51	1.8
Burundi	11.7	-8.5	20.2	0.051	52	3.4
Sao Tome and Principe	-2.9	-24.6	21.7	0.000	53	3.6
Congo	1.9	-21.7	23.6	0.000	54	2.4
Cambodia	6.8	-17.0	23.8	0.000	55	2.6

© 2020 Li Z et al. *JAMA Network Open.*
